# Insights into the Structure, Function, and Ion-Mediated Signaling Pathways Transduced by Plant Integrin-Linked Kinases

**DOI:** 10.3389/fpls.2017.00376

**Published:** 2017-04-03

**Authors:** Sorina C. Popescu, Elizabeth K. Brauer, Gizem Dimlioglu, George V. Popescu

**Affiliations:** ^1^Department of Biochemistry, Molecular Biology, Plant Pathology, and Entomology, Mississippi State University, StarkvilleMS, USA; ^2^Ottawa Research and Development Center, Agriculture and Agri-Food Canada, OttawaON, Canada; ^3^Institute for Genomics, Biocomputing and Biotechnology, Mississippi State University, StarkvilleMS, USA

**Keywords:** manganese, potassium, integrin-linked kinases, plant immune response, plant abiotic response, calcium, transporters

## Abstract

Kinases facilitate detection of extracellular signals and set in motion cellular responses for plant adaptation and survival. Some of the energy utilized for kinase signal processing is produced through the activity of ion transporters. Additionally, the synergy between cellular ions and signal transduction influences plant response to pathogens, and their growth and development. In plants, the signaling elements that connect cell wall and membrane sensors with ion homeostasis and transport-mediated processes are largely unknown. Current research indicates that plant *Integrin-Linked Kinases* (*ILK*s), a subfamily *Raf-like MAP2K Kinases*, may have evolved to fulfill this role. In this review, we explore new findings on plant *ILKs* placing a particular focus on the connection between ILKs proteins unique structural features and ILKs functions. The ankyrin repeat motifs and the kinase domains of ILKs in *Arabidopsis* and land plants lineage, respectively, are analyzed and discussed as potential determinants of ILKs’ metal ion cofactor specificity and their enzymatic and interaction activities. Further, *ILKs* regulation through gene expression, subcellular localization, and ions and ion transporters is reviewed in the context of recent studies. Finally, using evidence from literature and interactomics databanks, we infer ILKs-dependent cellular pathways and highlight their potential in transmitting multiple types of signals originating at the interface between the cell wall and plasma membrane.

## Introduction

Protein kinases constitute one of the largest and most diverse enzyme families in living systems. From single-cell and complex organisms, kinases facilitate detection of the organism’s immediate environment and generate local and systemic responses that govern growth and survival. Among complex eukaryotes, plants support a larger and more structurally complex set of kinases compared to animals. The expansion of the plant kinases is thought to be the result of the selective pressure of the environment and possibly contributed to the evolution of plants from aquatic to land-based niches (Hanada et al., 2008). In particular, a group of MAP3Ks – the Raf-like kinase (RAF) family – underwent a tremendous increase in the number and structural variety of its members compared to metazoans (Champion et al., 2004). The heterogeneity of plant RAFs compounded by the lack of information on the signaling, mechanistic and regulatory aspects for the majority of RAFs, makes the task of associating them to specific pathways and biological processes, difficult.

A subfamily of RAFs having unique characteristics include the Integrin-Linked Kinases (ILKs). ILKs were first described in metazoans as interactors of integrin transmembrane receptors and having key roles in assembling signaling complexes to membrane focal adhesion sites (Hannigan et al., 1996). Integrins coordinate signaling both intra- and extracellular processes including cell polarity, the cytoskeleton, cell adhesion, migration, and assembly of the extracellular matrix (ECM) (Sakai et al., 2003; Elad et al., 2013). While the roles of the metazoan ILK in some of these processes has been recognized, the study of this family in plants has just begun.

Plants, unlike metazoans, maintain multiple ILK genes in their genomes. Plant *ILKs* were first described in *Medicago* and *Arabidopsis* as ankyrin kinases proposed to act in nodulation and secondary root growth (Chinchilla et al., 2003, 2008). Recently, *ILK1* was demonstrated to regulate plants’ sensitivity to osmotic and salt stress, response to nutrient availability, and susceptibility to bacterial pathogens (Brauer et al., 2016). Most of the roughly 50 plant RAFs have unknown functions and poorly understood signaling mechanisms.

Two fairly well-studied RAFs – EDR1 and CTR1 – must be noted as an exception. EDR1 operate as a suppressor of disease resistance and programmed cell death (PCD) by modulating signal processing through three hormone-mediated pathways: salicylic acid, ethylene, and abscisic acid (Frye and Innes, 1998; Tang et al., 2005). At the molecular level, EDR1 was shown to suppress the accumulation of MAP2Ks (MKK4 and MKK5) and MAPKs (MPK3 and MPK6), possibly through direct interactions with the proteasome degradation machinery (Zhao et al., 2014). Indeed, the activities of the RING-finger E3 ligases KEG and ATL1 were regulated by EDR1 through direct interactions (Wawrzynska et al., 2008; Serrano et al., 2014). CTR1 was identified as a suppressor of the ethylene response pathway and a modulator of stress-induced changes in plant growth (Kieber et al., 1993; Zhao et al., 2014). Subsequent studies showed that in the absence of ethylene, the ETR receptors activate CTR1, which in turn phosphorylates and thus, inhibits the relocalization in the nucleus of EIN2, the central regulator of the ethylene-activated transcriptional program (Huang et al., 2003; Ju et al., 2012). The CTR1-controlled ethylene pathway has been recently linked to drought tolerance (Shi et al., 2015). Similar to EDR1, CTR1 modulates ABA signaling; however, this particular aspect of its functions is preserved in bryophytes but absent in the land plants (Yasumura et al., 2015).

These findings, alongside RAF-centered literature, underscore two common themes within this diverse family: (i) RAFs are described as participants in cellular pathways activated by extracellular signals, including both biotic and abiotic factors (Tang et al., 2005; Hashimoto et al., 2006; Saruhashi et al., 2015); (ii) The presence of regulatory as well as catalytic domains in RAFs structure indicates a possible general role of ‘adaptors,’ elements which link membrane sensors and diverse intracellular signaling complexes through stable physical interactions or phosphorylation activity. Here, we focus on plant *ILKs*, which are compared with their metazoan counterparts in their structural features, regulation, interaction partners, and signaling activity. We analyzed *ILK1*-like sequences in representative species of land plants, highlighting ILK specific features in the ankyrin repeat (AR) and kinase domains (KDs), and summarized recent literature on the regulation of *ILKs* at multiple levels, and identified known and potential components of signal processing pathways mediated by ILKs. We propose a model of stress-triggered signal transduction that emphasizes the contributions of *ILKs* and emphasizes future exploration topics.

## Structural Features of ILKSs

**FIGURE 1:**
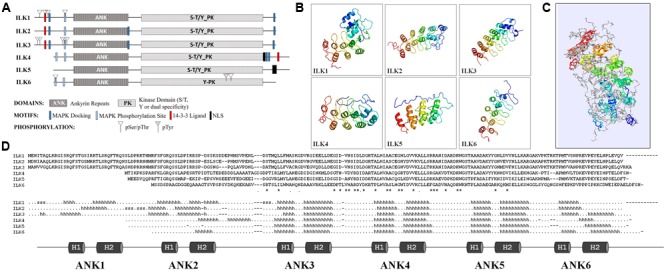
**Structural features of the *Arabidopsis* ILKs. (A)** Diagrams of the full-length primary protein sequences of the *Arabidopsis* ILKs containing motifs predicted using the ELM database of eukaryotic linear motifs. **(B)** Predicted 3D structures of ILKs ankyrin repeats using PHYRE2. Images are colored by rainbow N → C-terminus. **(C)** Superimposed ILKs’ 3D structures. The structures are colored to represent the succession of secondary structure elements (N-terminal/blue to C-terminal/red). The structural superposition of the PHYRE2 predicted 3D structures and alignments were performed using the ‘Iterative fit’ function of the Swiss-PDB Viewer 4.1.0. (Guex and Peitsch, 1997). **(D)** The alignment displays the amino acid sequence in each ILK. ‘^∗^/.’ indicate identical/similar residues. The predicted secondary structure is displayed below the alignment (*h*: α-helices, *s*: strands). The ankyrin repeats (ARs) are shown below and highlighted on the secondary structure alignment.

The unifying characteristics of plant and animal ILKs are the N-terminal ARs and the C-terminal eukaryotic KD. An initial predictive structural analysis (**Figure 1A**) using Eukaryotic Linear Motif (ELM) resource (Dinkel et al., 2016) shows that ILKs have a bi-globular 3D structure in which the ARs fold forms the first globular unit and the KD fold creates the second (**Supplementary Data 1**). Several predicted motifs in ILK might link them with MAP kinase (MAPK) cascades. As such, MAPK docking motifs (D-motifs) and Pro-directed kinase phosphorylation sites exists in most ILKs N- and C-termini; also, 14-3-3 ligand sites were found in ILKs 1 to 4. Experimentally identified phosphoresidues in ILKs reside mostly within the N-terminal region in the vicinity of the MAPK docking, Pro-directed phosphorylation, and 14-3-3 ligand motifs, as shown in PhosPhat 4.0 database (Zulawski et al., 2013). In the six *Arabidopsis* ILKs, phosphorylated residues consist of pSer or pThr, barring one internal pTyr in ILK6. Based on homology, the *Arabidopsis* ILKs are predicted by TAIR^1^ to function as Ser/Thr, or dual Ser/Thr and Tyr kinases, except ILK6 which may act as a Tyr-specific kinase.

### ILKs Ankyrin Repeats

The higher divergence among *Arabidopsis* ILKs in the N-terminal region prompted an analysis of the ILKs ARs. The structures of the AR motifs of the *Arabidopsis* ILKs were modeled using PHYRE2 Intensive mode (Kelley et al., 2015), and 3D structures were derived with high confidence by homology to ankyrin-containing structures (**Figure 1B** and **Supplementary Data 2**). ARs are composed of modules of two antiparallel α-helices followed by a β-hairpin or a long loop (helix-loop-helix-β hairpin/loop fold); consecutive repeats stack together to form a curved L-shaped domain. In all ILKs, the three ARs located in the middle/C-terminal end of the AR region, consisting of 6-residue outer helices, 4-residue inner helices, and long inter-repeat loops of 16 residues in average, are conserved. Despite a relatively weak sequence conservation between ILKs 1–3 or ILKs 4–6 (42% identity in average), the organization of ARs is remarkably conserved, as also shown by the superposition of all ILK models (**Figure 1C** and **Supplementary Data 3**). Additional ARs may exist in ILKs 1–4; partially formed ARs are located at the extremities of the AR regions in most ILKs (**Figure 1D**). In general, the significance of the number of ARs for the protein function is not entirely understood; nevertheless, structural and kinetic studies have addressed the importance of the number of repeats in folding, stability, and the molecular interactions established by AR proteins (Wetzel et al., 2008). It is expected that in ILKs as well, the ARs determine the specificity and affinity of ILKs for interacting partners.

### ILKs Kinase Domains

The plant and animal ILK-KDs have diverged considerably. The eukaryotic KD is composed of 12 subdomains with conserved amino acid residues essential for binding of the ATP complexed to the divalent cation cofactor, the phosphotransfer reaction, and regulation of kinase activity through phosphorylation of internal residues. Animal ILKs contain abundant substitutions in conserved residues of the KD and, although their kinase activity may be possible, extensive analyses substantiate the view that they are true pseudokinases (Wickström et al., 2010). By comparison, plant ILKs contain fewer substitutions and some, at least, can catalyze a phosphotransfer reaction, in both autophosphorylation and substrate phosphorylation assays (Chinchilla et al., 2008; Brauer et al., 2016).

**FIGURE 2:**
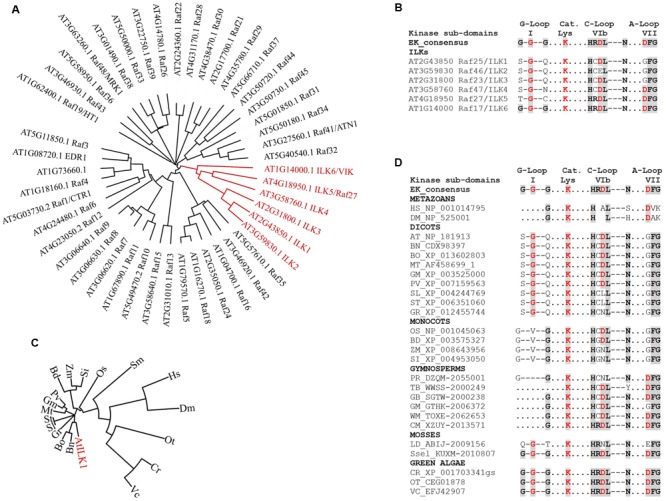
**The evolution of ILKs kinase domains in plants. (A)** Phylogenetic tree of *Arabidopsis* RAF kinases; the ILK clade is shown in red. **(B)** Alignment of kinase subdomains of the *Arabidopsis* ILKs showing conservation of residues in the G-loop, catalytic Lys, C-loop and the A-loop. In both B and D, the eukaryotic consensus of each kinase subdomain is shown for comparison. Conserved residues are shaded. **(C)** Phylogenetic tree of plant ILK1-like kinases; the AtILK is shown in red. ILK1 orthologs were identified in the NCBI database using BLAST; accessions with the highest score from each plant were selected. **(D)** Alignment of kinase subdomains shown in C. For C and D, full binominal names are as follows: HS (*Homo sapiens*), DM (*Drosophila melanogaster*), AT (*Arabidopsis thaliana*), BN (*Brassica napus*), BO (*B. oleracea var. oleracea*), MT (*Medicago truncatula*), GM (*Glycine max*), PV (*Phaseolus vulgaris*), SL (*Solanum lycopersicum*), ST (*S. tuberosum*), GR (*Gossypium raimondii*), OS (*Oryza sativa Japonica Group*), BD (*Brachypodium distachyon*), ZM (*Zea mays*), SI (*Setaria indica*), PD (*Pinus radiata*), TB (*Taxus baccata*), GB (*Ginkgo biloba*), WM (*Welwitschia mirabilis*), CM (*Cycas micholitzii*), LD (*Lycopodium deuterodensum*), Sse (*Selaginella selaginoides*), CR (*Chlamydomonas reinhardtii*), OT (*Ostreococcus tauri*), VC (*Volvox carteri f. nagariensis*). The alignments were performed using Clustal Omega; the phylogenetic trees were constructed and visualized using the iTOL Interactive Tree of Life. Protein sequences were obtained from the NCBI (http://www.ncbi.nlm.nih.gov/) or the 1000 Plants (OneKP). Red residues in **(B)** and **(D)** are known to be important for catalytic activity in eukaryotic kinases.

Scarce information exists regarding plant RAFs kinase activity and phosphorylation substrates. The animal Rafs are *bona fide* MAP3Ks – that is, they function within MAPK cascades. Except for CTR1, the majority of plant RAFs may not act as canonical MAP3Ks. ILKs bring additional complexity by possessing unique structural features that set them apart from the rest of the RAFs (**Figure 2A** and **Supplementary Figure S1**). ILK1 and its closest homologs, the pollen-specific ILK2-3 exhibit three specific KD alterations (**Figure 2B**):

(i) The Gly-loop (sub-domain I), which associates with the phosphate group of the bound ATP, consists of only one conserved Gly residue. In the absence of compensating substitutions, a malformed G-loop may interfere with the ATP-binding (Zeqiraj and van Aalten, 2010), although catalytically active kinase may only contain a partially conserved motif.
(ii) The HRDXXXN motif, which is located in the catalytic loop (C-loop, sub-domain VIb) and correctly orients the -OH in the substrate residue to be phosphorylated, contains non-conserved substitutions for Arg, and both Arg and Asp in ILK2. Asp is the proton-accepting base for catalysis, and thus essential for catalytic activity. Mutations of Arg have not been so far correlated to kinase inactivation in plants or other eukaryotes (see the review on non-RD kinases, Dardick et al., 2012).
(iii) The Asp in the DFG motif, which is located in the activation loop (A-loop, sub-domain VII) and binds the divalent cation complexed to ATP in conjunction with the Asn in HRDXXXN motif, is mutated to GFG.

Interestingly, the more distantly related ILK4 (Raf47), ILK5 (Raf27), and ILK6 (VIK, Raf17) contain canonical A-loops and/or C-loops; ILK6 also has a fully formed G-loop (**Figures 2A,B**). It is possible that alterations in the canonical kinase motifs may lead to an atypical reliance on Mn^2+^ as a cofactor in plant ILKs; however, the residues/motifs that regulate Mn^2+^ binding, and the consequence of Mn^2+^ utilization for ILKs activity are currently unknown.

The prevalence of ILK-KD modifications within the green plant lineage was examined by analyzing *ILK1*-like sequences in evolutionarily recent and ancient species of Viridiplantae (**Figures 2C,D**). Orthologs in green algae and mosses exhibit canonical residues at most positions in the kinase motifs, suggesting that they may represent an ancient ILK with biochemical properties of the eukaryotic kinases. Remarkably, the malformed G-loop and substitutions in the C- and A-loops first appear together in Angiosperms. Most dicots and monocots orthologs analyzed contained incomplete G-loops and single-amino-acid substitutions in the C- and A-loops. Notably, in several orthologs including Solanaceae (potato and tomato), and monocots (maize and *Setaria indica*) the Asp in HRDL was also mutated, suggestive of kinases with residual or absent activity (Shi et al., 2010). These findings demonstrate the presence of a range of active kinases with conserved or atypical features alongside potential pseudokinases and raise the question of what was the evolutionary pressure to maintain this variability. Although a clear answer is not available yet, these observations provide evidence of plant ILKs with catalysis-dependent and -independent functions.

## Regulation of *ILKs*

Regulation of *ILKs* is achieved by both transcriptional and post-translational mechanisms. Animal ILKs are regulated by phosphorylation and interaction with protein partners who influence inter-compartmental shuttling such as the cytosol-nucleus migration (Acconcia et al., 2007). Plant *ILKs* appear to be under a complex regulation at the level of gene expression, protein activity, and interactions, and we discuss below several regulatory means that highlight both unique and shared aspects of plant and metazoan *ILKs*.

### Expression and Localization

Published data on the localization of plant ILKs is sparse. However, information on the tissue-specific expression patterns of *ILK* genes may provide clues on the possible localization of the proteins. According to global transcriptomics studies in *Arabidopsis*^2^, *ILK* transcripts are expressed at high to medium levels in multiple plant tissues including leaf, root, and vasculature. *ILK1* transcripts were identified in mesophyll, lateral root, xylem, and root cortex cells, while both *ILK2* and *ILK3* have the highest expression in pollen. *ILK2* is also present in root epidermis, cambium, and elongation zone cells, while *ILK3* is also expressed in guard cells and root cortex. *ILK4* has an abundant expression in the endosperm, guard cells, and root tissues; *ILK5* and *ILK6* are highly expressed in leaves, guard cells, and in root tissues such as the cap, epidermis, and xylem.

Information on the subcellular localization of ILKs was generated primarily by mass spectrometry discovery proteomics. Notably, despite lacking transmembrane regions, several ILKs were found associated with the PM or intracellular membranous organelles. ILK1 co-localized with the PM and ER markers (Brauer et al., 2016), and ILK4 and ILK6 were identified in the PM, Golgi and tonoplast (Benschop et al., 2007; Whiteman et al., 2008; Elmore et al., 2012; Heard et al., 2015). ILK4 and ILK5 may be nuclear-localized as predicted by the bipartite or monopartite nuclear localization signals within their C-termini (**Figure 1**); an ILK5-GFP fusion was detected both in the cytosol and nucleus (Koroleva et al., 2005; Ito et al., 2011).

### Enzymatic Activity

The AtILK1 and MsILK are dual Mn^2+^/Mg^2+^ kinases which demonstrate the highest activity with Mn^2+^ as a cofactor; the metazoan ILK may show a similar cofactor-binding specificity (Maydan et al., 2010), although its kinase activity is still controversial (Fukuda et al., 2011; Reiterer et al., 2014). The divalent cations Mg^2+^ and Ca^2+^ are the cofactors of choice for the majority of eukaryotic kinases, affecting kinase conformation and orientation of ATP in the active site ^3^. A literature search revealed multiple plant kinases with dual or Mn^2+^ specific activities. Several receptor-like kinases (RLKs) and other PM-associated kinases were shown to have Mn^2+^-dependent activity. An RLK from *Solanum chacoense*, ScORK28, has dual Mn^2+^/Mg^2+^ activity; Mn^2+^ stimulated its activity at levels above Mg^2+^ (Germain et al., 2007). The AtCRK6 and AtCRK7 Cys-rich RLKs mediate signaling triggered by extracellular reactive oxygen species during the immune response; despite high conservation between the two AtCRKs and their canonical HRDL/DFG motifs, AtCRK6 activity was highest with Mn^2+^ rather than Mg^2+^ while AtCRK7 was Mg^2+^-dependent (Idänheimo et al., 2014). RPG1, an RLK that confers resistance to the rust fungus *Puccinia graminis* in barley, and ETR1 ethylene receptor and potential determinant of disease susceptibility to *Fusarium oxysporum*, exhibit higher activity with Mn^2+^ (Nirmala et al., 2006; Voet-van-Vormizeele and Groth, 2008). Likewise, an analysis of the kinase activity the phytosulfokine receptor PSKR1, which promotes cell elongation and modulates responses to bacterial and fungal pathogens, has Mn^2+^-dependency (Hartmann et al., 2015). AtCaMRLK, a CaM-binding RLK, displayed a preference for Mn^2+^ over Mg^2+^, as showed by its higher kinase activity on a generic substrate (Charpenteau et al., 2004). Several cytosolic Ca^2+^-dependent kinases from the CIPK family displayed enhanced activity with Mn^2+^ (Shi et al., 1999). The contribution of Mn^2+^ as a kinase cofactor in plants is far from marginal, and further studies should address the prevalence and functional relevance of this property among plant kinases associated with pathogen defense.

### Regulation by Ca^2+^ Sensors

Connections have been recently made in metazoans between integrin signaling and Ca^2+^-mediated pathways. The absence of *ILK* in epidermal cells led to defective cell differentiation and aberrant translocation of the CaSR calcium-sensing receptor (Sayedyahossein et al., 2016). In plants, CML9, a Ca^2+^ sensor, and negative PTI modulator interacted with ILK1 *in vivo* and inhibited ILK1 kinase activity (Brauer et al., 2016), suggesting that ILK1-CML9 are part of a putative pathway mediated by pathogen-triggered Ca^2+^ waves. Although limited, these findings support the existence of reciprocal regulatory relationships between ILK signaling and Ca^2+^, which may thus influence multiple cellular functions.

## Plant Cell Signaling Pathways Mediated By ILKs

### Plant Integrin-Like Receptors

Integrins are heterodimers composed of α- and β-subunits each with a large ectodomain, a transmembrane domain, and a short cytoplasmic region. Integrins are activated by binding via ectodomain to the Arg-Gly-Asp (RGD) motif present in the proteins of the ECM (e.g., fibronectin, collagen). Integrins are also activated by cytoplasmic signals (e.g., talin, actinin) through interactor-binding to the cytosolic region of the β-subunit. As integrins lack enzymatic activity, signaling is triggered by the assembly of complexes on the cytoplasmic face of the membrane following receptor clustering and conformational changes (Campbell and Humphries, 2011).

*Bona fide* integrin receptors are absent in plants. Nevertheless, plant integrin-like receptors (ILRs) have been proposed based on functional or structural analogy to the animal integrins. As such, structural models predicted for the PM-associated protein NDR1 showed that the protein possesses structural homology to the integrin’s β subunit (Century et al., 1997). Notably, the postulated function of NDR1 in the adhesion between the cell wall (CW) and PM, is reminiscent of integrin’s roles in coupling the cell’s ECM and membrane. Furthermore, the altered pathogen-associated molecular pattern-triggered immune (PTI) response and defective electrolyte leakage exhibited by *ndr1-1* mutants, parallel the PTI and ion homeostasis phenotypes of *ilk1-1* (Brauer et al., 2016) and suggest that NDR1 and ILK1 are components of the same cellular pathways. Another equally compelling contender for a plant ILR is the lectin receptor LecRK-I.9, demonstrated to bind the *bona fide* integrin ligand – the tripeptide RGD – present in the oomycete *Phytophthora infestans* effector IPI-O (Gouget et al., 2006). *LecRK-I.9* contributed to disease resistance toward *Phytophthora* and was proposed to maintain robust CW–PM interactions during infection (Bouwmeester et al., 2011).

Extending the ECM-membrane/CW-membrane analogy, a whole suite of potential plant ILRs emerges in the form of PM-associated receptor-like proteins (RLPs) that may provide a physical CW–PM link. Some RLPs were shown to modulate the developmental and stress-induced responses at the CW–PM continuum indicating that effective communication is occurring between these cellular components. The CW-sensing receptors from the CrRLKs family (e.g., THE1 and FER) control cell growth and expansion, pollen tube elongation, and pathogen resistance, probably through sensing physiological or pathogen-induced changes in the chemical composition of CW (Wolf et al., 2012). Thus, plant-specific ILK functions downstream potential ILRs may include the response to multiple types of signals that alter PM and CW integrity.

### Ion Transporters and ILKs

K^+^ transport emerges as a possible common denominator of *ILKs* signaling in plant and animal kingdoms. In plants, the function on *ILK1* in PTI was shown to be coupled to cellular K^+^ fluxes (Brauer et al., 2016). In animals, K^+^ efflux induced by the intracellular recognition of PAMPs including flagellin is currently considered a standard signal for the assembly of receptor complexes and the activation of defense (He et al., 2016). The extent to which changes in intracellular K^+^ gradients constitute signals or mediate immune signal transduction pathways in plants is not entirely understood. Global transcriptional responses to K^+^ starvation and re-supply revealed possible components and pathways for perception and integration of changes in K^+^ concentration. Among them, calmodulins emerged as prominent responsive genes, including CML9 (Armengaud et al., 2004); notably, CML9 is an ILK1 interactor and potential activity regulator (Brauer et al., 2016).

An important insight into how K^+^-mediated cell growth and elongation may play into plant *ILK1*-modulated pathways was recently provided by a study of signaling for cell growth focused on CNGC17, a Ca^2+^/CaM-regulated and a possible K^+^/Ca^2+^-influx channel (Ladwig et al., 2015). The study postulates that cell expansion is mediated by a cation influx through CNGC17 and H^+^-ATPases activated by the PSKR1/BAK1 complex having dual growth/immune functions.

**FIGURE 3:**
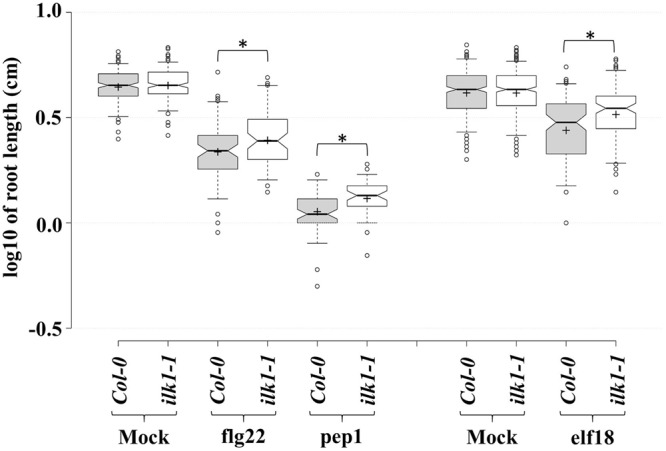
**PAMP and DAMP perception is mediated by *ILK1* signaling.** For flg22 and pep1 treatments, *Arabidopsis* Col-0 and *ilk1-1* seedlings were germinated on MS basal salt mixture supplemented with 2.5 mM MES, 0.9% agar, and 1% sucrose, at 22°C with a long-day (16 h light/8 h dark) photoperiod. For elf18 treatment, seeds were germinated on MS basal salt mixture supplemented with 0.6% agar, 3% sucrose, at 22°C under short-day (8 h light/16 h dark) photoperiod. Five-day (for flg22 and pep1) or seven-day-old (for elf18) seedlings were transferred to liquid MS ±1 μM peptide. The effect of treatment on root growth was analyzed at 10 days post-treatment and visualized in the box plots. Center lines show the medians, box limits indicate the 25th and 75th percentiles, whiskers extend to 5th and 95th percentiles, outliers are represented by dots, and crosses represent sample means. From left to right, *n* = 183, 197, 69, 64, 61, 46, 319, 331, 106, 105 sample points. ‘^∗^’ symbols represent statistical significance (*p* ≤ 0.01).

Moreover, an analysis of *ilk1-1* sensitivity to root growth suppression by other peptide signals revealed the reduced sensitivity of the mutant to elf18 and pep1 (**Figure 3**) indicating that *ILK1* in addition to its contribution to FLS2-mediated signaling, may also participate in EFR and PEPR1/2 pathways. Thus, a role for ILKs as possible signal integrators or scaffolds can be envisioned in PM-bound clusters of transporter and receptor complexes generating downstream inputs into immune and growth signaling pathways.

### ILK-Mediated Cellular Pathways

**FIGURE 4:**
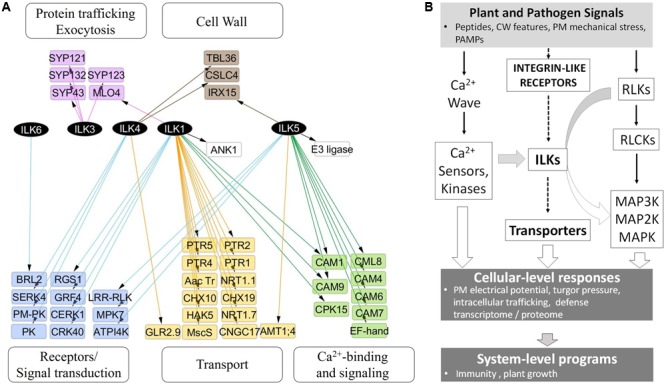
**ILKs integrate Ca^2+^- and receptor-mediated signaling in the plant immune response. (A)** Nodes represent proteins and edges represent experimentally verified interactions (solid line). The arrangement of nodes is based on the classical a tiered organization of signal transduction cascades; postulated plant integrin-like receptors and plasma membrane receptors are placed in the upper tier, followed by transporters, ILKs, Ca^2+^-regulated proteins, and phospho-site interacting 14-3-3 proteins. The network was constructed using the information in the Biogrid database and visualized using Cytoscape 3.2.1. **(B)** Proposed model of ILKs’ roles in signal transduction; the arrows show the known (continuous lines) or postulated (broken lines) direction of signal transduction. Plant or pathogen-derived signals reach the plant cell and activate several signal-processing pathways. (1) Plant Integrin-like receptors sense shifts in the composition and mechanical tension of the cell wall and plasma membrane; ILKs are possible transducers of signals from these receptors, through modulation of transporter activity. (2) Membrane receptor-like kinases (RLKs) bind specific ligands to recruit and activate cytosolic proteins (RLCKs); downstream MAP kinase module transmits the signal to effector proteins located in diverse cellular compartments. (3) Rapid calcium fluxes triggered by stimuli, increase Ca^2+^ concentration in the cytosol and activate Ca^2+^ sensors. ILKs bridge ion- and receptor-mediated signaling pathways. ILKs participate in pathways activated by PAMP and DAMP receptors, and their activity may be modulated by Ca^2+^ sensors (such as calmodulins). Concerted activation of these pathways results in immediate physiological changes, including the depolarization of the PM, turgor pressure, and induction of the transcriptome program. The plant response to pathogen attack encompasses both the activation of the immune system (defense program), as well as appropriate modifications in the plant growth. Continuous lines represent events for which experimental evidence exists, while broken lines depict proposed pathways.

In stark contrast to animal ILKs, for which a search of the BioGrid^4^ revealed over 200 known and potential functional interactors, knowledge of plant ILKs associations with protein partners and their signaling roles is much more limited. We inferred possible signaling pathways and physiological processes modulated by plant ILKs by generating an interaction network using information from several repositories (BioGrid, MIND, and BAR) (**Figure 4A** and **Supplementary Table S1**). In it, ILK1 and ILK5 establish the largest number of interactions, followed by ILK4, ILK3, and ILK6. With two exceptions, all interactors belong to several functional categories – transport, Ca^2+^-sensing and signaling, receptor and signal transduction, intracellular trafficking, and cell wall. A majority of ILK1 interactors are transporters, Ca^2+^ sensors, and kinases; ILK5 mostly interacts with Ca^2+^ sensors and kinases; ILK4 interacts with receptors and intracellular kinases, and ILK3 interacts exclusively with exocytosis-related proteins. When pondering on the large and diverse number of transporters interacting with ILK1, the inevitable question arises on whether *ILK1* loss-of-function (LOF) could lead to a collapse in cellular transport. All *ILK1* mutants generated to date are partial LOF (Brauer et al., 2016); potential partial and complete LOF T-DNA insertion lines for ILK4 and 5, which share tissue expression similarities with ILK1, are under analysis in our lab and it remains to be seen whether higher-order mutants will exhibit major biological defects. Nevertheless, the propensity of ILKs and other RAFs to associate with and control/be controlled by transporters remains to be demonstrated. Other than the ILK1-HAK5, one more example of a validated functional interaction between RAF and a transporter may exist, namely the CTR1-EIN2, where EIN2 shows similarity to the Nramp Mn^2+^/Fe^2+^-transporters (Ju et al., 2012).

The information from the interaction network was integrated into a model of signal transduction comprising proposed ILK-mediated signaling pathways and downstream cellular processes and phenotypes (**Figure 4B**). In the model, endogenous plant signals originating from the cell wall alongside pathogen-derived signals, such as pathogen- or damage-associated molecular patterns (PAMPs/DAMPs), activate complexes in the plasma membrane (PM) that include RLPs, ILRs, and ion channels. Several parallel signal transduction pathways are activated downstream these complexes and include MAPK cascades downstream PAMP-activated RLKs, ion transporters through the ILR-ILK axis, and Ca^2+^-dependent signaling. Outcomes include induction of the defense transcriptome (immune response), changes in the membrane electric potential, protein trafficking and ion transport, which impact plant defense and growth responses.

## Conclusion and Future Perspectives

Plant ILKs have the hallmarks of multi-valent kinases with a modular domain organization that drives domain-specific interactions. Our analysis reveals potentially new plant-specific ILK functions and places them as components in multiple signal transduction pathways, acting through a wide range of mechanisms to regulate cellular homeostasis. Biotechnology applications that are exploiting knowledge of ILKs, and other RAFs, can be envisioned to address pathogen resistance and stress tolerance in crops. However, several difficulties must be overcome to reach that point.

Firstly, our knowledge of their cellular partners and mechanisms of actions is still rudimentary. We have little insight into the molecular details of how distinct structural features of ILKs contribute to their cellular roles as kinases and scaffold proteins. Signaling triggered by ILRs and ILKs appears to coordinate signaling in response to multiple plants- and pathogen-derived signals, as well as abiotic stresses that alter the CW integrity, or the plant cell turgor pressure. Is it possible to link distinct ILKs structural features to ILKs inputs in pathogen- or stress-triggered pathways? In other words, can we separate the defense and abiotic stress functions of ILKs by engineering specific domains or motifs in their structure? Future research endeavors should center on the duality of ILKs as potential signal integrators and scaffolding components in multiple signaling pathways. It would be informative to uncover the ILRs, ILKs substrates and interacting partners, alongside ILKs contributions to pathogen- and stress-induced changes in the transcriptome and phosphoproteome.

Secondly, the ILK-mediated mechanisms that link the PM electrical potential to signal processing are poorly characterized. Membrane potential maintained through the activity of channels and transporters is known as an important source of energy and modulator of signal transduction in animals through the nanoscale re-organization of the PM and assembly of signaling complexes (Zhou et al., 2015), and a common denominator of the pathogen and abiotic stress responses. We suggest that modules of ILKs, transporters and membrane receptors may provide a mean for the activation of signal transduction and its modulation in rapport to the electrical state of the PM and ion homeostasis. Ions are components of biochemical reactions and essential micronutrients; however, their potential in acting as regulatory elements in pathogen defense is a virtually unknown area. The study of ILKs may provide a framework for defining this relationship in plants.

Finally, considering the ubiquity of ILKs in plants, it is likely that the components of ILK-mediated pathways vary with the tissue and cellular environment and are determinants of signaling specificity. It would be informative to isolate leaf, root, or pollen-specific ILK complexes and place them in the known signaling networks. Moreover, high-throughput interactomics and phosphoproteomics studies would give the much-needed support in mapping RAF-mediated intracellular pathways and interactomes. Such studies would open the possibility of directing plant signaling toward beneficial phenotypic outcomes.

## Author Contributions

SP and GP performed analyses; GD performed experiments, SP, GP, and EB wrote the manuscript.

## Supplementary Material

The Supplementary Material for this article can be found online at: http://journal.frontiersin.org/article/10.3389/fpls.2017.00376/full#supplementary-material

FIGURE S1 **Alignment of all RAF sequences from *Arabidopsis thaliana* using Clustal Omega.** The three conserved kinase motifs that are changed in ILKs – the Gly loop, the HRDLxxxN motif in the subdomain VIb, and the DFG motif in the start of the activation domain (A-loop) – are highlighted.

TABLE S1 **Interaction partners of the *Arabidopsis* ILKs**.

DATA S1 **Model and information associated with 3D structure prediction of full length ILK1**.

DATA S2 **Model and information associated with 3D structure prediction of ankyrin repeat domains of ILK1 to ILK6**.

DATA S3 **Information associated with superposition of the ankyrin repeat domains of ILK1 to ILK6**.

## Conflict of Interest Statement

The authors declare that the research was conducted in the absence of any commercial or financial relationships that could be construed as a potential conflict of interest.

## Footnotes

1 www.arabidopsis.org

2 https://genevisible.com/

3 europepmc.org/articles/pmc4318763

4 http://thebiogrid.org/
