# Supplementary material for: Insights into the Structure, Function, and Ion-Mediated Signaling Pathways Transduced by Plant Integrin-Linked Kinases
Source: Front Plant Sci. 2017 Apr 3;8:376. doi: 10.3389/fpls.2017.00376 (PMC5376563; doi:10.3389/fpls.2017.00376)
Supplement: FIGURE S1 — Alignment of all RAF sequences from Arabidopsis thaliana using Clustal Omega. The three conserved kinase motifs that are changed in ILKs – the Gly loop, the HRDLxxxN motif in the subdomain VIb, and the DFG motif in the start of the activation domain (A-loop) – are highlighted. [file Image_1.pdf]

CLUSTAL O(1.2.1) multiple sequence alignment

```
AT1G04700.1_Raf16           ----------------------------MRMEFPGSSNQHLGRDRF--N-GE--VGCGNN
AT2G35050.1_Raf24           MDQAKGYEHVRYTAPDPRDEGLGSINQRFSHDSSTNVNTYVRPPDYGVSTPA--RPVLNY
AT1G16270.1_Raf18           MDRNRPPHPFQQHAMEPG-YVNDSVPQGFTPDQTGLSNANVRPN----PADV--KPGLHY
AT1G79570.1_Raf5            MDKARHQ-QLFQHSMEPG-YRNETVPQPFMPDQTGSASANMRPPNS-NGSDV--KAVHNF
AT3G46920.1_Raf42           ------------------------------------------------------------
AT5G57610.1_Raf35           ------------------------------------------------------------
AT1G08720.1_EDR1            ------------------------------------------------------------
AT5G11850.1_Raf3            ------------------------------------------------------------
AT1G18160.1_Raf4            ------------------------------------------------------------
AT1G73660.1                 ------------------------------------------------MKV-----KEET
AT4G24480.1_Raf6            ---------------MPHR-TTYFFPRQFPDRG------------FDSFSLKNDHDKKKS
AT5G03730.2_Raf1/CTR1       -------------MEMPGRRSNYTLLSQFSDDQVSVSVTGAPPPHYDSLSSENRSNH--N
AT4G23050.2_Raf12           ------------------------------------------------------------
AT5G49470.2_Raf10           ------------------------------------------------------------
AT1G67890.1_Raf11           ------------------------------------------------------------
AT3G06620.1_Raf7            ------------------------------------------------------------
AT3G06630.1_Raf8            ------------------------------------------------------------
AT3G06640.1_Raf9            ------------------------------------------------------------
AT1G14000.1_VIK             ------------------------------------------------------------
AT2G31800.1_ILK3            ------------------------------------------------------------
AT2G43850.1_ILK1            ------------------------------------------------------------
AT3G59830.1_ILK2            ------------------------------------------------------------
AT3G58760.1_ILK4            ------------------------------------------------------------
AT4G18950.1_Raf27           ------------------------------------------------------------
AT2G31010.1_Raf13           ------------------------------------------------------------
AT3G58640.1_Raf15           ------------------------------------------------------------
AT5G01850.1_Raf31           ------------------------------------------------------------
AT5G50180.1_Raf34           ------------------------------------------------------------
AT3G27560.1                 ------------------------------------------------------------
AT5G40540.1_Raf32           ------------------------------------------------------------
AT5G66710.1_Raf37           ------------------------------------------------------------
AT3G50720.1_Raf44           ------------------------------------------------------------
AT3G50730.1_Raf45           ------------------------------------------------------------
AT3G63260.1_Raf48/MRK1      ------------------------------------------------------------
AT3G22750.1_Raf39           ------------------------------------------------------------
AT4G14780.1_Raf26           ------------------------------------------------------------
AT3G01490.1_Raf38           ------------------------------------------------------------
AT5G50000.1_Raf33           ------------------------------------------------------------
AT4G38470.1_Raf30           ------------------------------------------------------------
AT2G17700.1_Raf21           ------------------------------------------------------------
AT4G35780.1_Raf29           ------------------------------------------------------------
AT2G24360.1_Raf22           ------------------------------------------------------------
AT4G31170.1_Raf28           ------------------------------------------------------------
AT1G62400.1_Raf19/HT1       ------------------------------------------------------------
AT3G46930.1_Raf43           ------------------------------------------------------------
AT5G58950.1_Raf36           ------------------------------------------------------------


AT1G04700.1_Raf16           CSQTGEEFSNEFLRDFGAQRRLQHGGVNRNVEGNYNNRHLVYEDFNRILGLQRVDSNMSE
AT2G35050.1_Raf24           SIQTGEEFAFEFMRDRVIMKPQFI----PNVYGEHSG-MPVSVNL-SALGMVHPMSESGP
AT1G16270.1_Raf18           SIQTGEEFSLEFLRDRVISQRSAN----PIAAGDINY-PTGYNG--------HAGSEFGS
AT1G79570.1_Raf5            SIQTGEEFSLEFMRDRVIPQRSSN----PNGAGDMNY-NTGYMELRGLIGISHTGSECAS
AT3G46920.1_Raf42           --------------------------------------------------MAHEPSSPSS
AT5G57610.1_Raf35           ------------------------------------------------------------
AT1G08720.1_EDR1            ------------------------------------------------------------
AT5G11850.1_Raf3            ------------------------------------------------------------
AT1G18160.1_Raf4            ------------------------------------------------------------
AT1G73660.1                 LKNLGDG-----------------------------------------------------
AT4G24480.1_Raf6            SSNVGESFGFQ--RDN--------------------------------------------
AT5G03730.2_Raf1/CTR1       SGNTG-------------------------------------------------------
AT4G23050.2_Raf12           ------------------------------------------------------------
AT5G49470.2_Raf10           ------------------------------------------------------------
AT1G67890.1_Raf11           ------------------------------------------------------------
```

AT3G06620.1_Raf7	--
AT3G06630.1_Raf8	--
AT3G06640.1_Raf9	--
AT1G14000.1_VIK	--
AT2G31800.1_ILK3	--
AT2G43850.1_ILK1	--
AT3G59830.1_ILK2	--
AT3G58760.1_ILK4	--
AT4G18950.1_Raf27	--
AT2G31010.1_Raf13	--
AT3G58640.1_Raf15	--
AT5G01850.1_Raf31	--
AT5G50180.1_Raf34	--
AT3G27560.1	--
AT5G40540.1_Raf32	--
AT5G66710.1_Raf37	--
AT3G50720.1_Raf44	--
AT3G50730.1_Raf45	--
AT3G63260.1_Raf48/MRK1	--
AT3G22750.1_Raf39	--
AT4G14780.1_Raf26	--
AT3G01490.1_Raf38	--
AT5G50000.1_Raf33	--
AT4G38470.1_Raf30	--
AT2G17700.1_Raf21	--
AT4G35780.1_Raf29	--
AT2G24360.1_Raf22	--
AT4G31170.1_Raf28	--
AT1G62400.1_Raf19/HT1	--
AT3G46930.1_Raf43	--
AT5G58950.1_Raf36	--

AT1G04700.1_Raf16	G----I-----------------N---SSNGYFAE----SNVADSPR-------------
AT2G35050.1_Raf24	N----ATVLNIEE-K-RQSFEHER---KPPSRIEDKTYHELVQSAPVISSKND--TGQR-
AT1G16270.1_Raf18	D----VSRMSMVGNG-IRQYERTN---PPVHEFGNKL--GHIHSAPEASLCQDRSLGNFH
AT1G79570.1_Raf5	D----VSRFSTVENG-TSDIERTN---SSLHEFGNKL--NHVQSAPQALLSKDSSVGNLH
AT3G46920.1_Raf42	NLVSNPANLSASGLDYSSDLSNKRVSDGIISGFGSEQ------VSIDATNRNNPNLG---
AT5G57610.1_Raf35	---MDSGS-VNSSVT---
AT1G08720.1_EDR1	--
AT5G11850.1_Raf3	--
AT1G18160.1_Raf4	--
AT1G73660.1	--
AT4G24480.1_Raf6	--
AT5G03730.2_Raf1/CTR1	--
AT4G23050.2_Raf12	--
AT5G49470.2_Raf10	--
AT1G67890.1_Raf11	--
AT3G06620.1_Raf7	--
AT3G06630.1_Raf8	--
AT3G06640.1_Raf9	--
AT1G14000.1_VIK	--
AT2G31800.1_ILK3	--
AT2G43850.1_ILK1	--
AT3G59830.1_ILK2	--
AT3G58760.1_ILK4	--
AT4G18950.1_Raf27	--
AT2G31010.1_Raf13	--
AT3G58640.1_Raf15	--
AT5G01850.1_Raf31	--
AT5G50180.1_Raf34	--
AT3G27560.1	--
AT5G40540.1_Raf32	--
AT5G66710.1_Raf37	--
AT3G50720.1_Raf44	--
AT3G50730.1_Raf45	--
AT3G63260.1_Raf48/MRK1	--
AT3G22750.1_Raf39	--

```
AT4G14780.1_Raf26            ------------------------------------------------------------
AT3G01490.1_Raf38            ------------------------------------------------------------
AT5G50000.1_Raf33            ------------------------------------------------------------
AT4G38470.1_Raf30            ------------------------------------------------------------
AT2G17700.1_Raf21            ------------------------------------------------------------
AT4G35780.1_Raf29            ------------------------------------------------------------
AT2G24360.1_Raf22            ------------------------------------------------------------
AT4G31170.1_Raf28            ------------------------------------------------------------
AT1G62400.1_Raf19/HT1        ------------------------------------------------------------
AT3G46930.1_Raf43            ------------------------------------------------------------
AT5G58950.1_Raf36            ------------------------------------------------------------


AT1G04700.1_Raf16            -KMFQTAISDVYLPEVLKLLCSFGGRILQRPGDGKLRYIGGETRIISIRKHVGLNELMHK
AT2G35050.1_Raf24            RHSLVSSRASDSSLNRAKFLCSFGGKVIPRPRDQKLRYVGGETRIIRISKTISFQELMHK
AT1G16270.1_Raf18            GYAS--SSASGSLTAKVKVLCSFGGKILPRPGDSKLRYVGGETHIISIRKDISWQELRQK
AT1G79570.1_Raf5             GYKNTSSSASGSVTAKVKILCSFGGKILPRPGDSKLRYVGGETHIISIRKDISWQELRQK
AT3G46920.1_Raf42            ---NKRSDMDDEELEKVKFLCSYNGKIIPRPSDGMLRYVGGQTRIVSVKKNVRFDEFEQK
AT5G57610.1_Raf35            ---SLV-SSLNDEPHRVKFLCSFLGSILPRPQDGKLRYVGGETRIVSVNRDIRYEELMSK
AT1G08720.1_EDR1             ------------------------------------------------------------
AT5G11850.1_Raf3             --------------------------------------------------------MS--
AT1G18160.1_Raf4             -------------------------------------------------------MKM--
AT1G73660.1                  --------------------------VVLRPVDHCS-------SIWS--------MKM--
AT4G24480.1_Raf6             -----KSNGVGEDSNKEKESTVFSSNPLLSKSSAVS-------DLFS--------DDR--
AT5G03730.2_Raf1/CTR1        ------------------------------------------------------------
AT4G23050.2_Raf12            ---------------------------------------------------------M--
AT5G49470.2_Raf10            ---------------------------------------------------------M--
AT1G67890.1_Raf11            ---------------------------------------------------------M--
AT3G06620.1_Raf7             ------------------------------------------------------------
AT3G06630.1_Raf8             ------------------------------------------------------------
AT3G06640.1_Raf9             ---------------------------------------------MV--------KLL--
AT1G14000.1_VIK              ------------------------------------------------------------
AT2G31800.1_ILK3             ------------------------------------------------------------
AT2G43850.1_ILK1             ------------------------------------------------------------
AT3G59830.1_ILK2             ------------------------------------------------------------
AT3G58760.1_ILK4             ------------------------------------------------------------
AT4G18950.1_Raf27            ------------------------------------------------------------
AT2G31010.1_Raf13            ------------------------------------------------------------
AT3G58640.1_Raf15            ------------------------------------------------------------
AT5G01850.1_Raf31            ------------------------------------------------------------
AT5G50180.1_Raf34            ------------------------------------------------------------
AT3G27560.1                  ------------------------------------------------------------
AT5G40540.1_Raf32            ------------------------------------------------------------
AT5G66710.1_Raf37            ------------------------------------------------------------
AT3G50720.1_Raf44            ------------------------------------------------------------
AT3G50730.1_Raf45            ------------------------------------------------------------
AT3G63260.1_Raf48/MRK1       ------------------------------------------------------------
AT3G22750.1_Raf39            ------------------------------------------------------------
AT4G14780.1_Raf26            ------------------------------------------------------------
AT3G01490.1_Raf38            ------------------------------------------------------------
AT5G50000.1_Raf33            ------------------------------------------------------------
AT4G38470.1_Raf30            ------------------------------------------------------------
AT2G17700.1_Raf21            ------------------------------------------------------------
AT4G35780.1_Raf29            ------------------------------------------------------------
AT2G24360.1_Raf22            ------------------------------------------------------------
AT4G31170.1_Raf28            ------------------------------------------------------------
AT1G62400.1_Raf19/HT1        ------------------------------------------------------------
AT3G46930.1_Raf43            ------------------------------------------------------------
AT5G58950.1_Raf36            ------------------------------------------------------------


AT1G04700.1_Raf16            TYALCNHPHTIKYQLPGEDLDALISVCSDEDLLHMIEEYQEAE--T-------------K
AT2G35050.1_Raf24            MKEIFPEARTIKYQLPGEDLDALVSVSSDEDLQNMMEECIVF---G-------------N
AT1G16270.1_Raf18            VLEIYYRTHVVKYQLPGEDLDALVSVSCDEDLLNMMEEYNEME--N-------------R
AT1G79570.1_Raf5             ILEIYYQTRVVKYQLPGEDLDALVSVSSEEDLQNMLEEYNEME--N-------------R
AT3G46920.1_Raf42            MIQVYGHPVVVKYQLPDEDLDALVSVSSSEDIDNMMEEFEKLVERS-------------S
AT5G57610.1_Raf35            MRELYDGAAVLKYQQPDEDLDALVSVVNDDDVTNMMEEYDKLG--S-------------G
AT1G08720.1_EDR1             ----------------------------------MKHIFKKLHRGG--------NQEQQN
```

```
AT5G11850.1_Raf3          ---------------------------------KMKHLLRKLHIGG--------SSGVGG
AT1G18160.1_Raf4          ---------------------------------NMKKFLKKLRITP--------NQRDDG
AT1G73660.1               ---------------------------------NMKNFLKKLHISP--------NQSDEA
AT4G24480.1_Raf6          -------------------------KSEKKHQQQLAAFYEWLAEKKANLSRSSSTTTTHG
AT5G03730.2_Raf1/CTR1     ------------------------------KAKAERGGFDWDPSGG--------------
AT4G23050.2_Raf12         -------------------------AGNNSESSLYQVLVEWCQRM---------------
AT5G49470.2_Raf10         -------------------------EKTT---PPAEELLKKIREL---------------
AT1G67890.1_Raf11         -------------------------ENPN---PPAEKLLKKIREL---------------
AT3G06620.1_Raf7          --------------------------MEN---PPAEELLKKILEL---------------
AT3G06630.1_Raf8          ---------------------------------MEEELLKKMLEL---------------
AT3G06640.1_Raf9          -------------------------QDPI---TPNKELLKKMIEL---------------
AT1G14000.1_VIK           ------------------------------------------------------------
AT2G31800.1_ILK3          ------------------------------------------------------------
AT2G43850.1_ILK1          ------------------------------------------------------------
AT3G59830.1_ILK2          ------------------------------------------------------------
AT3G58760.1_ILK4          ------------------------------------------------------------
AT4G18950.1_Raf27         ------------------------------------------------------------
AT2G31010.1_Raf13         ------------------------------------------------------------
AT3G58640.1_Raf15         ------------------------------------------------------------
AT5G01850.1_Raf31         ------------------------------------------------------------
AT5G50180.1_Raf34         ------------------------------------------------------------
AT3G27560.1               ------------------------------------------------------------
AT5G40540.1_Raf32         ------------------------------------------------------------
AT5G66710.1_Raf37         ------------------------------------------------------------
AT3G50720.1_Raf44         ------------------------------------------------------------
AT3G50730.1_Raf45         ------------------------------------------------------------
AT3G63260.1_Raf48/MRK1    ------------------------------------------------------------
AT3G22750.1_Raf39         ------------------------------------------------------------
AT4G14780.1_Raf26         ------------------------------------------------------------
AT3G01490.1_Raf38         ------------------------------------------------------------
AT5G50000.1_Raf33         ------------------------------------------------------------
AT4G38470.1_Raf30         ------------------------------------------------------------
AT2G17700.1_Raf21         ------------------------------------------------------------
AT4G35780.1_Raf29         ------------------------------------------------------------
AT2G24360.1_Raf22         ------------------------------------------------------------
AT4G31170.1_Raf28         ------------------------------------------------------------
AT1G62400.1_Raf19/HT1     ------------------------------------------------------------
AT3G46930.1_Raf43         ------------------------------------------------------------
AT5G58950.1_Raf36         ------------------------------------------------------------


AT1G04700.1_Raf16         AGSQRIR-----------VFLVPST--ESSESPKIFHERNMNINRNTNQQTDIDHYQYVS
AT2G35050.1_Raf24         GGSEKPR-----------MFLFSSS--DIEEAQFVM-----------EHAEGDSEVQYVV
AT1G16270.1_Raf18         GGSQKLR-----------MFLFSVS--DLDGALLGV-----------NKSDVDSEFQYVV
AT1G79570.1_Raf5          GGSQKLR-----------MFLFSIS--DMDDALLGV-----------NKNDGDSEFQYVV
AT3G46920.1_Raf42         DGSGKLR-----------VFLFDASSSEVDDSFGIL-E---------YGDGVDIGQRYVE
AT5G57610.1_Raf35         DGFTRLR-----------IFLFSTP--EQDGSLHYV-E---------RDDQRESERRYVD
AT1G08720.1_EDR1          RTNDAA-----------------PP-SDQNRI-HVS----------ANP-----------
AT5G11850.1_Raf3          -G-----FADHHRL------DD-------------S----------T-------------
AT1G18160.1_Raf4          EGSVSNRSNKS---------SDAEP-SPSDSL-RSQ----------DNS----------E
AT1G73660.1               EGSISTTKSNHHKS------IDVSS-SSSPRS-HHS----------NSP----------E
AT4G24480.1_Raf6          RGVKPTRFSMSSDADEERELLLSSP-ADPAPL-PAT----------SSPDSIIDSARTVN
AT5G03730.2_Raf1/CTR1     -GGGDH--------------------------------------------------RLNN
AT4G23050.2_Raf12         ------------------------------------------------------------
AT5G49470.2_Raf10         ------------------------------------------------------------
AT1G67890.1_Raf11         ------------------------------------------------------------
AT3G06620.1_Raf7          ------------------------------------------------------------
AT3G06630.1_Raf8          ------------------------------------------------------------
AT3G06640.1_Raf9          ------------------------------------------------------------
AT1G14000.1_VIK           ------------------------------------------------------------
AT2G31800.1_ILK3          ------------------------------------------------------------
AT2G43850.1_ILK1          ------------------------------------------------------------
AT3G59830.1_ILK2          ------------------------------------------------------------
AT3G58760.1_ILK4          ------------------------------------------------------------
AT4G18950.1_Raf27         ------------------------------------------------------------
AT2G31010.1_Raf13         ------------------------------------------------------------
AT3G58640.1_Raf15         ------------------------------------------------------------
AT5G01850.1_Raf31         ------------------------------------------------------------
```

```
AT5G50180.1_Raf34          ------------------------------------------------------------
AT3G27560.1                ------------------------------------------------------------
AT5G40540.1_Raf32          ------------------------------------------------------------
AT5G66710.1_Raf37          ------------------------------------------------------------
AT3G50720.1_Raf44          ------------------------------------------------------------
AT3G50730.1_Raf45          ------------------------------------------------------------
AT3G63260.1_Raf48/MRK1     ------------------------------------------------------------
AT3G22750.1_Raf39          ------------------------------------------------------------
AT4G14780.1_Raf26          ------------------------------------------------------------
AT3G01490.1_Raf38          ------------------------------------------------------------
AT5G50000.1_Raf33          ------------------------------------------------------------
AT4G38470.1_Raf30          ------------------------------------------------------------
AT2G17700.1_Raf21          ------------------------------------------------------------
AT4G35780.1_Raf29          ------------------------------------------------------------
AT2G24360.1_Raf22          ------------------------------------------------------------
AT4G31170.1_Raf28          ------------------------------------------------------------
AT1G62400.1_Raf19/HT1      ------------------------------------------------------------
AT3G46930.1_Raf43          ------------------------------------------------------------
AT5G58950.1_Raf36          ------------------------------------------------------------


AT1G04700.1_Raf16          ALNGIVDVSPQKS-------SSGQSGTSQ-TT------QFGNASE---------------
AT2G35050.1_Raf24          AVNGMDLSSR-RS-------SLGLSP---PGNNLD-ELLHGNFDRKIDRAA-----TEPA
AT1G16270.1_Raf18          AVNDMDLGSR-SN-------STLNGLDSSSANNLA-ELDVRNTEG-IN------------
AT1G79570.1_Raf5           AVNGMDIGSG-KN-------STLLGLDSSSANNLA-ELDVRNTEG-INTIA-----GDVV
AT3G46920.1_Raf42          AVNGVVVSKES-V-------ASGSSNPNSDFSGVD-VVDSL-------------------
AT5G57610.1_Raf35          ALNNLIEGTDF-R-------KLQQYPDSPRFN----LVDDF-------------------
AT1G08720.1_EDR1           ---PQATPSSVTETLPV--AGATSSMA---------SPA---------------------
AT5G11850.1_Raf3           -R-PMIDPSPILSTSPS--PASTSSVS---------SSGFGNASTTM----PRLDTFE--
AT1G18160.1_Raf4           FK-PFLGLSNWLSSVTH--RKSPS------------SSNATNSKEDD----TTMEHGG--
AT1G73660.1                IK-PFSGLSNWLSSVGH--RKIPS------------PPNSFNAKNRA----ATVDDTV--
AT4G24480.1_Raf6           IH-ERNIDRSFDREVSLPRMSSESSFAGSFFSGTTVDGNFSNFSSHTDARETST--TTLV
AT5G03730.2_Raf1/CTR1      QP-NRVGNNMYASSLGLQRQSSGSSFGESSLSGDYYMPTLSAAANEIESVGFPQDDGFRL
AT4G23050.2_Raf12          ------------------------------------------------------------
AT5G49470.2_Raf10          ------------------------------------------------------------
AT1G67890.1_Raf11          ------------------------------------------------------------
AT3G06620.1_Raf7           ------------------------------------------------------------
AT3G06630.1_Raf8           ------------------------------------------------------------
AT3G06640.1_Raf9           ------------------------------------------------------------
AT1G14000.1_VIK            ------------------------------------------------------------
AT2G31800.1_ILK3           ------------------------------------------------------------
AT2G43850.1_ILK1           ------------------------------------------------------------
AT3G59830.1_ILK2           ------------------------------------------------------------
AT3G58760.1_ILK4           ------------------------------------------------------------
AT4G18950.1_Raf27          ------------------------------------------------------------
AT2G31010.1_Raf13          -------------------------------------------------MEERRDDES--
AT3G58640.1_Raf15          -------------------------------------------------MGETG-DDA--
AT5G01850.1_Raf31          ------------------------------------------------------------
AT5G50180.1_Raf34          ------------------------------------------------------------
AT3G27560.1                ------------------------------------------------------------
AT5G40540.1_Raf32          ------------------------------------------------------------
AT5G66710.1_Raf37          ------------------------------------------------------------
AT3G50720.1_Raf44          ------------------------------------------------------------
AT3G50730.1_Raf45          ------------------------------------------------------------
AT3G63260.1_Raf48/MRK1     ------------------------------------------------------------
AT3G22750.1_Raf39          ------------------------------------------------------------
AT4G14780.1_Raf26          ------------------------------------------------------------
AT3G01490.1_Raf38          ------------------------------------------------------------
AT5G50000.1_Raf33          ------------------------------------------------------------
AT4G38470.1_Raf30          ------------------------------------------------------------
AT2G17700.1_Raf21          ------------------------------------------------------------
AT4G35780.1_Raf29          ------------------------------------------------------------
AT2G24360.1_Raf22          ------------------------------------------------------------
AT4G31170.1_Raf28          ------------------------------------------------------------
AT1G62400.1_Raf19/HT1      ------------------------------------------------------------
AT3G46930.1_Raf43          ------------------------------------------------------------
AT5G58950.1_Raf36          ------------------------------------------------------------
```

```
AT1G04700.1_Raf16        -F--SP-----TFHLR---DSPTSVHTWEHKDSNSP---TFMKPYG--------------
AT2G35050.1_Raf24        VASLTP-----LAGNESLPASQTSQPVTGFSTGNEP---FSQPYLG--------------
AT1G16270.1_Raf18        GVGPSQ-----LTGID------FQQSSMQYSESAPP---TSFAQYP--------------
AT1G79570.1_Raf5         GVGASQ-----LMVNG------FQQTSAQQSESIPP---SSSLHYS--------------
AT3G46920.1_Raf42        GVG----------------QSDFVATTW------TSSNFSPQTYHSNVSRLVPPDPRSSA
AT5G57610.1_Raf35        SMV----------------EPMLNQLSIETGGGSQRGNEIPTAQYSNLHQLR--------
AT1G08720.1_EDR1         ------------------PTAASNRADY------MSSEEEYQVQLALAISASNSQSS---
AT5G11850.1_Raf3         PVGRD--------------LTAVDGVDF------NLMEEEYQVQLAMAISVSDPDPR---
AT1G18160.1_Raf4         PVGSESG---M------QGLGSSSNSKD------PEVEEEYQIQLALELSA-----R---
AT1G73660.1              VVNGS--------------EHVDLGSKD------PAVEEENQIQLALELSA-----R---
AT4G24480.1_Raf6         SVNKEEEE-------VEVREQGKEQSLA------QKSREGYYLQVTLAKWLSSQANL---
AT5G03730.2_Raf1/CTR1    GFGGGGGDLRIQMAADSAGGSSSGKSWA------QQTEESYQLQLALALRLSSEATC---
AT4G23050.2_Raf12        --------------------------------------ETSQARLREDV-----------
AT5G49470.2_Raf10        --------------------------------------EESQEHLKREM-----------
AT1G67890.1_Raf11        --------------------------------------EESQEDLKREM-----------
AT3G06620.1_Raf7         --------------------------------------EESQEHLKQEM-----------
AT3G06630.1_Raf8         --------------------------------------EQSQELLKQEM-----------
AT3G06640.1_Raf9         --------------------------------------EKSQEHLMQEM-----------
AT1G14000.1_VIK          ------------------------------------------------------------
AT2G31800.1_ILK3         ------------------------------------------------------------
AT2G43850.1_ILK1         ------------------------------------------------------------
AT3G59830.1_ILK2         ------------------------------------------------------------
AT3G58760.1_ILK4         ------------------------------------------------------------
AT4G18950.1_Raf27        ------------------------------------------------------------
AT2G31010.1_Raf13        ---------------SPTHQG--------------SELAERVKLLSFES-----------
AT3G58640.1_Raf15        ---------------GPSEQGPSNQTWW------PSEFVEKFGSVYLGS-----------
AT5G01850.1_Raf31        ------------------------------------------------------------
AT5G50180.1_Raf34        ------------------------------------------------------------
AT3G27560.1              ------------------------------------------------------------
AT5G40540.1_Raf32        ------------------------------------------------------------
AT5G66710.1_Raf37        ------------------------------------------------------------
AT3G50720.1_Raf44        ------------------------------------------------------------
AT3G50730.1_Raf45        ------------------------------------------------------------
AT3G63260.1_Raf48/MRK1   ------------------------------------------------------------
AT3G22750.1_Raf39        ------------------------------------------------------------
AT4G14780.1_Raf26        ------------------------------------------------------------
AT3G01490.1_Raf38        ------------------------------------------------------------
AT5G50000.1_Raf33        ------------------------------------------------------------
AT4G38470.1_Raf30        ------------------------------------------------------------
AT2G17700.1_Raf21        ------------------------------------------------------------
AT4G35780.1_Raf29        ------------------------------------------------------------
AT2G24360.1_Raf22        ------------------------------------------------------------
AT4G31170.1_Raf28        ------------------------------------------------------------
AT1G62400.1_Raf19/HT1    ------------------------------------------------------------
AT3G46930.1_Raf43        ------------------------------------------------------------
AT5G58950.1_Raf36        ------------------------------------------------------------


AT1G04700.1_Raf16        ------------------NTN------------------AVHFMPKMQIPR--NSFGQQS
AT2G35050.1_Raf24        ------------------QQLQFPGLGNHQIYTSG-HMASIGYIDEKR-SA--PLHVQPQ
AT1G16270.1_Raf18        ------------------QSI--PHNGAFQFQQAVPPNATLQYAPSNP-PS--------S
AT1G79570.1_Raf5         ------------------QSI--PLNAAYQLQQSVPPSSALHYPQSIT-PG--------S
AT3G46920.1_Raf42        YVVPMTVHADPPHSFQLETVSEKPIVGKMQQQQQGYTTPSEHHPPAYV-ESRQEALRQPD
AT5G57610.1_Raf35        ----------------------IPRVGSGQMLAQRYGEVEGTWSPFYS-PRH-HGHHDPR
AT1G08720.1_EDR1         --------EDP-E--------------KHQIRA----------ATLLS-----LGSHQRM
AT5G11850.1_Raf3         --------ENA-D--------------TAQLDA----------AKRIS-----LGVSAPV
AT1G18160.1_Raf4         --------EDP-E--------------AAQIEA----------MKQFS-----LGSR-PS
AT1G73660.1              --------EDP-E--------------ATQIEA----------IKQFS-----LGS---C
AT4G24480.1_Raf6         --------ACE-------------------------------------------------
AT5G03730.2_Raf1/CTR1    --------ADD----------------PNFLDP------------------------VPD
AT4G23050.2_Raf12        ----------------------------DDLLL----------QEESR-----TGKE---
AT5G49470.2_Raf10        ----------------------------SRLKV----------SAEMK-----QRSH---
AT1G67890.1_Raf11        ----------------------------SKLKV----------SAEIK-----RRSH---
AT3G06620.1_Raf7         ----------------------------SRLKV----------STELR-----QRSH---
AT3G06630.1_Raf8         ----------------------------SRLKL----------STELR-----QPSH---
AT3G06640.1_Raf9         ----------------------------SRLKV----------STELR-----KE-----
AT1G14000.1_VIK          ------------------------------------------------------------
```

```
AT2G31800.1_ILK3              ------------------------------------------------------------
AT2G43850.1_ILK1              ------------------------------------------------------------
AT3G59830.1_ILK2              ------------------------------------------------------------
AT3G58760.1_ILK4              ------------------------------------------------------------
AT4G18950.1_Raf27             ------------------------------------------------------------
AT2G31010.1_Raf13             -------------------------------------------Q-GEA-----LSKDSP-
AT3G58640.1_Raf15             -------------------------------------------QEETS-----STKDSP-
AT5G01850.1_Raf31             ------------------------------------------------------------
AT5G50180.1_Raf34             ------------------------------------------------------------
AT3G27560.1                   ------------------------------------------------------------
AT5G40540.1_Raf32             ------------------------------------------------------------
AT5G66710.1_Raf37             ------------------------------------------------------------
AT3G50720.1_Raf44             ------------------------------------------------------------
AT3G50730.1_Raf45             ------------------------------------------------------------
AT3G63260.1_Raf48/MRK1        ------------------------------------------------------------
AT3G22750.1_Raf39             ------------------------------------------------------------
AT4G14780.1_Raf26             ------------------------------------------------------------
AT3G01490.1_Raf38             ------------------------------------------------------------
AT5G50000.1_Raf33             ------------------------------------------------------------
AT4G38470.1_Raf30             ------------------------------------------------------------
AT2G17700.1_Raf21             ------------------------------------------------------------
AT4G35780.1_Raf29             ------------------------------------------------------------
AT2G24360.1_Raf22             ------------------------------------------------------------
AT4G31170.1_Raf28             ------------------------------------------------------------
AT1G62400.1_Raf19/HT1         ------------------------------------------------------------
AT3G46930.1_Raf43             ------------------------------------------------------------
AT5G58950.1_Raf36             ------------------------------------------------------------


AT1G04700.1_Raf16             PPTSPFSVHKRA----------NTDVP--------YFADQNGFFDPYLAAPNFP-QQ-NR
AT2G35050.1_Raf24             PHYIPYSVNPETPLESLVPHYPQKPEQ--------GFLREE-----------------QI
AT1G16270.1_Raf18             SVHYPQSILPNS-----TLQYPQSIS-----------SSSYGLYPQYYGETEQ-------
AT1G79570.1_Raf5              SLQYPQSITPGS-----SYQYPQSIIP--------GSASSYGIYPQYYGHVVQH-GERER
AT3G46920.1_Raf42             IVHSPIQLLPSSTS-L-FSQQPFQDSP---------LSVSSHQ---FLPAAHMS-----M
AT5G57610.1_Raf35             T----FQEFPSSPSSA-RYRMPYGEIPD------KGLDRMPEE---YV------------
AT1G08720.1_EDR1              DSRRDSSEVVAQ---RLSRQYWEYGVLDYEEKVVDSFYDVYSL-------STDSAKQGEM
AT5G11850.1_Raf3              TDADS----AVD---FLSLRYWGHKVINYDQKVRDGFYDVYGI-------TSNSLSQGKM
AT1G18160.1_Raf4              APENT----PAE---LMAYRYWNYNCLGYDDKIVDGFYDLCGV-------MNES-SLKRI
AT1G73660.1                   APENS----PAE---LIAYRYWNYNCLGYDDKILDGFYDLYGV-------LNAS-SAERI
AT4G24480.1_Raf6              ----SVHIQSTE---SISYRFWVSGCLSYSDKISDGFYSILGMDPYLWLMCNNSEDGKRI
AT5G03730.2_Raf1/CTR1         ESALRTSPSSAE---TVSHRFWVNGCLSYYDKVPDGFYMMNGLDPYIWTLCIDLHESGRI
AT4G23050.2_Raf12             -SATGLETDTEK---EAEVEVEAEAADSWDNPT---------------------------
AT5G49470.2_Raf10             -SASP------Q---RPVRRNSNDGTPMWRKTGAASFRHASPL-------RKESHAKV--
AT1G67890.1_Raf11             -SSSP------K---RPSRRNSGEGTPLWRKTGAASFRHASPL-------RKESHSKDGV
AT3G06620.1_Raf7              -SVSP------H---RPARRNIGEGAPSWRKSGAASFRNASPL-------RKESRIQNSM
AT3G06630.1_Raf8              -PVLP------R---RPLRRIQ-----------------------------------GSM
AT3G06640.1_Raf9              ------------------SRIQ-----------------------------------CSM
AT1G14000.1_VIK               ------------------------------------------------------------
AT2G31800.1_ILK3              ------------------------------------------------------------
AT2G43850.1_ILK1              ------------------------------------------------------------
AT3G59830.1_ILK2              ------------------------------------------------------------
AT3G58760.1_ILK4              ------------------------------------------------------------
AT4G18950.1_Raf27             ------------------------------------------------------------
AT2G31010.1_Raf13             -RSVEQDCSPGQ---RASQHLWDTGIL--SEPIPNGFYSVVPD-------KRVKELYNRL
AT3G58640.1_Raf15             -RNLGQDGLPSS---TASNILWSTGSL--SEPIPNGFYSVIPD-------NRLKQLFNNI
AT5G01850.1_Raf31             ------------------------------------------------------------
AT5G50180.1_Raf34             ------------------------------------------------------------
AT3G27560.1                   ------------------------------------------------------------
AT5G40540.1_Raf32             ------------------------------------------------------------
AT5G66710.1_Raf37             ------------------------------------------------------------
AT3G50720.1_Raf44             ------------------------------------------------------------
AT3G50730.1_Raf45             ------------------------------------------------------------
AT3G63260.1_Raf48/MRK1        ------------------------------------------------------------
AT3G22750.1_Raf39             ------------------------------------------------------------
AT4G14780.1_Raf26             ------------------------------------------------------------
AT3G01490.1_Raf38             ------------------------------------------------------------
AT5G50000.1_Raf33             ------------------------------------------------------------
AT4G38470.1_Raf30             ------------------------------------------------------------
```

```
AT2G17700.1_Raf21           ------------------------------------------------------------
AT4G35780.1_Raf29           ------------------------------------------------------------
AT2G24360.1_Raf22           ------------------------------------------------------------
AT4G31170.1_Raf28           ------------------------------------------------------------
AT1G62400.1_Raf19/HT1       ------------------------------------------------------------
AT3G46930.1_Raf43           ------------------------------------------------------------
AT5G58950.1_Raf36           ------------------------------------------------------------


AT1G04700.1_Raf16           FFFETTTQKQ----------------------------------KHPEVNLHDRRPSDDI
AT2G35050.1_Raf24           FHVQDPETSS--KE----AKMRRDDSFQKVNDHPISTVESNLSAKEPKMRRESSTPRVNE
AT1G16270.1_Raf18           FPMQYHDHNS--SN------------YS------IP-----IPFP------------GQP
AT1G79570.1_Raf5            FPL-YPDHS---SN------------YSAIGETTSS-----IPIQG-HVSQQ--GGWAEG
AT3G46920.1_Raf42           APL-NSQISS--TPVLINPVMQTQE--NLLGNYHAA--QKLVPLPTEP----RNTAYQGT
AT5G57610.1_Raf35           ----RPQASH--H-----PFYEHQA--------HIP--DSVVWVPAGAMPPESKGGFPGN
AT1G08720.1_EDR1            PSLEDLESNHG--TPGFEAVV--------------------VNRPID----------SSL
AT5G11850.1_Raf3            PLLVDLQAISISDNVDYEVIL--------------------VNRLID----------PEL
AT1G18160.1_Raf4            PPLVDLQGTLVSDGVTWDAVL--------------------VNSSKD----------SNL
AT1G73660.1                 PPLLDLQGTPVSDGVTWEAVL--------------------VNRSGD----------SNL
AT4G24480.1_Raf6            PSLLLLKETE-PNDTSMEVVL--------------------IDRRED----------SRL
AT5G03730.2_Raf1/CTR1       PSIESLRAVDSGVDSSLEAII--------------------VDRRSD----------PAF
AT4G23050.2_Raf12           ------------------------------------------------------------
AT5G49470.2_Raf10           -------A----------------------------------------------------
AT1G67890.1_Raf11           ------------------------------------------------------------
AT3G06620.1_Raf7            ----RLRS----------------------------------------------------
AT3G06630.1_Raf8            ------------------------------------------------------------
AT3G06640.1_Raf9            ------------------------------------------------------------
AT1G14000.1_VIK             ------------------------------------------------------------
AT2G31800.1_ILK3            ------------------------------------------------------------
AT2G43850.1_ILK1            ------------------------------------------------------------
AT3G59830.1_ILK2            ------------------------------------------------------------
AT3G58760.1_ILK4            ------------------------------------------------------------
AT4G18950.1_Raf27           ------------------------------------------------------------
AT2G31010.1_Raf13           PTPSELHAL-GEEGVRIEVIL--------------------VDFQKD----------KKL
AT3G58640.1_Raf15           PTLEDLHAL-GDEGLKADVIL--------------------VDFQKD----------KKL
AT5G01850.1_Raf31           ------------------------------------------------------------
AT5G50180.1_Raf34           ------------------------------------------------------------
AT3G27560.1                 ------------------------------------------------------------
AT5G40540.1_Raf32           ------------------------------------------------------------
AT5G66710.1_Raf37           ------------------------------------------------------------
AT3G50720.1_Raf44           ------------------------------------------------------------
AT3G50730.1_Raf45           ------------------------------------------------------------
AT3G63260.1_Raf48/MRK1      ------------------------------------------------------------
AT3G22750.1_Raf39           ------------------------------------------------------------
AT4G14780.1_Raf26           ------------------------------------------------------------
AT3G01490.1_Raf38           ------------------------------------------------------------
AT5G50000.1_Raf33           ------------------------------------------------------------
AT4G38470.1_Raf30           ------------------------------------------------------------
AT2G17700.1_Raf21           ------------------------------------------------------------
AT4G35780.1_Raf29           ------------------------------------------------------------
AT2G24360.1_Raf22           ------------------------------------------------------------
AT4G31170.1_Raf28           ------------------------------------------------------------
AT1G62400.1_Raf19/HT1       ------------------------------------------------------------
AT3G46930.1_Raf43           ------------------------------------------------------------
AT5G58950.1_Raf36           ------------------------------------------------------------


AT1G04700.1_Raf16           YPHGQAY--------------------------IGAEKMTLKKNAL---------SDPQL
AT2G35050.1_Raf24           YPVSSMPSDLIVPDDL-----------PK----EEAPIVTQTSSSTPDPSSSTLSEKSLR
AT1G16270.1_Raf18           YPHPGI---------T-----------QQ----N-APVQV---------------EEPNI
AT1G79570.1_Raf5            YPYPGS---------T-----------PK----S-TQALA---------------EEQKV
AT3G46920.1_Raf42           -ISPGIPFDGYGGSQV-----------PP----SNH-------VVLPDG--------SF-
AT5G57610.1_Raf35           -VLHGGP-----------------------------------------------------
AT1G08720.1_EDR1            HELLEIAECIALGCSTT-----SVSVLVQRLAELVTEHMGGSAEDS----------SIVL
AT5G11850.1_Raf3            QELERRVFALASECPD-FAPGQVSSDLTQKIANIVVEQMGGPVENA----------DEAL
AT1G18160.1_Raf4            LRLEQMALDIAAKSKSASSSGFVNSELVRQLAVLVADYMGGPVLDP----------DSTL
AT1G73660.1                 LRLEQMALDIAAKSRSVSSSGFVNSELVRKLAILVGDYMGGPVVHP----------ESML
AT4G24480.1_Raf6            KELEDKAHELYCSSDN-------MLVLVEKLGRLVAVYMGGNFQVE---------QGDLQ
```

```
AT5G03730.2_Raf1/CTR1       KELHNRVHDISCSCIT-------TKEVVDQLAKLICNRMGGPVIMG---------EDELV
AT4G23050.2_Raf12           ----------ATW-----ERAVSGFYFADSAYRTLMDSMGHAIHVTS-A------ASGEI
AT5G49470.2_Raf10           ----------GGG-----GEGQSAGKFTDKQYLNILQSMAQAVHVFD--------LNGQI
AT1G67890.1_Raf11           ----------AGG-----GDGPSAGKFTDKQYLNILQSMAQAVHVFD--------LNGQI
AT3G06620.1_Raf7            ----------EVG-----GGGPSAGKFTDKQYLNILQSMAQAVHAFD--------LNMRI
AT3G06630.1_Raf8            ------------------NSNPSPGKFTDKQYLNILQSLAQSVHVLD--------LNTRI
AT3G06640.1_Raf9            ------------------NLRPSPWKFTHKQYLNILQSMAQSVHAFD--------LNMRI
AT1G14000.1_VIK             ------------------------------------------------------------
AT2G31800.1_ILK3            ------------------------------------------------------------
AT2G43850.1_ILK1            ------------------------------------------------------------
AT3G59830.1_ILK2            ------------------------------------------------------------
AT3G58760.1_ILK4            ------------------------------------------------------------
AT4G18950.1_Raf27           ------------------------------------------------------------
AT2G31010.1_Raf13           AMLKQLITTLVSGSGT------NPALVIKKIAGTVSDFYKRPTLESP-------------
AT3G58640.1_Raf15           FRQKQLITKLVSGLNS------KPATIIKKIAGLVADVYKQSTLQSP-------------
AT5G01850.1_Raf31           ------------------------------------------------------------
AT5G50180.1_Raf34           ------------------------------------------------------------
AT3G27560.1                 ------------------------------------------------------------
AT5G40540.1_Raf32           ------------------------------------------------------------
AT5G66710.1_Raf37           ------------------------------------------------------------
AT3G50720.1_Raf44           ------------------------------------------------------------
AT3G50730.1_Raf45           ------------------------------------------------------------
AT3G63260.1_Raf48/MRK1      ------------------------------------------------------------
AT3G22750.1_Raf39           ------------------------------------------------------------
AT4G14780.1_Raf26           ------------------------------------------------------------
AT3G01490.1_Raf38           ------------------------------------------------------------
AT5G50000.1_Raf33           ------------------------------------------------------------
AT4G38470.1_Raf30           ------------------------------------------------------------
AT2G17700.1_Raf21           ------------------------------------------------------------
AT4G35780.1_Raf29           ------------------------------------------------------------
AT2G24360.1_Raf22           ------------------------------------------------------------
AT4G31170.1_Raf28           ------------------------------------------------------------
AT1G62400.1_Raf19/HT1       ------------------------------------------------------------
AT3G46930.1_Raf43           ------------------------------------------------------------
AT5G58950.1_Raf36           ------------------------------------------------------------


AT1G04700.1_Raf16           HDESQINNGLEAFTKQPWKILRK---NLRVV-ATSKWEDSDDIYFNNPEGKRCKELELTK
AT2G35050.1_Raf24           KSEDHVENNLSAKEPKMRKEHSTTRVNEYSVSSVSSDSMVPDQALKEEAP---ISMKISN
AT1G16270.1_Raf18           KPETKVRDY---VEPENRHILATNHQNPPQA---------DDTEVKNREP--SV---ATT
AT1G79570.1_Raf5            SSDMKIREE---VEPENRKTPGNDHQNPPQI---------DDVEVRNHNQ--VREMAVAT
AT3G46920.1_Raf42           YQQVTMAES-------------FQRVNDCHMCQTSFPHMHSDPIMREGND------GSTM
AT5G57610.1_Raf35           -------GG-------------YEGGNGCENCRVPYHRNH--QLLEQSNI------GNNG
AT1G08720.1_EDR1            ARWTEKSSEFKAALNTCVFPIGFVKIGISRH---------RALLFKVLA--------DSV
AT5G11850.1_Raf3            RRWMLRSYELRNSLNTTILPLGRVNVGLARH---------RALLFKVLA--------DRI
AT1G18160.1_Raf4            RAWWSLSYSLKATLRSMVLPLGSLTIGLARH---------RALLFKVLC--------DSV
AT1G73660.1                 RAWRSLSYSLKATLGSMVLPLGSLTIGLARH---------RALLFKVLC--------DSV
AT4G24480.1_Raf6            KRWKLVSNRLKEFRKCIILPIGSLTMGLCRH---------RAILFKKLA--------DYI
AT5G03730.2_Raf1/CTR1       PMWKECIDGLKEIFK-VVVPIGSLSVGLCRH---------RALLFKVLA--------DII
AT4G23050.2_Raf12           TFWSRSAENLYHWYAEEVVGYRTIDVLVTEE---------Y------RN--------SLT
AT5G49470.2_Raf10           IFWNSMAEKLYGFSASEALGKDPIDILVDVQ---------D------AS--------VAQ
AT1G67890.1_Raf11           IFWNSMAEKLYGFSAAEALGKDSINILVDGQ---------D------AA--------VAK
AT3G06620.1_Raf7            IFWNAMAEKVYGYSAAEALGENPINVIADDR---------D------AA--------FAM
AT3G06630.1_Raf8            IFWNAMSEKLYGYSAAEVVGRNPVHVIVDDQ---------N------AA--------FAL
AT3G06640.1_Raf9            IFWNAMAEKVYGYSAAEAVGQNPIDVMVDDR---------D------AP--------FAM
AT1G14000.1_VIK             ------------------------------------------------------------
AT2G31800.1_ILK3            ------------------------------------------------------------
AT2G43850.1_ILK1            ------------------------------------------------------------
AT3G59830.1_ILK2            ------------------------------------------------------------
AT3G58760.1_ILK4            ------------------------------------------------------------
AT4G18950.1_Raf27           ------------------------------------------------------------
AT2G31010.1_Raf13           -SKLALEENAFLFENHGAQLLGQIKRGCCRA---------RAILFKVLA--------DTV
AT3G58640.1_Raf15           -AKS-----TQSFENCGIQLLGQIKHGSCRP---------RAILFKVLA--------DTV
AT5G01850.1_Raf31           ------------------------------------------------------------
AT5G50180.1_Raf34           ------------------------------------------------------------
AT3G27560.1                 ------------------------------------------------------------
AT5G40540.1_Raf32           ------------------------------------------------------------
AT5G66710.1_Raf37           ------------------------------------------------------------
```

```
AT3G50720.1_Raf44           ------------------------------------------------------------
AT3G50730.1_Raf45           ------------------------------------------------------------
AT3G63260.1_Raf48/MRK1      ------------------------------------------------------------
AT3G22750.1_Raf39           ------------------------------------------------------------
AT4G14780.1_Raf26           ------------------------------------------------------------
AT3G01490.1_Raf38           ------------------------------------------------------------
AT5G50000.1_Raf33           ------------------------------------------------------------
AT4G38470.1_Raf30           --------------------MVMEDNESCAS---------RVIFDALPT--------SQA
AT2G17700.1_Raf21           ------------------MTIK-DESESCGS---------RAVVASPS-----------Q
AT4G35780.1_Raf29           ------------------MAIKEETEESCGS---------RAVVASIT-----------K
AT2G24360.1_Raf22           ------------------------------------------------------------
AT4G31170.1_Raf28           ------------------------------------------------------------
AT1G62400.1_Raf19/HT1       ------------------------------------------------------------
AT3G46930.1_Raf43           ------------------------------------------------------------
AT5G58950.1_Raf36           ------------------------------------------------------------


AT1G04700.1_Raf16           EVP--------NS---WINRDNNP---DSFDQATKKQ--D--------------------
AT2G35050.1_Raf24           STPDP-KSLVYPEKSLRTSQ-EKT---GAFDT---TN--E--------------------
AT1G16270.1_Raf18           VPSQDAAHMLPPRRDTRQNTPVKP---STYRDAVITE--Q--------------------
AT1G79570.1_Raf5            TPPSQDAHLLPPSRDPRQNTTAKP---ATYRDAVITG--Q--------------------
AT3G46920.1_Raf42           YVPYVSSAFYASRPDDIMRIQ---------QTDKFTG--QQSFLNHS------NHQERDT
AT5G57610.1_Raf35           FPPVHCAHCPPNRESFLLNTDPKPTHHGAYPNETFGP--DRGWMVQQQVNPNPPRIEEGR
AT1G08720.1_EDR1            RLP--CRLVKGSH-----------------------------YTGNE--DD-------A-
AT5G11850.1_Raf3            NLP--CMLVKGSY-----------------------------YTGTD--DG-------A-
AT1G18160.1_Raf4            GVP--CRIVKGQQ-----------------------------YTGSD--DV-------A-
AT1G73660.1                 GVP--CRIVKGQQ-----------------------------YTGSE--DV-------A-
AT4G24480.1_Raf6            GLP--CRIARGCR-----------------------------YCKES--HQ-------S-
AT5G03730.2_Raf1/CTR1       DLP--CRIAKGCK-----------------------------YCNRD--DA-------A-
AT4G23050.2_Raf12           GIR--NRVCRGET-----------------------------WTGQF--PF-------Q-
AT5G49470.2_Raf10           NIT--RRCSSGES-----------------------------WTGEF--PV-------K-
AT1G67890.1_Raf11           NIF--QRCSSGES-----------------------------WTGEF--PV-------K-
AT3G06620.1_Raf7            NIA--RRCVRGES-----------------------------WTGEF--PV-------K-
AT3G06630.1_Raf8            NVA--RRCANGES-----------------------------WTGEF--PV-------K-
AT3G06640.1_Raf9            TIA--QLCSNGES-----------------------------WTGKF--PV-------K-
AT1G14000.1_VIK             ------------------------------------------------------------
AT2G31800.1_ILK3            ------------------------------------------------------------
AT2G43850.1_ILK1            ------------------------------------------------------------
AT3G59830.1_ILK2            ------------------------------------------------------------
AT3G58760.1_ILK4            ------------------------------------------------------------
AT4G18950.1_Raf27           ------------------------------------------------------------
AT2G31010.1_Raf13           GLE--SRLVVGLP----------------------SDGTVNCMDSNKHMSV-------IV
AT3G58640.1_Raf15           GLQ--SRLVVGLP----------------------SDGAAESVDSYSHISV-------TV
AT5G01850.1_Raf31           ------------------------------------------------------------
AT5G50180.1_Raf34           ------------------------------------------------------------
AT3G27560.1                 ------------------------------------------------------------
AT5G40540.1_Raf32           ------------------------------------------------------------
AT5G66710.1_Raf37           ------------------------------------------------------------
AT3G50720.1_Raf44           ------------------------------------------------------------
AT3G50730.1_Raf45           ------------------------------------------------------------
AT3G63260.1_Raf48/MRK1      ------------------------------------------------------------
AT3G22750.1_Raf39           ------------------------------------------------------------
AT4G14780.1_Raf26           ------------------------------------------------------------
AT3G01490.1_Raf38           ------------------------------------------------------------
AT5G50000.1_Raf33           ------------------------------------------------------------
AT4G38470.1_Raf30           TMDRRERIKMEVFDEVLRRLRQ-----SDIEDAHLPGFEDDLWNHFNRLPA-------RY
AT2G17700.1_Raf21           ENPRHYRMKLDVYSEVLQRLQE-----SNYEEATLPDFEDQLWLHFNRLPA-------RY
AT4G35780.1_Raf29           ESPRQHRMKLEVYGEVLQRIQE-----SNYEEANFPGFDDLLWLHFNRLPA-------RY
AT2G24360.1_Raf22           ------------------------------------------------------------
AT4G31170.1_Raf28           ------------------------------------------------------------
AT1G62400.1_Raf19/HT1       ------------------------------------------------------------
AT3G46930.1_Raf43           -----------------------------------MDGEVTSWIRRANFSH-------TV
AT5G58950.1_Raf36           -----------------------------------MDEEATSWIRRAKFSQ-------TV


AT1G04700.1_Raf16           -----------------------------------------------------GSNS---
AT2G35050.1_Raf24           -----------------------------------------------------GMKKN--
AT1G16270.1_Raf18           -----------------------------------------------------VPVSG--
```

```
AT1G79570.1_Raf5          -----------------------------------------------------VPLSG--
AT3G46920.1_Raf42         LHNANLATA------------------------QVET-----TEPFVNEIVRDVPIKVQV
AT5G57610.1_Raf35         SHISNVGRPND--HYTPDYPV--SNYPLGQRAGHEIS-----NEGFHDKPLGGIPLNSAN
AT1G08720.1_EDR1          ---VNTIRLED----EREYLV-----DLMTDPGTLIP-----ADFASASNNTVEP-----
AT5G11850.1_Raf3          ---VNLIKLDD----KSEYII-----DLMGAPGALIP-----SEVPSSFLPVSCT-----
AT1G18160.1_Raf4          ---MNSIKTDD----GREYIV-----DLMGDPGTLIP-----ADAAGLQMDFDDS-----
AT1G73660.1               ---MNFIKADD----GREYIV-----DLMGDPGTLIP-----ADAAGLQIDYDES-----
AT4G24480.1_Raf6          ---SCLVKIDDDRKLSREYVV-----DLIGEPGNVHD-----PDSSI---NGETQ-----
AT5G03730.2_Raf1/CTR1     ---SCLVRFG----LDREYLV-----DLVGKPGHLWE-----PDSLL---NGPSS-----
AT4G23050.2_Raf12         ---KKT---------GELFMALVTKSPVYE-NGELVGVVTVSSDATLFNRMHPLSN----
AT5G49470.2_Raf10         ---NKA---------GERFSVVTTMSPSYDDDGCLIGIICITNDSALFQDPRGSPA---K
AT1G67890.1_Raf11         ---NKM---------GERFSVVTTISPFYDDDGLLIGIICITNDSALFQRPRVPPA---K
AT3G06620.1_Raf7          ---SKS---------GDRFSAVTTCSPFYDDDGALMGIICITSNTAPYLNPRISLA---K
AT3G06630.1_Raf8          ---TKS---------GKIFSAVTTCSPFYDDNGTVVGIISITSDIAPYLNPRLSLP---R
AT3G06640.1_Raf9          ---RRT---------GEKFSAVTTCSPFYADDGSLIGIVSITSDVAPYLNPTISLA---K
AT1G14000.1_VIK           ------------------------------------------------------------
AT2G31800.1_ILK3          ------------------------------------------------------------
AT2G43850.1_ILK1          ------------------------------------------------------------
AT3G59830.1_ILK2          ------------------------------------------------------------
AT3G58760.1_ILK4          ------------------------------------------------------------
AT4G18950.1_Raf27         ------------------------------------------------------------
AT2G31010.1_Raf13         V----L---NS--VE---LL-----VDLIRFPGQLVPR-----SAKAIFMSHISPA----
AT3G58640.1_Raf15         L----L---NS--VE---ML-----VDLMRFPGQLIPL-----STKAIFMSHISAA----
AT5G01850.1_Raf31         ------------------------------------------------------------
AT5G50180.1_Raf34         ------------------------------------------------------------
AT3G27560.1               ------------------------------------------------------------
AT5G40540.1_Raf32         ------------------------------------------------------------
AT5G66710.1_Raf37         ------------------------------------------------------------
AT3G50720.1_Raf44         ------------------------------------------------------------
AT3G50730.1_Raf45         ------------------------------------------------------------
AT3G63260.1_Raf48/MRK1    ------------------------------------------------------------
AT3G22750.1_Raf39         ------------------------------------------------------------
AT4G14780.1_Raf26         ------------------------------------------------------------
AT3G01490.1_Raf38         ------------------------------------------------------------
AT5G50000.1_Raf33         ------------------------------------------------------------
AT4G38470.1_Raf30         ALDVNVERAED--VLMHKRL-----LHSAYDPQ---NR-----PAIEVHLVQVQPA----
AT2G17700.1_Raf21         ALDVKVERAED--VLTHQRL-----LKLAADPA---TR-----PVFEVRSVQVSPR----
AT4G35780.1_Raf29         ALDVNVERAED--VLTHQRL-----LKLAEDPA---TR-----PVFEVRCVQVSPT----
AT2G24360.1_Raf22         -------------------------------ML---EG-----AKFNVLA--VGNH----
AT4G31170.1_Raf28         -------------------------------ML---EN-----PKFDLHA--VGNH----
AT1G62400.1_Raf19/HT1     ------------------------------------------------------------
AT3G46930.1_Raf43         CYRMITPSLES-----------------MPFTV---NQ-----EK---------------
AT5G58950.1_Raf36         SYRLNSSKLAS-----------------LPFMI---NQ-----DKFSGLK--AIPQ----


AT1G04700.1_Raf16         ----------NSSFSPNYFSP---------------------------------------
AT2G35050.1_Raf24         -------QDNQFCLLGGFSVS-GHG-----TS------------NNSSSNVSNFDQP---
AT1G16270.1_Raf18         -------EDDQLSTSSGTCGL---------VH------------TDSESNLIDLDYPEPL
AT1G79570.1_Raf5          -------IEDQLSTSSSTYAP---------VH------------SDSESNLIDLNYPEPE
AT3G46920.1_Raf42         TRQQQHPVDPSVAYAQCR------E-----L--SGLVDNVNIHAPEIYSNCQNFISPVDK
AT5G57610.1_Raf35         RSAE----ERGFHYGNNLYPP-GPD-----SIHSAGHSHMHHPQPNIWQNVSNPIAGPPG
AT1G08720.1_EDR1          ------------CNSNGNKFPTAQFSNDVPKLSEGEGS----------------------
AT5G11850.1_Raf3          ------------DT---RVFPENLDSLQHSSP--VLEK----------------------
AT1G18160.1_Raf4          ------------VY---SASPRDVDSSHVASSSSGVES----------------------
AT1G73660.1               ------------AY---SASPGDNDSIHVASSSNGIES----------------------
AT4G24480.1_Raf6          ------------CQ---IPSPLQ--MSHLTDFSRPCV-----------------------
AT5G03730.2_Raf1/CTR1     ------------IS---ISSPLR--FPRPKPVEPAVD-----------------------
AT4G23050.2_Raf12         -EHQQQARSN-----NRHESNLRKHQWHLPRPQIAAASQVPVVP---------QYS----
AT5G49470.2_Raf10         TRRGQEGETSFSRVTSSVASKLGLDSKEAVVSKLGLDSQQPIQV---------AIA----
AT1G67890.1_Raf11         NRWQE-GDSSFCRGTNGVASRLGFDSKEAVVSKLGLDSQQPIQA---------AIA----
AT3G06620.1_Raf7          LKAQEEGETSSIPARNSFASKLGLDSRGAVISKLGLDSDQPIQV---------AIA----
AT3G06630.1_Raf8          LKPQE-PE-----------RKLGLDSKGAVISKPGLDSDQPIQV---------SIA----
AT3G06640.1_Raf9          LKASE-VETSSTPARNSFAFKLGLDTKGAVVSKLGLDSDQPIQV---------AIA----
AT1G14000.1_VIK           ------------------------------------------------------------
AT2G31800.1_ILK3          ------------------------------------------------------------
AT2G43850.1_ILK1          ------------------------------------------------------------
AT3G59830.1_ILK2          ------------------------------------------------------------
AT3G58760.1_ILK4          ------------------------------------------------------------
```

```
AT4G18950.1_Raf27          ------------------------------------------------------------
AT2G31010.1_Raf13          ------GE---------------SDSAENDSCDSPLEPNSPL------------YER---
AT3G58640.1_Raf15          ------GE---------------SDSAENDSCDSPLEPNSPM------------FGYPEK
AT5G01850.1_Raf31          ------------------------------------------------------------
AT5G50180.1_Raf34          ------------------------------------------------------------
AT3G27560.1                ------------------------------------------------------------
AT5G40540.1_Raf32          ------------------------------------------------------------
AT5G66710.1_Raf37          ------------------------------------------------------------
AT3G50720.1_Raf44          ------------------------------------------------------------
AT3G50730.1_Raf45          ------------------------------------------------------------
AT3G63260.1_Raf48/MRK1     ------------------------------------------------------------
AT3G22750.1_Raf39          ------------------------------------------------------------
AT4G14780.1_Raf26          ------------------------------------------------------------
AT3G01490.1_Raf38          ------------------------------------------------------------
AT5G50000.1_Raf33          ------------------------------------------------------------
AT4G38470.1_Raf30          ------GI---------------SAD--LDSTS-----NDAGHS----SPTRKSIHPPPA
AT2G17700.1_Raf21          ------IS---------------ADS------DPAVEEDAQSSH----QPSGPGVLAPPT
AT4G35780.1_Raf29          ------LN---------------GNSGDVDPSDPAVNEDAQSSY-------NSRSLAPPT
AT2G24360.1_Raf22          ------------------------------------------HN----------------
AT4G31170.1_Raf28          -------------------------------------------N----------------
AT1G62400.1_Raf19/HT1      ------------------------------------------------------------
AT3G46930.1_Raf43          ------------------------------------------------------------
AT5G58950.1_Raf36          ------RS---------------SSSSSASSSDPKLVSSNSQTT----------------


AT1G04700.1_Raf16          -----------NHQPAAQI----TSSDSQD-----S-GSSVFSLSVNTNENYLDCS---R
AT2G35050.1_Raf24          VTQQRVF--HSERTVRDPT-ETNRLSKSDD-----S-LASQFVMAQTTSDAFLPI-----
AT1G16270.1_Raf18          QPTRRVY--RSERIPREQLEMLNRLSKSDD-----S-LGSQFLMSHPQASTGQQEP---A
AT1G79570.1_Raf5           QSSQRVY--CSERIPREQLELLNRLSKSDN-----S-LSSQFVTSESPANTAQQDS---G
AT3G46920.1_Raf42          IGKEDIMGTSSQQMARK---------------------------------NMFL------
AT5G57610.1_Raf35          LP-MQINGTVNQTVIRNPIETAPRYSTGM-----------------ENQ-GVLVGS---P
AT1G08720.1_EDR1           -----SHSSMAN-YSSSLD---RRTEAERTD--SS------------------------Y
AT5G11850.1_Raf3           -----EIETPAFSVSKEAD---S-----------RSGMVAN-FFTGNQEENS-DRCAVEK
AT1G18160.1_Raf4           -----SIEEHTESWSAEHR---SRTKGSREE--NQSAGGGD-LMIPN-------IREAVG
AT1G73660.1                -----SYEENTEFRTGEHR---SSTKSSGER--NQSGGGGD-LIVHPNISRE-DVKNQKK
AT4G24480.1_Raf6           ------HSTSPCQTVESKT---SRTL-SENI--QRSGSQGQ---VH--------------
AT5G03730.2_Raf1/CTR1      ------FRLLAKQYFSDSQ---SLNLVFDPA--SDDMGFSM---FH--------------
AT4G23050.2_Raf12          ----S---AVASNLKASKL---LPQRNGDDS---FNGN-HN---SRSRDENVPVVASTTF
AT5G49470.2_Raf10          ----SKISDL---VGNKVK---SKMRAGDNNAANLEGGSGD---SHQSDQGF--------
AT1G67890.1_Raf11          ----SKISDLASKVGNKVR---SKMRAGDNNASHPEGGNGG---SHQSDQGF--------
AT3G06620.1_Raf7           ----SKISDLASKVSNKVR---SKMRAGDNSATLSEGGSGD---SHQKDHNV--------
AT3G06630.1_Raf8           ----SKISSLASKLSNKVR---SKMRAGDNS------ACGD---SHHSDHDV--------
AT3G06640.1_Raf9           ----SKISDLASKVRNKVR---SKMPAGDSSVTVFEGETGD---SHHSDHGV--------
AT1G14000.1_VIK            ------------------------------------------------------------
AT2G31800.1_ILK3           ------MANVVGQLKRGI--------S-----RQFSTGSLRRTLSRQ-------------
AT2G43850.1_ILK1           ------MENITAQLKRGI--------S-----RQFSTGSIRRTLSRQ-------------
AT3G59830.1_ILK2           ------MDNIAAQLKRGI--------S-----RQFSTGSMRRTLSRQ-------------
AT3G58760.1_ILK4           ------------------------------------------------------------
AT4G18950.1_Raf27          ------------------------------------------------------------
AT2G31010.1_Raf13          RDPESTEKDENLQFYRKL--------EGY---PNASGSSLRSLMLRP-------------
AT3G58640.1_Raf15          FDHENAEKDENLSLHRKL--------DGS---PNTSGPPSRNMLLRS-------------
AT5G01850.1_Raf31          ------------------------------------------------------------
AT5G50180.1_Raf34          ------------------------------------------------------------
AT3G27560.1                ------------------------------------------------------------
AT5G40540.1_Raf32          ------------------------------------------------------------
AT5G66710.1_Raf37          ------------------------------------------------------------
AT3G50720.1_Raf44          ------------------------------------------------------------
AT3G50730.1_Raf45          ------------------------------------------------------------
AT3G63260.1_Raf48/MRK1     ------------------------------------------------------------
AT3G22750.1_Raf39          ------------------------------------------------------------
AT4G14780.1_Raf26          ------------------------------------------------------------
AT3G01490.1_Raf38          ------------------------------------------------------------
AT5G50000.1_Raf33          ------------------------------------------------------------
AT4G38470.1_Raf30          FGSSPNLEALALAASLSQ--------D---------------------------------
AT2G17700.1_Raf21          FGSSPNFEAITQGSKIV-------------------------------------------
AT4G35780.1_Raf29          FGSSPNFEALTQAYKDH---------A---------------------------------
AT2G24360.1_Raf22          -N-DNNYYAFTQEFYQKL--------N---------------------------------
AT4G31170.1_Raf28          -N-DNNYYAFTQDFYQKL--------G---------------------------------
```

```
AT1G62400.1_Raf19/HT1        ------------------------------------------------------------
AT3G46930.1_Raf43            ------------------------------------------------------------
AT5G58950.1_Raf36            -G-DTSSL-EAADVYVVD--------S---------------------------------


AT1G04700.1_Raf16            EKFNGFQHDMSLDILI---------RSHT-----------SATDQLCS---------TTK
AT2G35050.1_Raf24            SESSETSHEANME----------SQNVHPTAPV------IPAPDSIWTAEG------SMS
AT1G16270.1_Raf18            KEAAGISHEDSHIVND-------VE---------NISGNVVA---------------SNE
AT1G79570.1_Raf5             KEAVGKSHDEFKTVND-------DANHHTHKDVETIFEKVGVSDETLESEPLHKIVNPDD
AT3G46920.1_Raf42            -------HDTSGQSPV-------SPNIDHTD----------------SAKRLTRVVLPGH
AT5G57610.1_Raf35            QRISGFDGMSSLGQPS-------YPNPHLQD----------------RAFPLD-------
AT1G08720.1_EDR1             PKVGPLRNID-YSSPSSVTSSTQLENNS-STAIGKG------------------------
AT5G11850.1_Raf3             HQTERFEHDF--GKL---MHSQQISGEN-MPPFS--GKP-TCAQK----------VKV--
AT1G18160.1_Raf4             SQKAPVQHLS--SKP---THSF--THAR-SPSWTEGVSS-PAGRR----------MKV--
AT1G73660.1                  VEKAPFQNLS--SRP---IHSF--THMR-SPSWTEGVSS-PAAQR----------MKV--
AT4G24480.1_Raf6             --------KE-FELP----------DNA-GTVCCAHIDQ-TCC-----------------
AT5G03730.2_Raf1/CTR1        --------RQ-YDNP----------GGE-NDALA--------------------------
AT4G23050.2_Raf12            EKYGSLADKF-LGKLQRKITGSQGTEDN-EPILRNGINKSACGSG----------GSSKA
AT5G49470.2_Raf10            -----FDAAF-ADRREDAATSGADT-PR-GDFIQSPFGVFLRSDE---------------
AT1G67890.1_Raf11            -----FDAAF-SDQREDAETNDAST-PR-GNLIQSPFGVFLCNDD---------------
AT3G06620.1_Raf7             -----FGATL-VDHRDDAASSGAST-PR-GDFIQSPFGVFTCNDE---------------
AT3G06630.1_Raf8             -----FGDTL-SDHRDDAASSGAST-PR-GDFIQSPFGVFTCYDD---------------
AT3G06640.1_Raf9             -----FGATL-SDHMDDAASSGAST-PR-GDFIQSPFGVFTCNDD---------------
AT1G14000.1_VIK              --------------------MSSDSPAA-GDGGEQAAA-----------G-T--------
AT2G31800.1_ILK3             -----FTR-Q-------ASHDPRRNNMR-FSFGRQSSL-----------DPI--------
AT2G43850.1_ILK1             -----FTR-Q-------SSLDPRRTNMR-FSFGRQSSL-----------DPI--------
AT3G59830.1_ILK2             -----FTR-Q-------NSLDPRRNNMR-FSFGRQSSL-----------DPI--------
AT3G58760.1_ILK4             ------------------MTIKPKSPAR-FKLGRQSSL-----------APE--------
AT4G18950.1_Raf27            -------------------MEEDYQQPR-FTIGRQSSM-----------APE--------
AT2G31010.1_Raf13            --STAIERKL-------SNTSHSEPNVA-TVFWRRSRRKVIAEQRTASSSPEHPSMRR-G
AT3G58640.1_Raf15            --ASALERKL-------SF-SQSESNMA-NEFWRQSRRKVIADQRTASSSPEHLSFRART
AT5G01850.1_Raf31            ------------------------------------------------------------
AT5G50180.1_Raf34            ------------------------------------------------------------
AT3G27560.1                  ------------------------------------------------------------
AT5G40540.1_Raf32            ------------------------------------------------------------
AT5G66710.1_Raf37            -------------------------------------------------------MRP--
AT3G50720.1_Raf44            ------------------------------------------------------------
AT3G50730.1_Raf45            ------------------------------------------------------------
AT3G63260.1_Raf48/MRK1       -------------------------------------------------------MAS--
AT3G22750.1_Raf39            -------------------------------------------------------MET--
AT4G14780.1_Raf26            ------------------------------------------------------------
AT3G01490.1_Raf38            ------------------------------------------------------------
AT5G50000.1_Raf33            ------------------------------------------------------------
AT4G38470.1_Raf30            ---------------------EDADN--------------SVHNNSLYSRPLHE------
AT2G17700.1_Raf21            ---------------------EDVDS--------------VVNA-TLSTRPMHE------
AT4G35780.1_Raf29            ---------------------QDDDS--------------AVNAQLPNSRPMHE------
AT2G24360.1_Raf22            ----------------------EGSNMS-ME-------SMQTSNAGGSV----S-MSV--
AT4G31170.1_Raf28            ---------------------EEGTNMS-VD-------SMQTSNAGGSV----S-MSV--
AT1G62400.1_Raf19/HT1        ------------------------------------------------------------
AT3G46930.1_Raf43            ----------------------MQRN--------------PVTNKKRSVSPLPH-MAL--
AT5G58950.1_Raf36            ---------------------EIQTN--------------PVTHKQRSVSPSPQ-MAV--


AT1G04700.1_Raf16            SSD----------------------------------------KADYSSPNTNFPVVFLR
AT2G35050.1_Raf24            QSE----KK-------NVETNTPEHVSQTETSAKAVPQGHNEKGDIVVDINDRFPREFLA
AT1G16270.1_Raf18            TLD----KRTVSGGGIETEARNLSHVD-TERSHDIPEKQTS--SGVLIDINDRFPQDFLS
AT1G79570.1_Raf5             ANK----NRVVNGADTEIGVSNLSHVN-AAMSHVIPEEQASLQGDILIDINDRFPRDFLS
AT3G46920.1_Raf42            ESQ----PK---------------------ES--CVPTQSPLLG---------NPGLYLQ
AT5G57610.1_Raf35            ------------------------------PN--WVPSENPTVH---------NEHLQVR
AT1G08720.1_EDR1             ------SRGAIIECS------------------RTNMNIV-----PYNQNSEEDPKNLFA
AT5G11850.1_Raf3             --K--NVSKYVISAA-------------------KNPEFAQKLHAVLLESGASPPPDLFM
AT1G18160.1_Raf4             --K--DVSQYMIDAA------------------KENPQLAQKLHDVLLESGVVAPRNLFS
AT1G73660.1                  --K--DVSQYMIDAA------------------KENPRLAQKLHDVLLESGVVAPPNLFS
AT4G24480.1_Raf6             --------------A------------------K-------VSSMVLTESVLRALPLDIP
AT5G03730.2_Raf1/CTR1        ------------------------------------------------ENGGGSLPP---
AT4G23050.2_Raf12            SNA--VTCTAFRDN------------------GNGKPKRA---------------EVRIS
AT5G49470.2_Raf10            --K--ASTKPFRDSSDE---------------SDGNSVVP---------------KT---
AT1G67890.1_Raf11            --K--SSSKASGESNDE---------------NDRNSVVP---------------KK---
```

```
AT3G06620.1_Raf7          --K--FVSKPFKDSSDE---------------SDGKPAIH---------------KV---
AT3G06630.1_Raf8          --K--FPSKPSKDS------------------SDRKPAIH---------------KV---
AT3G06640.1_Raf9          --K--FSSEPFIDS------------------SDGYPIT-----------------L---
AT1G14000.1_VIK           ---------------------------------------SVPSPSYD---------K---
AT2G31800.1_ILK3          ---------------------------------------RRSPDGS---NGP----Q---
AT2G43850.1_ILK1          ---------------------------------------RRSPDSSKSDDEP----H---
AT3G59830.1_ILK2          ---------------------------------------RRSPESLS--CEP----H---
AT3G58760.1_ILK4          ---------------------------------------SRTPIDTLTEDEDDDLAA---
AT4G18950.1_Raf27         ---------------------------------------KIPEPSVHSEEEV--------
AT2G31010.1_Raf13         RSMLSTGRNSFRDYTGEASSPSSSS-------TSEIRKTRRRSFRITPEIG---------
AT3G58640.1_Raf15         KSMLSGDKNLARDFTGDVATSSCKSVG---GAKMETKRIRRRSISITPEIG---------
AT5G01850.1_Raf31         ------------------------------------------------------------
AT5G50180.1_Raf34         ------------------------------------------------------------
AT3G27560.1               ------------------------------------------------------------
AT5G40540.1_Raf32         ------------------------------------------------------------
AT5G66710.1_Raf37         ------R--GYQRA-----------------PSM------QKPTDYPTDKT---------
AT3G50720.1_Raf44         -------------------------------------------MAISPTMM---------
AT3G50730.1_Raf45         ----------------------------------------------MISRM---------
AT3G63260.1_Raf48/MRK1    -----GG--G--EADKSLEIG----------SGT-------ADPKIGGTGS---------
AT3G22750.1_Raf39         -----RN---------E-TKA----------SPE-------NNLRNRGADG---------
AT4G14780.1_Raf26         ------------------------------------------MEKKSEEDG---------
AT3G01490.1_Raf38         ----------------------------------------------MKEKA---------
AT5G50000.1_Raf33         ------------------------------------------------------------
AT4G38470.1_Raf30         ------------------ITF----------STEDKPKLLFQLTALLAELG---------
AT2G17700.1_Raf21         ------------------ITF----------STIDKPKLLSQLTSLLGELG---------
AT4G35780.1_Raf29         ------------------ITF----------STIDRPKLLSQLTSMLGELG---------
AT2G24360.1_Raf22         -----DN--S---------------------SVG-------SSDALIGHPG---------
AT4G31170.1_Raf28         -----DN--S---------------------SVG-------SSDALIGHPG---------
AT1G62400.1_Raf19/HT1     ------------------------------------------------------------
AT3G46930.1_Raf43         -----SD--AFIEAKSDIKRF----------STP-------HPRRVEPEKG---------
AT5G58950.1_Raf36         -----PD--VFKEARSERKRF----------STP-------HPRRVESEKG---------


AT1G04700.1_Raf16         QEPMIPRHDL-ETNSDDSDTQKSLPREESIHYSG--LP--LRKVGSR-------ETTFMH
AT2G35050.1_Raf24         DILKTKE-------SLNF------PGLGPLHADGAGVSLNIQNNDPK-------TWSYFR
AT1G16270.1_Raf18         EIFAKAL-------SDDM-----PSGANPYQHDGAGVSLNVENHDPK-------NWSYFR
AT1G79570.1_Raf5          EIFSQAI-------SEDT------STVRPYPHDGAAVSMNVQNHDRK-------NWSYFQ
AT3G46920.1_Raf42         SLVGGQQFDSAEAQSSNP-----AYDVVESTFDAANLPSSLSSNPDA--ANLPSSLSS--
AT5G57610.1_Raf35         EPLPGPLLQ-----------------------------TNLTAAPIMQTPVMQTSVESKL
AT1G08720.1_EDR1          DLNPFQNKGADKLYMPTK---SGLNNVDDFH--------QQKNNPLVGRSPAPMMWKNYS
AT5G11850.1_Raf3          DINPHNLRGKNLLQEL--------------------R-QESSNSMVSGIPCYPEKVAE--
AT1G18160.1_Raf4          EVYSESMEATGEIKSVAE---SNDEKGKDFGTIQQGR-NQSNLGPVRFLPPLPRPQSKAI
AT1G73660.1               EVYPQQLEATVESKNSTE---AKKERGKDLETTQEGR-HQNGFGPVRFLPPLPRVQSKTN
AT4G24480.1_Raf6          NLSEEKIAPQETCK----------------------------EETVLLEDPTAMKQP---
AT5G03730.2_Raf1/CTR1     ---SANMPPQNMMR----------------------------ASNQIEA--APMNAP---
AT4G23050.2_Raf12         DVYGNG-----A-----E---GLIHNGDRFQYI--GN-LGQS------------------
AT5G49470.2_Raf10         -LTSKA-----E-----E---WMVKKGLSWPWK--GN-EREGL----EGRRSHSVWPWVR
AT1G67890.1_Raf11         -LTSKT-----E-----E---WMVKKGLSWPWK--GN-EREGL----ERRNAHSVWPWVH
AT3G06620.1_Raf7          -LTSKA-----E-----E---WMVKKGLSWPWK--GN-EQEGS----KGRPTNSVWPWVQ
AT3G06630.1_Raf8          -PTSKA-----E-----E---WMVKKGLSRPWK--GN-EQEGS----RVRPTHSVWSWVE
AT3G06640.1_Raf9          -FTSKA-----E-----E---WMVKKGLSWPWK--GN-EQEGS----RVKPTYSVWPCVQ
AT1G14000.1_VIK           -Q-KEKARVSRTSLI-------------LWHAH---------QNDAAAVRKLLEEDP---
AT2G31800.1_ILK3          -L-AVPDNLDATMQL-------------LFVAC---------RGDVEGVQDLLDEG----
AT2G43850.1_ILK1          -M-SVPENLDSTMQL-------------LFMAS---------KGDVRGIEELLDEG----
AT3G59830.1_ILK2          -M-SVPENLDSTMQL-------------LFMAS---------KGDVNGVEELLNEG----
AT3G58760.1_ILK4          -A-ATAGIGDPTIRL-------------MYLAN---------EGDIDGINKMLDSG----
AT4G18950.1_Raf27         -F-EDGEEIDGGVRL-------------MYLAN---------EGDIEGIKELIDSG----
AT2G31010.1_Raf13         ------DDIASAVRE-------------MYEKSKQNR-LLQGREDEN-------------
AT3G58640.1_Raf15         ------DDIVRAVRA-------------MNEALKQNR-LSKEQGDDDSSPNSPNDR----
AT5G01850.1_Raf31         ------------------------------------------------------------
AT5G50180.1_Raf34         ------------------------------------------------------------
AT3G27560.1               ------------------------------------------------------------
AT5G40540.1_Raf32         ------------------------------------------------------------
AT5G66710.1_Raf37         ------LHPNYP-----------------F----------------------LMSS----
AT3G50720.1_Raf44         ------LNANYP-----------------F----------------------FMSA----
AT3G50730.1_Raf45         ------IFRNY-------------------------------------------------
AT3G63260.1_Raf48/MRK1    ------RSAGEE----------------RYF---------------------RADT----
AT3G22750.1_Raf39         ------NNSKKD----------------MIF---------------------RADK----
```

```
AT4G14780.1_Raf26          ------NNTTKE----------------KIF---------------------RADK----
AT3G01490.1_Raf38          ------ESGGGV----------------GYV---------------------RADQ----
AT5G50000.1_Raf33          ------MKEGKD----------------GFV---------------------RADQ----
AT4G38470.1_Raf30          ------LNIQEA----------------HAF--------------------STTDG----
AT2G17700.1_Raf21          ------LNIQEA----------------HAF--------------------STVDG----
AT4G35780.1_Raf29          ------LNIQEA----------------HAF--------------------STADG----
AT2G24360.1_Raf22          ------LKPVRH-----------------YS-----------------------LS----
AT4G31170.1_Raf28          ------LKPMRH----------------PYS-----------------------LS----
AT1G62400.1_Raf19/HT1      ------------------------------------------------------------
AT3G46930.1_Raf43          ------MKAKSS----------------SRK-----------------------DS----
AT5G58950.1_Raf36          ------MKPKLS----------------HK------------------------NS----


AT1G04700.1_Raf16          TQGSDDFFKSKLLGPQ--------------------------------------------
AT2G35050.1_Raf24          NLAQDEFERKDLSLMDQ-------------DH--------------------PGFPTSMT
AT1G16270.1_Raf18          NLADEQFSDRDVAYID--------------RT--------------------PGFPSDME
AT1G79570.1_Raf5           QLAEDQFIQRDVVLDQ--------------AD--------------------SRIPSDRK
AT3G46920.1_Raf42          SVGGADHKESSKSLFSN-------------QDPWNL-----------QTNSNEDVKPDLL
AT5G57610.1_Raf35          AQGGEQF-NYVNTGISN-------------GVPYQD-----------KPQPLAGGKKDMG
AT1G08720.1_EDR1           CNEAPKRKENSY--IENLL------PK-L----HRD-PRYGNTQSSYATSSSNGAISSNV
AT5G11850.1_Raf3           -----QLRE----------------------------SERNPTAESYQQSVEV-------
AT1G18160.1_Raf4           TH---DLREHSGSGLGHLS------EH-CNIDGHSDSSHS-ETSTDYPRNVPV-------
AT1G73660.1                AH---DQRDNGKV------------------VSQSDSSHSEASSTEYARTVPA-------
AT4G24480.1_Raf6           ------------------------------------------------------------
AT5G03730.2_Raf1/CTR1      ------------------------------------------------------------
AT4G23050.2_Raf12          -------------------------------------------------KPPRGLESGLV
AT5G49470.2_Raf10          NE---QQKQQ-----------------------------------AYQSNSNHSVKSESQ
AT1G67890.1_Raf11          NE---QQKEE-----------------------------------AHHSNSYNSVKSESL
AT3G06620.1_Raf7           NE---QKKER-----------------------------------CHQINPSAGVQYESH
AT3G06630.1_Raf8           NE---QEKDK-----------------------------------YHQIYPSAGVKSESH
AT3G06640.1_Raf9           NE---QKKDK-----------------------------------SHQINRYSGVKSKSH
AT1G14000.1_VIK            ------------TLVHARDYDKRTPLHVASLHGWID-----------------VVKCLLE
AT2G31800.1_ILK3           ------------IDVNSIDLDGRTALHIAACEGHVD-----------------VVKLLLT
AT2G43850.1_ILK1           ------------IDVNSIDLDGRTALHIAACEGHLG-----------------VVKALLS
AT3G59830.1_ILK2           ------------IDVNSIDLDGRTALHIASCEGHYD-----------------VVKVLLS
AT3G58760.1_ILK4           ------------TNVDYRDIDARTALHVAACQGRTD-----------------VVELLLS
AT4G18950.1_Raf27          ------------IDANYRDIDDRTALHVAACQGLKD-----------------VVELLLD
AT2G31010.1_Raf13          --------------SSVID-------N--NVSGLHLDD----------------------
AT3G58640.1_Raf15          ------------TESSHLQ-------K--NVSGFHLDAHD----------QVSGGRSTLS
AT5G01850.1_Raf31          ------------------------------------------------------------
AT5G50180.1_Raf34          ------------------------------------------------------------
AT3G27560.1                ------------MG----------------------------------------------
AT5G40540.1_Raf32          ------------MG----------------------------------------------
AT5G66710.1_Raf37          ------------HGLKSF------------------------------------------
AT3G50720.1_Raf44          ------------FGS---------------------------------------------
AT3G50730.1_Raf45          ------------------------------------------------------------
AT3G63260.1_Raf48/MRK1     ------------LDFSKWDL--HMGQT--STSSVL-------------------------
AT3G22750.1_Raf39          ------------IDLKNLDI--QLEKH--LSRVWSRSIEK--------------------
AT4G14780.1_Raf26          ------------IDLKSLDR--QLEKH--LSRVWSRNLEV--------------------
AT3G01490.1_Raf38          ------------IDLKSLDE--QLQRH--LSKAWTMEKRK----------SLSDGEDNVN
AT5G50000.1_Raf33          ------------IDLKSLDE--QLERH--LSRALTLEKNK----------KKDEEDTTA-
AT4G38470.1_Raf30          ------------YSLDVF-----------VVDGWPYEETE----------RLRIS---LE
AT2G17700.1_Raf21          ------------FSLDVF-----------VVDGWSQEETD----------GLRDA---LS
AT4G35780.1_Raf29          ------------FSLDVF-----------VVDGWSQEETE----------GLKDA---LK
AT2G24360.1_Raf22          ------------VGQSVF-----------R--------PG----------R--------V
AT4G31170.1_Raf28          ------------DGQSVF-----------R--------PG----------K--------V
AT1G62400.1_Raf19/HT1      ------------------------------------------------------------
AT3G46930.1_Raf43          ------------SEKKSV-----------NLRSLSH--SG----------PIRDLSTQKV
AT5G58950.1_Raf36          ------------FDKRSF-----------NLR--SP--SG----------PIRDLGTLRI


AT1G04700.1_Raf16          ----LIVEDVTNEVISDNLLSATIVPQVNRE----SDDDH------K-------------
AT2G35050.1_Raf24          NTNGVPIDYSYPPLQSEKVASSQIHPQIHFD----GNIKP------DVSTI---------
AT1G16270.1_Raf18          DGGEIARLHQVAPLTENRV-----------------------------------------
AT1G79570.1_Raf5           DGGESSRLPYVSPLSRDGISTNLANPQLTLG----QDYGG------NFSEKDGGGT----
AT3G46920.1_Raf42          -------NSSKVILENDLL----IGLWFSLKGSEEEHIKQ------ELQNVAEGVAASVL
AT5G57610.1_Raf35          NL--VEVNPSAATLEGAEL----SVERLSFLPELMESVKR---------AALEGAAEVKA
AT1G08720.1_EDR1           HGRD-----NVTFVSPVAVPSSFTSTENQFRPSIVEDMNRNTNNELDLQPHTAAVVHGQQ
```

```
AT5G11850.1_Raf3          ------------------------------------------------------------
AT1G18160.1_Raf4          -----------AVAAAAVVASSMVVAA-------AKSA-NSDSSTLELSAAA-----AAA
AT1G73660.1               -----------AVAAAAVVASSMVAAA------AAKSA-NSDSSPIELPAAAAATATAAA
AT4G24480.1_Raf6          ------------------------------------------------------------
AT5G03730.2_Raf1/CTR1     ------------------------------------------------------------
AT4G23050.2_Raf12         SGM------------------------------RGTKM----------------------
AT5G49470.2_Raf10         ---------------------------------ACESI----------------------
AT1G67890.1_Raf11         ---------------------------------ASESN----------------------
AT3G06620.1_Raf7          ---------------------------------AFESN----------------------
AT3G06630.1_Raf8          ---------------------------------GSESN----------------------
AT3G06640.1_Raf9          ---------------------------------ASESN----------------------
AT1G14000.1_VIK           FGA--------DVN-------------------AQD---R--------------------
AT2G31800.1_ILK3          RKA--------NID-------------------ARD---R--------------------
AT2G43850.1_ILK1          RRA--------NID-------------------ARD---R--------------------
AT3G59830.1_ILK2          RRA--------NID-------------------ARD---R--------------------
AT3G58760.1_ILK4          RGA--------KVD-------------------TKD---R--------------------
AT4G18950.1_Raf27         RKA--------EVD-------------------PKD---R--------------------
AT2G31010.1_Raf13         --E-------LNSKKTMSLPSS----PHAYRCQTFG---R--------------------
AT3G58640.1_Raf15         REP-------LDPQKAISLPSS----PQNYRSQ-YE---Q--------------------
AT5G01850.1_Raf31         ------------------------------------------------------------
AT5G50180.1_Raf34         ------------------------------------------------------------
AT3G27560.1               ------------------------------------------------------------
AT5G40540.1_Raf32         ------------------------------------------------------------
AT5G66710.1_Raf37         ------------------------------------------------------------
AT3G50720.1_Raf44         ------------------------------------------------------------
AT3G50730.1_Raf45         ------------------------------------------------------------
AT3G63260.1_Raf48/MRK1    ------------------------------------------------------------
AT3G22750.1_Raf39         ------------------------------------------------------------
AT4G14780.1_Raf26         ------------------------------------------------------------
AT3G01490.1_Raf38         NTR-------HNQN-------------------NFG------------------------
AT5G50000.1_Raf33         -------------V-------------------AIG------------------------
AT4G38470.1_Raf30         KEA----------A-------------------KIE------------------------
AT2G17700.1_Raf21         KEI----------L-------------------KLK------------------------
AT4G35780.1_Raf29         KEI----------R-------------------KFK------------------------
AT2G24360.1_Raf22         THA----------L---------------------N------------------------
AT4G31170.1_Raf28         THA----------L---------------------N------------------------
AT1G62400.1_Raf19/HT1     MEK----------K-------------------RFD------------------------
AT3G46930.1_Raf43         KER----------G-------------------KSK------------------------
AT5G58950.1_Raf36         QER----------V-------------------KSK------------------------


AT1G04700.1_Raf16         --SY-----TREKEITNADHESE-----MEEKYKKS------------------RNTDDS
AT2G35050.1_Raf24         --TI------PDLNTVD-----------TQEDYSQSQIK------G---AESTDATLNAG
AT1G16270.1_Raf18         ---------DPQMKVTESEEFDA-----MVENLRTSDCE------Q---EDEKSETRNAG
AT1G79570.1_Raf5          -GSIPPALENEQMKVTESEEFGA-----MVENLRTPDSE------P---KDEKTETRHAA
AT3G46920.1_Raf42         QSST-PSYHEPPIKVDE-YAFNS-----KGEVSRNDEMK------Q--QSTHFKDIRNQL
AT5G57610.1_Raf35         HPEEAKDQVRPELVENESEHMNA-----QDEPEI-DSDS------D--NPNNFK------
AT1G08720.1_EDR1          NDESH-IHDHRKYTSDDIS------TGCDPRL-----KDHESTSSS--LDSTSYRNDPQV
AT5G11850.1_Raf3          ----D-LSMKRNFDLDNTG--KASSSENME-------VGTADGESA--VCD----SHDQG
AT1G18160.1_Raf4          VMATA-AAVSRQFELDSLSNGDAGSGGL---------HGVDSGGER--ISDRSIGNESSK
AT1G73660.1               VVATA-AAVSRQLELGSNSDGDDGSGGHEPQGSGDSNHGPNSGGER--ISDKSIGNESSK
AT4G24480.1_Raf6          ----N-LSVEPEIVEADTRKDK---KGRLP-------VD------A--ISP--YLTIE--
AT5G03730.2_Raf1/CTR1     ----P-IS------------------------------Q------P--VPN--RANRE--
AT4G23050.2_Raf12         ---------------------SD--LNGEIEDAWNTRLSVDPLPILGVNSGRQQSPVNQR
AT5G49470.2_Raf10         ---------------------KA--SSNEPMGYWSSSVNVNSTSSS-SSCGSTSSS---V
AT1G67890.1_Raf11         ---------------------KP--ANNENMG----SVNVNSASSA-SSCGSTSSS---V
AT3G06620.1_Raf7          ---------------------KP--INNEASSLWSSPINANSTSSA-SSCGSTSSS---V
AT3G06630.1_Raf8          ---------------------KP--TDDEASNMWSSSINANSTNSA-SSCGSTSRS---V
AT3G06640.1_Raf9          ---------------------KP--TNNKASGLRSSCINANSA----ISRGIISHS---T
AT1G14000.1_VIK           WKNTP-LADA---------------EGARKQKMIELLKS------HGGLSYGQNGSHFEP
AT2G31800.1_ILK3          WGSTA-AADA---------------KYYGNMDVFNILKA------RGAKVPKTKR---TP
AT2G43850.1_ILK1          WGSTA-AADA---------------KYYGNLDVYNLLKA------RGAKVPKTRK---TP
AT3G59830.1_ILK2          WGSTA-AVDA---------------KYYGNVEVYNLLKA------RGAKAPKTRK---TP
AT3G58760.1_ILK4          WGSTP-LADA---------------VYYKNHDVIKLLEK------HGAKPT---I---AP
AT4G18950.1_Raf27         WGSTP-FADA---------------IFYKNIDVIKILEI------HGAKHP---M---AP
AT2G31010.1_Raf13         RGPSE-F---------------------AVKDTWNKVV-------------------ESS
AT3G58640.1_Raf15         SGSSH-R---------------------NISHIWDKVL-------------------GSP
AT5G01850.1_Raf31         ------------------------------------------------------------
```

```
AT5G50180.1_Raf34          ------------------------------------------------------------
AT3G27560.1                --------------------------------------------------------SASG
AT5G40540.1_Raf32          --------------------------------------------------------SVTG
AT5G66710.1_Raf37          -------------------------------------------------ESDDEDDDSDS
AT3G50720.1_Raf44          ------------------------------------------------------DDNNDE
AT3G50730.1_Raf45          ------------------------------------------------------PSHNES
AT3G63260.1_Raf48/MRK1     ---------------------------------------------------------TNS
AT3G22750.1_Raf39          ------------------------------------------------------------
AT4G14780.1_Raf26          ------------------------------------------------------------
AT3G01490.1_Raf38          ----------------------------HRQLVFQRPLL------GGGYSNNNNSSKNDI
AT5G50000.1_Raf33          ----------------------------GSASSSPVTLN------GGGFVG---------
AT4G38470.1_Raf30          ----------------------------LQSQSWPMQQS--------FSPEKENGQTGAR
AT2G17700.1_Raf21          ----------------------------DQPGSKQKSIS--------FFEHDKSSNELIP
AT4G35780.1_Raf29          ----------------------------DQPCSKQKSIT--------FFEHDKSTNELLP
AT2G24360.1_Raf22          -----------------------------DDALAQALMD--------TR-----------
AT4G31170.1_Raf28          -----------------------------DDALAQALMD--------SK-----------
AT1G62400.1_Raf19/HT1      -----------------------------SMESWSMILE--------SE------NVE--
AT3G46930.1_Raf43          -----------------------------IDKKSSKSVD--------YR--------G--
AT5G58950.1_Raf36          -----------------------------KDTGWSKLFD--------NT--------G--

                                                           GxGxxG
AT1G04700.1_Raf16          FSEA-AMVEIEAGIYGLQIIKNTDL--EDLHELGSGTFGTVYYGKWRGTD-VAIKRIKNS
AT2G35050.1_Raf24          VPLI-DFMAADSGMRSLQVIKNDDL--EELKELGSGTFGTVYHGKWRGTD-VAIKRIKRS
AT1G16270.1_Raf18          LPPV-GPSLADYDTSGLQIIMNDDL--EELKELGSGTFGTVYHGKWRGSD-VAIKRIKKS
AT1G79570.1_Raf5           LPPL-GS---EFDYSGLQIIKNEDL--EELRELGSGTFGTVYHGKWRGSD-VAIKRIKKS
AT3G46920.1_Raf42          LERLNFGYSGSDSLDQLQIIKDSDL--EELRELGSGTFGTVYHGKWRGTD-VAIKRINDR
AT5G57610.1_Raf35          IEQ--TKAEAEAKSRGLQSIRNDDL--EEIRELGHGTYGSVYHGKWKGSD-VAIKRIKAS
AT1G08720.1_EDR1           L---------DDADVGECEIPWNDLVIAE--RIGLGSYGEVYHADWHGTE-VAVKKFLDQ
AT5G11850.1_Raf3           IN----P---LLGEAAKWEIMWEDLQIGE--RIGIGSYGEVYRAEWNGTE-VAVKKFLDQ
AT1G18160.1_Raf4           SD----A---AIDDVAECEILWEEITVAE--RIGLGSYGEVYRGDWHGTA-VAVKKFIDQ
AT1G73660.1                SD---------CDDVSDCEILWEEITVGE--RIGLGSYGEVYRGDWHGTE-VAVKKFLDQ
AT4G24480.1_Raf6           ----------PSLASDWLEVSWNELHIKE--RVGAGSFGTVHRAEWHGSD-VAVKILSIQ
AT5G03730.2_Raf1/CTR1      ----------LGLDGDDMDIPWCDLNIKE--KIGAGSFGTVHRAEWHGSD-VAVKILMEQ
AT4G23050.2_Raf12          NN----RLV-TDS---SCEIRWEDLQLGE--EVGRGSFAAVHRGVWNGSD-VAIKVYFDG
AT5G49470.2_Raf10          MN----KVD-MDSDCLDYEILWEDLTIGE--QIGQGSCGTVYHGLWFGSD-VAVKVFSKQ
AT1G67890.1_Raf11          MN----KVD-MDSDCLDYEILWEDLTIGE--QIGQGSCGTVYHGLWFGSD-VAVKVFSKQ
AT3G06620.1_Raf7           MN----KVD-TDSEGLEYEILWDDLTIGE--QVGQGSCGTVYHGLWFGSD-VAVKVFSKQ
AT3G06630.1_Raf8           MD----KVD-IDSDPLEHEILWDDLTIGE--QIGRGSCGTVYHGIWFGSD-VAVKVFSKQ
AT3G06640.1_Raf9           MN----KVD-TNSNCLEYEILWDDLTIGE--QIGQGSCGTVYHGLWFGSD-VAVKLISKQ
AT1G14000.1_VIK            KP----VPP-PIPKKCDWEIEPAELDFSNAAMIGKGSFGEIVKAYWRGTP-VAVKRILPS
AT2G31800.1_ILK3           MV----VAN--PREVPEYELNPQELQVRKADGISKGIYQ---VAKWNGTK-VSVKILDKD
AT2G43850.1_ILK1           MT----VSN--PREVPEYELNPLEVQVRKSDGISKGAYQ---VAKWNGTR-VSVKILDKD
AT3G59830.1_ILK2           MT----VGN--PKEVPEYELNPLELQVRKVDGISKGTYQ---VAKWNGTR-VSVKIFDKD
AT3G58760.1_ILK4           MH----VLT--DKEVPEYEIHPTELDFSNSVKISKGTFN---KASWRGID-VAVKTFGEE
AT4G18950.1_Raf27          MH----VKT--AREVPEYEINPSELDFTQSKEITKGTYC---MAMWRGIQ-VAVKKLDDE
AT2G31010.1_Raf13          TL----QNQ-PLLPYQEWDIDFSELTVGT--RVGIGFFGEVFRGVWNGTD-VAIKLFLEQ
AT3G58640.1_Raf15          MF----QNK-PLLPYEEWNIDFSELTVGT--RVGIGFFGEVFRGIWNGTD-VAIKVFLEQ
AT5G01850.1_Raf31          ------MSS-DDTIEESLLVDPKLLFIGS--KIGEGAHGKVYQGRYGRQI-VAIKVVNRG
AT5G50180.1_Raf34          MD----SLT-GFRMEPKWQIDPQLLFVGP--KIGEGAHAKVYEGKYKNQT-VAIKIVHRG
AT3G27560.1                FY----SNE-EFELDPKWLVDPRHLFVGP--KIGEGAHAKVYEGKYRNQT-VAIKIIKRG
AT5G40540.1_Raf32          FY----SNE-VFELDPKWVVDPQHLFVGP--KIGEGAHAKIYEGKYKNKT-VAIKIVKRG
AT5G66710.1_Raf37          SN----DQF-AFTINTELLVDVKDISIGD--FIGEGSSSTVYRGLFRRVVPVSVKIFQPK
AT3G50720.1_Raf44          SD----NQF-DFNISRELLLNPKDIMRGE--MIGEGGNSIVYKGRLKNIVPVAVKIVQPG
AT3G50730.1_Raf45          DD----EPF-HFSISRELLLDRNDVVVGE--MIGEGAYSIVYKGLLRNQFPVAVKIMDPS
AT3G63260.1_Raf48/MRK1     AS----TSA-PAPAMQEWEIDLSKLDMKH--VLAHGTYGTVYRGVYAGQE-VAVKVLDWG
AT3G22750.1_Raf39          ----------HPKPKEEWEIELAKLEMRN--VIARGAYGIVYKGIYDGQD-VAVKVLDWG
AT4G14780.1_Raf26          ----------NPKAKEEWEIDLAKLETSN--VIARGTYGTVYKGIYDGQD-VAVKVLDWE
AT3G01490.1_Raf38          IR----STE-VEKSRREWEIDPSKLIIKS--VIARGTFGTVHRGIYDGQD-VAVKLLDWG
AT5G50000.1_Raf33          ----------KRKQRLEWEIDPSKLIIKT--VLARGTFGTVHRGIYDGQD-VAVKLLDWG
AT4G38470.1_Raf30          TH----VPI-PNDGTDVWEINLKHLKFGH--KIASGSYGDLYKGTYCSQE-VAIKVLKPE
AT2G17700.1_Raf21          AC----IEI-PTDGTDEWEIDVTQLKIEK--KVASGSYGDLHRGTYCSQE-VAIKFLKPD
AT4G35780.1_Raf29          AC----VEI-PTDGTDEWEIDMKQLKIEK--KVACGSYGELFRGTYCSQE-VAIKILKPE
AT2G24360.1_Raf22          -Y----PTE-GLTNYDEWTIDLRKLNMGP--AFAQGAFGKLYKGTYNGED-VAIKILERP
AT4G31170.1_Raf28          -Y----PTE-GLVNYEEWTIDLRKLHMGP--AFAQGAFGKLYRGTYNGED-VAIKLLERS
AT1G62400.1_Raf19/HT1      -T----WEA-SKGEREEWTADLSQLFIGN--KFASGAHSRIYRGIYKQRA-VAVKMVRIP
AT3G46930.1_Raf43          -S----KVS-SAGVLEECLIDVSKLSYGD--RFAHGKYSQIYHGEYEGKA-VALKIITAP
AT5G58950.1_Raf36          -R----RVS-AVEASEEFRVDMSKLFFGL--KFAHGLYSRLYHGKYEDKA-VAVKLITVP
                                                   :       .  *       .       *::*
```

```
AT1G04700.1_Raf16          CFSGG-SSEQARQTKDFWRE-----ARILANLHHPNVVAFYGVVPDG-------------
AT2G35050.1_Raf24          CFIGR-SSEQERLTSEFWHE-----AEILSKLHHPNVMAFYGVVKDG-------------
AT1G16270.1_Raf18          CFAGR-SSEQERLTGEFWGE-----AEILSKLHHPNVVAFYGVVKDG-------------
AT1G79570.1_Raf5           CFAGR-SSEQERLTGEFWGE-----AEILSKLHHPNVVAFYGVVKDG-------------
AT3G46920.1_Raf42          CFAGK-PSEQERMIDDFWNE-----AQNLAGLHHPNVVAFYGVVLDS-------------
AT5G57610.1_Raf35          CFAGK-PSERERLIEDFWKE-----ALLLSSLHHPNVVSFYGIVRDG-------------
AT1G08720.1_EDR1           DFSG-------AALAEFRSE-----VRIMRRLRHPNVVFFLGAVTRP-------------
AT5G11850.1_Raf3           DFSG-------DALTQFKSE-----IEIMLRLRHPNVVLFMGAVTRP-------------
AT1G18160.1_Raf4           DITG-------EALEEFRSE-----VRMMRRLRHPNIVLFMGAVTRP-------------
AT1G73660.1                DLTG-------EALEEFRSE-----VRIMKKLRHPNIVLFMGAVTRP-------------
AT4G24480.1_Raf6           DFHD-------DQFREFLREVCKQAVAIMKRVRHPNVVLFMGAVTER-------------
AT5G03730.2_Raf1/CTR1      DFHA-------ERVNEFLRE-----VAIMKRLRHPNIVLFMGAVTQP-------------
AT4G23050.2_Raf12          DYNA-------MTLTECKKE-----INIMKKLRHPNVLLFMGAVCTE-------------
AT5G49470.2_Raf10          EYSE-------EIITSFRQE-----VSLMKRLRHPNVLLFMGAVTSP-------------
AT1G67890.1_Raf11          EYSE-------EIITSFKQE-----VSLMKRLRHPNVLLFMGAVASP-------------
AT3G06620.1_Raf7           EYSA-------EVIESFKQE-----VLLMKRLRHPNVLLFMGAVTSP-------------
AT3G06630.1_Raf8           EYSE-------SVIKSFEKE-----VSLMKRLRHPNVLLFMGAVTSP-------------
AT3G06640.1_Raf9           EYSE-------EVIQSFRQE-----VSLMQRLRHPNVLLFMGAVTLP-------------
AT1G14000.1_VIK            LSDDR------LVIQDFRHE-----VDLLVKLRHPNIVQFLGAVTER-------------
AT2G31800.1_ILK3           LYKDS------DTINAFKHE-----LTLFEKVRHPNVVQFVGAVTQN-------------
AT2G43850.1_ILK1           SYSDP------ERINAFRHE-----LTLLEKVRHPNVIQFVGAVTQN-------------
AT3G59830.1_ILK2           SYSDP------ERVNAFTNE-----LTLLAKARHPNIVQFVGAVTQN-------------
AT3G58760.1_ILK4           MFTDE------DKVNAFRDE-----LALLQKIRHPNVVQFLGAVTQS-------------
AT4G18950.1_Raf27          VLSDD------DQVRKFHDE-----LALLQRLRHPNIVQFLGAVTQS-------------
AT2G31010.1_Raf13          DL-------TAENMEDFCNE-----ISILSRVRHPNVVLFLGACTKP-------------
AT3G58640.1_Raf15          DL-------TAENMEDFCNE-----ISILSRLRHPNVILFLGACTKP-------------
AT5G01850.1_Raf31          SKPDQ----QSSLESRFVRE-----VNMMSRVQHHNLVKFIGACKDP-------------
AT5G50180.1_Raf34          ETPEE----IAKRDSRFLRE-----VEMLSRVQHKNLVKFIGACKEP-------------
AT3G27560.1                ESPEE----IAKRDNRFARE-----IAMLSKVQHKNLVKFIGACKEP-------------
AT5G40540.1_Raf32          ESPEE----IAKRESRFARE-----VSMLSRVQHKNLVKFIGACKEP-------------
AT5G66710.1_Raf37          RTSAL----SIEQRKKFQRE-----VLLLSKFRHENIVRFIGACIEP-------------
AT3G50720.1_Raf44          KTSAV----SIQDKQQFQKE-----VLVLSSMKHENIVRFVGACIEP-------------
AT3G50730.1_Raf45          TTSAV----TKAHKKTFQKE-----VLLLSKMKHDNIVKFVGACIEP-------------
AT3G63260.1_Raf48/MRK1     EDGYATPAETTALRASFEQE-----VAVWQKLDHPNVTKFIGASMGTSDLRIPPAGDTGG
AT3G22750.1_Raf39          EDGYATTAETSALRASFRQE-----VAVWHKLDHPNVTRFVGASMGTANLKIPSSA----
AT4G14780.1_Raf26          DDGNETTAKTATNRALFRQE-----VTVWHKLNHPNVTKFVGASMGTTNLNIRS-A----
AT3G01490.1_Raf38          EEGHRSDAEIASLRAAFTQE-----VAVWHKLDHPNVTKFIGAAMGTSEMSIQTE-----
AT5G50000.1_Raf33          EEGHRSEAEIVSLRADFAQE-----VAVWHKLDHPNVTKFIGATMGASGLQLQTE-----
AT4G38470.1_Raf30          RL-------DSDLEKEFAQE-----VFIMRKVRHKNVVQFIGACTKP-------------
AT2G17700.1_Raf21          RV-------NNEMLREFSQE-----VFIMRKVRHKNVVQFLGACTRS-------------
AT4G35780.1_Raf29          RV-------NAEMLREFSQE-----VYIMRKVRHKNVVQFIGACTRS-------------
AT2G24360.1_Raf22          ENSPE---KAQFMEQQFQQE-----VSMLANLKHPNIVRFIGACRKP-------------
AT4G31170.1_Raf28          DSNPE---KAQALEQQFQQE-----VSMLAFLKHPNIVRFIGACIKP-------------
AT1G62400.1_Raf19/HT1      THKEE---TRAKLEQQFKSE-----VALLSRLFHPNIVQFIAACKKP-------------
AT3G46930.1_Raf43          EDSDD-IFLGARLEKEFIVE-----ATLLSRLSHPNVVKFVGVNT---------------
AT5G58950.1_Raf36          DDDDN-GCLGARLEKQFTKE-----VTLLSRLTHPNVIKFVGAYKDP-------------
                                               *             * *:  * .

AT1G04700.1_Raf16          ------PGGTMATVTEYMVNGSLRHVLQRKDRL-----------L---------------
AT2G35050.1_Raf24          ------PGGTLATVTEYMVNGSLRHVLLS-NRH-----------L---------------
AT1G16270.1_Raf18          ------PGATLATVTEYMVDGSLRHVLVRKDRH-----------L---------------
AT1G79570.1_Raf5           ------PGGTLATVTEYMVDGSLRHVLVRKDRH-----------L---------------
AT3G46920.1_Raf42          ------PGGSVATVTEYMVNGSLRNALQKNVRN-----------F---------------
AT5G57610.1_Raf35          ------PDGSLATVAEFMVNGSLKQFLQKKDRT-----------I---------------
AT1G08720.1_EDR1           --------PNLSIVTEFLPRGSLYRILHRPK--SH---------I---------------
AT5G11850.1_Raf3           --------PNFSILTEFLPRGSLYRLLHRPN--HQ---------L---------------
AT1G18160.1_Raf4           --------PNLSIVTEFLPRGSLYRLIHRPN--NQ---------L---------------
AT1G73660.1                --------PNLSIVTEFLPRGSLYRLIHRPN--NQ---------L---------------
AT4G24480.1_Raf6           --------PRLSIITEYLPRGSLFRLIHRPASGEL---------L---------------
AT5G03730.2_Raf1/CTR1      --------PNLSIVTEYLSRGSLYRLLHKSGAREQ---------L---------------
AT4G23050.2_Raf12          --------EKSAIIMEYMPRGSLFKILHNTNQP-----------L---------------
AT5G49470.2_Raf10          --------QRLCIVTEFLPRFGLITLANITLPFVLFELRGQNSDLPYQITFAVEVSSVCC
AT1G67890.1_Raf11          --------QRLCIVTEFLPRGSLFRLLQRNKSK-----------L---------------
AT3G06620.1_Raf7           --------QRLCIVSEFLPRGSLFRLLQKSTSK-----------L---------------
AT3G06630.1_Raf8           --------QRLCIVSEFVPRGSLFRLLQRSMSK-----------L---------------
AT3G06640.1_Raf9           --------QGLCIVSEFLPRGSLFRLLQRNMSK-----------L---------------
AT1G14000.1_VIK            --------KPLMLITEYLRGGDLHQYLKEK---GG---------L---------------
```

```
AT2G31800.1_ILK3          --------VPMMIVSEYHPKGDLGSYLQKK---GR---------L---------------
AT2G43850.1_ILK1          --------IPMMIVVEYNPKGDLSVYLQKK---GR---------L---------------
AT3G59830.1_ILK2          --------LPMMIVVECNPKGDLSVYLQKK---GR---------L---------------
AT3G58760.1_ILK4          --------TPMMIVTEYLPKGDLRQYLDRK---GP---------L---------------
AT4G18950.1_Raf27         --------NPMMIVTEYLPRGDLRELLKRK---GQ---------L---------------
AT2G31010.1_Raf13         --------PRLSMITEYMELGSLYYLIHMSGQKKK---------L---------------
AT3G58640.1_Raf15         --------PRLSLITEYMEMGSLYYLLHLSGQKKR---------L---------------
AT5G01850.1_Raf31         ---------LMVIVTELLPGMSLRKYLTSI-RPQL---------L---------------
AT5G50180.1_Raf34         ---------VMVIVTELLQGGTLRKYLLNL-RPAC---------L---------------
AT3G27560.1               ---------MMVIVTELLLGGTLRKYLVSL-RPKR---------L---------------
AT5G40540.1_Raf32         ---------IMVIVTELLLGGTLRKYLVSL-RPGS---------L---------------
AT5G66710.1_Raf37         ---------KLMIITELMEGNTLQKFMLSV-RPKP---------L---------------
AT3G50720.1_Raf44         ---------QLMIVTELVRGGTLQRFMLNS-RPSP---------L---------------
AT3G50730.1_Raf45         ---------QLIIVTELVEGGTLQRFM-HS-RPGP---------L---------------
AT3G63260.1_Raf48/MRK1    RGNGAHPARACCVVVEYVAGGTLKKFLIKK-YRAK---------L---------------
AT3G22750.1_Raf39         ETENSLPQRACCVVVEYIPGGTLKQYLFRN-RRKK---------L---------------
AT4G14780.1_Raf26         DSKGSLPQQACCVVVEYLPGGTLKQHLIRH-KSKK---------L---------------
AT3G01490.1_Raf38         NGQMGMPSNVCCVVVEYCPGGALKSFLIKT-RRRK---------L---------------
AT5G50000.1_Raf33         SGPLAMPNNICCVVVEYLPGGALKSYLIKN-RRRK---------L---------------
AT4G38470.1_Raf30         --------PHLCIVTEFMPGGSVYDYLHKQ-K-GV---------F---------------
AT2G17700.1_Raf21         --------PTLCIVTEFMARGSIYDFLHKQ-K-CA---------F---------------
AT4G35780.1_Raf29         --------PNLCIVTEFMTRGSIYDFLHKH-K-GV---------F---------------
AT2G24360.1_Raf22         --------MVWCIVTEYAKGGSVRQFLTRR-QNRA---------V---------------
AT4G31170.1_Raf28         --------MVWCIVTEYAKGGSVRQFLTKR-QNRA---------V---------------
AT1G62400.1_Raf19/HT1     --------PVYCIITEYMSQGNLRMYLNKK-EPYS---------L---------------
AT3G46930.1_Raf43         ---------GNCIITEYVPRGSLRSYLHKL-EQKS---------L---------------
AT5G58950.1_Raf36         --------PVYCVLTQYLPEGSLRSFLHKP-ENRS---------L---------------
                                       : :      :                     .

AT1G04700.1_Raf16         -----D--------R-------------------------RKKLMITLDSAFGMEYLHM-
AT2G35050.1_Raf24         -----D--------R-------------------------RKRLIIAMDAAFGMEYLHS-
AT1G16270.1_Raf18         -----D--------R-------------------------RKRLIIAMDAAFGMEYLHA-
AT1G79570.1_Raf5          -----D--------R-------------------------RKRLIIAMDAAFGMEYLHS-
AT3G46920.1_Raf42         -----D--------R-------------------------CKRQLIAMDIAFGMEYLHG-
AT5G57610.1_Raf35         -----D--------R-------------------------RKRLIIAMDTAFGMEYLHG-
AT1G08720.1_EDR1          -----D--------E-------------------------RRRIKMALDVAMGMNCLHTS
AT5G11850.1_Raf3          -----D--------E-------------------------KRRMRMALDVAKGMNYLHTS
AT1G18160.1_Raf4          -----D--------E-------------------------RKRLRMALDAARGMNYLHSC
AT1G73660.1               -----D--------E-------------------------RRRLRMALDAARGMNYLHSC
AT4G24480.1_Raf6          -----D--------Q-------------------------RRRLRMALDVAKGLNYLHCL
AT5G03730.2_Raf1/CTR1     -----D--------E-------------------------RRRLSMAYDVAKGMNYLHNR
AT4G23050.2_Raf12         -----D--------K-------------------------KRRLRMALDVARGMNYLHRR
AT5G49470.2_Raf10         RGTRQNWIGGDVSIWPRIFYLAYYSLDSLIQLLYLVYNMLHIFLTYFFAQARGMNYLHHC
AT1G67890.1_Raf11         -----D--------LRR-------------------------RIHMASDIARGMNYLHHC
AT3G06620.1_Raf7          -----D--------WRR-------------------------RIHMALDIARGMNYLHHC
AT3G06630.1_Raf8          -----D--------WRR-------------------------RINMALDIARGMNYLHCC
AT3G06640.1_Raf9          -----D--------WRR-------------------------RINMALDIARGMNYLHRC
AT1G14000.1_VIK           -----T--------P-------------------------TTAVNFALDIARGMTYLHN-
AT2G31800.1_ILK3          -----S--------P-------------------------AKVLRFALDIARGMNYLHEC
AT2G43850.1_ILK1          -----S--------P-------------------------SKALRFALDIARGMNYLHEC
AT3G59830.1_ILK2          -----S--------P-------------------------SKALRFALDIARGMNYLHEC
AT3G58760.1_ILK4          -----M--------P-------------------------AHAVKFALEIARGMNYLHEH
AT4G18950.1_Raf27         -----K--------P-------------------------ATAVRYALDIARGMSYLHEI
AT2G31010.1_Raf13         -----S--------W-------------------------HRRLRMLRDICRGLMCIHR-
AT3G58640.1_Raf15         -----S--------W-------------------------RRKLKMLRDICRGLMCIHR-
AT5G01850.1_Raf31         -----H--------L-------------------------PLALSFALDIARALHCLHA-
AT5G50180.1_Raf34         -----E--------T-------------------------RVAIGFALDIARGMECLHS-
AT3G27560.1               -----D--------I-------------------------RLAVGFALDIARAMECLHS-
AT5G40540.1_Raf32         -----D--------I-------------------------RVAVGYALDIARAMECLHS-
AT5G66710.1_Raf37         -----D--------L-------------------------KLSISFALDIARGMEFLNA-
AT3G50720.1_Raf44         -----D--------L-------------------------KVSLSFALDISRAMEYLHS-
AT3G50730.1_Raf45         -----D--------L-------------------------KMSLSFALDISRAMEFVHS-
AT3G63260.1_Raf48/MRK1    -----P--------I-------------------------KDVIQLALDLARGLSYLHS-
AT3G22750.1_Raf39         -----A--------F-------------------------KVVVQLALDLSRGLSYLHS-
AT4G14780.1_Raf26         -----A--------F-------------------------KAVIKLALDLARGLSYLHS-
AT3G01490.1_Raf38         -----A--------F-------------------------KVVIQLSLDLARGLSYLHS-
AT5G50000.1_Raf33         -----T--------F-------------------------KIVVQLALDLARGLSYLHS-
AT4G38470.1_Raf30         -----K--------L-------------------------PTLFKVAIDICKGMSYLHQ-
```

```
AT2G17700.1_Raf21        -----K--------L-------------------------QTLLKVALDVAKGMSYLHQ-
AT4G35780.1_Raf29        -----K--------I-------------------------QSLLKVALDVSKGMNYLHQ-
AT2G24360.1_Raf22        -----P--------L-------------------------KLAVKQALDVARGMAYVHG-
AT4G31170.1_Raf28        -----P--------L-------------------------KLAVMQALDVARGMAYVHE-
AT1G62400.1_Raf19/HT1    -----S--------I-------------------------ETVLRLALDISRGMEYLHS-
AT3G46930.1_Raf43        -----P--------L-------------------------EQLIDFGLDIAKGMEYIHS-
AT5G58950.1_Raf36        -----P--------L-------------------------KKLIEFAIDIARGMEYIHS-
                                                                           . .:  :.

                               HRDLxxxN                DFG
AT1G04700.1_Raf16        --KNIVHFDLKCDNLLVNLRDPQRPICKVGDFGLSRIKRNT-------LVSGGVRGTLPW
AT2G35050.1_Raf24        --KSIVHFDLKCDNLLVNLKDPARPICKVGDFGLSKIKRNT-------LVTGGVRGTLPW
AT1G16270.1_Raf18        --KNIVHFDLKCDNLLVNLKDPSRPICKVGDFGLSKIKRNT-------LVSGGVRGTLPW
AT1G79570.1_Raf5         --KNTVHFDLKCDNLLVNLKDPSRPICKVGDFGLSKIKRNT-------LVSGGVRGTLPW
AT3G46920.1_Raf42        --KKIVHFDLKSDNLLVNLRDPHRPICKVGDLGLSKVKCQT-------LISGGVRGTLPW
AT5G57610.1_Raf35        --KNIVHFDLKCENLLVNMRDPQRPICKIGDLGLSKVKQKT-------LVSGGVRGTLPW
AT1G08720.1_EDR1         TP-TIVHRDLKTPNLLVDN----NWNVKVGDFGLSRLKHNT------FLSSKSTAGTPEW
AT5G11850.1_Raf3         HP-TVVHRDLKSPNLLVDK----NWVVKVCDFGLSRMKHHT------YLSSKSTAGTPEW
AT1G18160.1_Raf4         NP-VIVHRDLKSPNLLVDK----NWVVKVCDFGLSRMKVST------YLSSKSTAGTAEW
AT1G73660.1              NP-MIVHRDLKSPNLLVDK----NWVVKVCDFGLSRMKHST------YLSSKSTAGTAEW
AT4G24480.1_Raf6         NP-PVVHWDLKSPNLLVDK----NWTVKVCDFGLSRFKANT------FIPSKSVAGTPEW
AT5G03730.2_Raf1/CTR1    NP-PIVHRDLKSPNLLVDK----KYTVKVCDFGLSRLKAST------FLSSKSAAGTPEW
AT4G23050.2_Raf12        NP-PIVHRDLKSSNLLVDK----NWNVKVGDFGLSKWKNAT------FLSTKSGKGTPQW
AT5G49470.2_Raf10        TP-PIIHRDLKSSNLLVDK----NWTVKVADFGLSRIKHET------YLTTKTGRGTPQW
AT1G67890.1_Raf11        SP-PIIHRDLKSSNLLVDR----NWTVKVADFGLSRIKHET------YLTT-NGRGTPQW
AT3G06620.1_Raf7         SP-PIIHRDLKSSNLLVDK----NWTVKVADFGLSRIKHET------YLTSKSGKGTPQW
AT3G06630.1_Raf8         SP-PIIHRDLKSSNLLVDR----NWTVKVADFGLSRIKHQT------YLTSKSGKGTPQW
AT3G06640.1_Raf9         SP-PIIHRDLKSSNLLVDK----NLTVKVADFGLSRIKHHT------YLTSKSGKGMPQW
AT1G14000.1_VIK          EPNVIIHRDLKPRNVLLVNSS--ADHLKVGDFGLSKLIKVQNSH--DVYKMTGETGSYRY
AT2G31800.1_ILK3         KPEPVIHCDLKPKNIMLD--S--GGHLKVAGFGLISFAKLSSDKSKILNHGAHIDPSNYC
AT2G43850.1_ILK1         KPDPIIHCDLKPKNILLD--R--GGQLKISGFGMIRLSKISQDKAKVANHKAHIDLSNYY
AT3G59830.1_ILK2         KPDPIIHCELMPKNILLD--R--GGQLKISGFGLIKLSKIGEDSAKVVNHEAQIDKSNYY
AT3G58760.1_ILK4         KPEAIIHCDLEPPNILRD--D--SGHLKVADFGVSKLLVVKKTVKKDRPVVTCLDSSWRY
AT4G18950.1_Raf27        KGDPIIHRDLEPSNILRD--D--SGHLKVADFGVSKLVTVKE-----DKPFTCQDISCRY
AT2G31010.1_Raf13        --MKIVHRDLKSANCLVDK----HWTVKICDFGLSRIMTDE------NMKDTSSAGTPEW
AT3G58640.1_Raf15        --MGIVHRDIKSANCLLSN----KWTVKICDFGLSRIMTGT------TMRDTVSAGTPEW
AT5G01850.1_Raf31        --NGIIHRDLKPDNLLLTE-N--HKSVKLADFGLAREESVT-------EMMTAETGTYRW
AT5G50180.1_Raf34        --HGIIHRDLKPENLLLTA-D--HKTVKLADFGLAREESLT-------EMMTAETGTYRW
AT3G27560.1              --HGIIHRDLKPENLILSA-D--HKTVKLADFGLAREESLT-------EMMTAETGTYRW
AT5G40540.1_Raf32        --HGVIHRDLKPESLILTA-D--YKTVKLADFGLAREESLT-------EMMTAETGTYRW
AT5G66710.1_Raf37        --NGIIHRDLKPSNMLLTG-D--QKHVKLADFGLAREETKG--------FMTFEAGTYRW
AT3G50720.1_Raf44        --KGIIHRDLNPRNVLVTG-D--MKHVKLADFGLAREKTLG--------GMTCEAGTYRW
AT3G50730.1_Raf45        --NGIIHRDLNPRNLLVTG-D--LKHVKLADFGIAREETRG--------GMTCEAGTSKW
AT3G63260.1_Raf48/MRK1   --KAIVHRDVKSENMLLQP----NKTLKIADFGVARVEAQN------PQDMTGETGTLGY
AT3G22750.1_Raf39        --ERIVHRDVKTENMLLDY----QRNLKIADFGVARVEAQN------PKDMTGETGTLGY
AT4G14780.1_Raf26        --EKIVHRDVKTENMLLDA----QKNLKIADFGVARVEALN------PKDMTGETGTLGY
AT3G01490.1_Raf38        --QKIVHRDVKTENMLLDK----SRTLKIADFGVARLEASN------PNDMTGETGTLGY
AT5G50000.1_Raf33        --QKIVHRDVKTENMLLDK----TRTVKIADFGVARVEASN------PNDMTGETGTLGY
AT4G38470.1_Raf30        --NNIIHRDLKAANLLMDE----NEVVKVADFGVARVKAQT-------GVMTAETGTYRW
AT2G17700.1_Raf21        --NNIIHRDLKTANLLMDE----HGLVKVADFGVARVQIES-------GVMTAETGTYRW
AT4G35780.1_Raf29        --NNIIHRDLKTANLLMDE----HEVVKVADFGVARVQTES-------GVMTAETGTYRW
AT2G24360.1_Raf22        --RNFIHRDLKSDNLLISA----DKSIKIADFGVARIEVQT-------EGMTPETGTYRW
AT4G31170.1_Raf28        --RNFIHRDLKSDNLLISA----DRSIKIADFGVARIEVQT-------EGMTPETGTYRW
AT1G62400.1_Raf19/HT1    --QGVIHRDLKSNNLLLND----EMRVKVADFGTSCLETQC-------REAKGNMGTYRW
AT3G46930.1_Raf43        --REIVHQDLKPENVLIDN----DFHLKIADFGIACEEEYC-------DVLGDNIGTYRW
AT5G58950.1_Raf36        --RRIIHRDLKPENVLIDE----EFHLKIADFGIACEEEYC-------DMLADDPGTYRW
                              :* ::   . :           *:  :*

AT1G04700.1_Raf16        MAPEL---------LNGSSNRVSEKVDVFSFGIVMWEILTGEEPYANLHC-GAIIGGIVN
AT2G35050.1_Raf24        MAPEL---------LSGSSSKVSEKVDVFSFGIVLWEILTGEEPYANMHY-GAIIGGIVN
AT1G16270.1_Raf18        MAPEL---------LNGSSSKVSEKVDVFSFGIVLWEILTGEEPYANMHY-GAIIGGIVN
AT1G79570.1_Raf5         MAPEL---------LNGSSSKVSEKVDVFSFGIVLWEILTGEEPYANMHY-GAIIGGIVN
AT3G46920.1_Raf42        MAPEL---------LNGTSSLVSEKVDVFSFGIVLWELFTGEEPYADLHY-GAIIGGIVS
AT5G57610.1_Raf35        MAPEL---------LSGKSNMVSEKIDVYSFGIVMWELLTGEEPYADMHC-ASIIGGIVN
AT1G08720.1_EDR1         MAPEV---------LRNE--PSNEKCDVYSFGVILWELATLRLPWRGMNP-MQVVGAVGF
AT5G11850.1_Raf3         MAPEV---------LRNE--PANEKCDVYSFGVILWELATSRVPWKGLNP-MQVVGAVGF
AT1G18160.1_Raf4         MAPEV---------LRNE--PADEKCDVYSYGVILWELFTLQQPWGKMNP-MQVVGAVGF
AT1G73660.1              MAPEV---------LRNE--PADEKCDVYSYGVILWELFTLQQPWGKMNP-MQVVGAVGF
AT4G24480.1_Raf6         MAPEF---------LRGE--PTNEKSDVYSFGVVLWELITLQQPWNGLSP-AQVVGAVAF
```

```
AT5G03730.2_Raf1/CTR1        MAPEV---------LRDE--PSNEKSDVYSFGVILWELATLQQPWGNLNP-AQVVAAVGF
AT4G23050.2_Raf12            MAPEV---------LRSE--PSNEKCDVFSFGVILWELMTTLVPWDRLNS-IQVVGVVGF
AT5G49470.2_Raf10            MAPEV---------LRNE--AADEKSDVYSFGVILWELVTEKIPWESLNA-MQVIGAVGF
AT1G67890.1_Raf11            MAPEV---------LRNE--AADEKSDVYSFGVVLWELVTEKIPWENLNA-MQVIGAVGF
AT3G06620.1_Raf7             MAPEV---------LRNE--SADEKSDIYSFGVVLWELATEKIPWETLNS-MQVIGAVGF
AT3G06630.1_Raf8             MAPEV---------LRNE--SADEKSDIYSFGVVLWELATEKIPWENLNS-MQVIGAVGF
AT3G06640.1_Raf9             MAPEV---------LRNE--SADEKSDIYSFGVVLWELATEKIPWENLNS-MQVIGAVGF
AT1G14000.1_VIK              MAPEV---------FKHR--RYDKKVDVFSFAMILYEMLEGEPPFANHEP-YEAAK-HVS
AT2G31800.1_ILK3             MAPEV---------YKDE--IFDRSVDSYSFGVVLYEMIEGVQPFHPKPP-EEAVKLMCL
AT2G43850.1_ILK1             IAPEV---------YKDE--IFDLRVDAHSFGVILYEITEGVPVFHPRPP-EEVARMMCL
AT3G59830.1_ILK2             IAPEI---------YKDE--VFDKRADVHSFGVILYELTEGVSLFHPKPP-EEVAESICI
AT3G58760.1_ILK4             MAPEV---------YRNE--EYDTKVDVFSFALILQEMIEGCEPFHEIED-REVPK-AYI
AT4G18950.1_Raf27            IAPEV---------FTSE--EYDTKADVFSFALIVQEMIEGRMPFAEKED-SEASE-AYA
AT2G31010.1_Raf13            MAPEL---------IRNR--PFTEKCDIFSLGVIMWELSTLRKPWEGVPP-EKVVFAVAH
AT3G58640.1_Raf15            MAPEL---------IRNE--PFSEKCDIFSLGVIMWELCTLTRPWEGVPP-ERVVYAIAY
AT5G01850.1_Raf31            MAPEL-YSTVTLRQGEKK--HYNNKVDVYSFGIVLWELLTNRMPFEGMSN-LQAAYAAAF
AT5G50180.1_Raf34            MAPEL-YSTVTLRLGEKK--HYNHKVDAYSFAIVLWELLHNKLPFEGMSN-LQAAYAAAF
AT3G27560.1                  MAPEL-YSTVTLRQGEKK--HYNHKVDAYSFAIVLWELILNKLPFEGMSN-LQAAYAAAF
AT5G40540.1_Raf32            MAPEL-YSTVTLRHGEKK--HYNHKVDAYSFAIVLWELIHNKLPFEGMSN-LQAAYAAAF
AT5G66710.1_Raf37            MAPEL-FSYDTLEIGEKK--HYDHKVDVYSFAIVFWELLTNKTPFKGKNN--IFVAYAAS
AT3G50720.1_Raf44            MAPEV-CSREPLRIGEKK--HYDQKIDVYSFALIFWSLLTNKTPFSEIPS--ISIPYFVN
AT3G50730.1_Raf45            MAPEVVYSPEPLRVGEKK--EYDHKADIYSFAIVLWQLVTNEEPFPDVPN-SLFVPYLVS
AT3G63260.1_Raf48/MRK1       MAPEV---------LEGK--PYNRKCDVYSFGVCLWEIYCCDMPYADCSF-AEISHAVVH
AT3G22750.1_Raf39            MAPEV---------LDGK--PYNRRCDVYSFGICLWEIYCCDMPYPDLSF-ADVSSAVVR
AT4G14780.1_Raf26            MAPEV---------IDGK--PYNRRCDVYSFGICLWEIYCCDMPYPDLSF-VDVSSAVVL
AT3G01490.1_Raf38            MAPEV---------LNGS--PYNRKCDVYSFGICLWEIYCCDMPYPDLSF-SEVTSAVVR
AT5G50000.1_Raf33            MAPEV---------LNGN--PYNRKCDVYSFGICLWEIYCCDMPYPDLTF-SEVTSAVVR
AT4G38470.1_Raf30            MAPEV---------IEHK--PYDHKADVFSYGIVLWELLTGKLPYEYMTP-LQAAVGVVQ
AT2G17700.1_Raf21            MAPEV---------IEHK--PYNHKADVFSYAIVLWELLTGDIPYAFLTP-LQAAVGVVQ
AT4G35780.1_Raf29            MAPEV---------IEHK--PYDHRADVFSYAIVLWELLTGELPYSYLTP-LQAAVGVVQ
AT2G24360.1_Raf22            MAPEM---------IQHR--AYNQKVDVYSFGIVLWELITGLLPFQNMTA-VQAAFAVVN
AT4G31170.1_Raf28            MAPEM---------IQHR--PYTQKVDVYSFGIVLWELITGLLPFQNMTA-VQAAFAVVN
AT1G62400.1_Raf19/HT1        MAPEM---------IKEK--PYTRKVDVYSFGIVLWELTTALLPFQGMTP-VQAAFAVAE
AT3G46930.1_Raf43            MAPEV---------LKRI--PHGRKCDVYSFGLLLWEMVAGALPYEEMKFAEQIAYAVIY
AT5G58950.1_Raf36            MAPEM---------IKRK--PHGRKADVYSFGLVLWEMVAGAIPYEDMNP-IQAAFAVVH
                             :***.                     * .* .: . .:      :

AT1G04700.1_Raf16            NTLRPPVP---ERCEAEWRKLMEQCWSFDPGVRPSFTEIVERLRSMTVALQPKRRT----
AT2G35050.1_Raf24            NTLRPTVP---NYCDPEWRMLMEQCWAPDPFVRPAFPEIARRLRTMSSSAVHTKPHAVNH
AT1G16270.1_Raf18            NTLRPTIP---SYCDSDWRILMEECWAPNPTARPSFTEIAGRLRVMSTAATSNQSKPPAH
AT1G79570.1_Raf5             NTLRPTIP---GFCDDEWRTLMEECWAPNPMARPSFTEIAGRLRVMSSAATSTQSKPSAH
AT3G46920.1_Raf42            NTLRPQIP---DFCDMDWKLLMERCWSAEPSERPSFTEIVNELRTMATKLPSKEQGSTQG
AT5G57610.1_Raf35            NALRPKIP---QWCDPEWKGLMESCWTSEPTERPSFTEISQKLRTMAAAMNLK-------
AT1G08720.1_EDR1             QNRRLEIP---KELDPVVGRIILECWQTDPNLRPSFAQLTEVLKPLNRLVLPTPQ-----
AT5G11850.1_Raf3             QNRRLEIP---DDIDLTVAQIIRECWQTEPHLRPSFTQLMQSLKRLQGLNISNR------
AT1G18160.1_Raf4             QHRRLDIP---EFVDPGIADIIRKCWQTDPRLRPSFGEIMDSLKQLQKPIQRAAVPSS--
AT1G73660.1                  QHRRLDIP---DFVDPAIADLISKCWQTDSKLRPSFAEIMASLKRLQKPVTGSNIPRP--
AT4G24480.1_Raf6             QNRRLIIP---PNTSPVLVSLMEACWADEPSQRPAFGSIVDTLKKLLKSPVQLIQMGGDK
AT5G03730.2_Raf1/CTR1        KCKRLEIP---RNLNPQVAAIIEGCWTNEPWKRPSFATIMDLLRPLIKSAVPPPNRSDL-
AT4G23050.2_Raf12            MDRRLDLP---EGLNPRIASIIQDCWQTDPAKRPSFEELISQMMSLFRKPGSGAQEEDD-
AT5G49470.2_Raf10            MNQRLEVP---KNVDPQWISLMESCWHSEPQDRPSFQEIMEKLRELQRKYTIQFQAARAA
AT1G67890.1_Raf11            MNQRLEVP---KDVDPQWIALMESCWHSEPQCRPSFQELMDKLRELQRKYTIQFQAARAA
AT3G06620.1_Raf7             MDQRLEIP---KDIDPRWISLMESCWHSDTKLRPTFQELMDKLRDLQRKYMIQFQATRAA
AT3G06630.1_Raf8             MNQRLEIP---KDTDPDWISLIESCWHR--------------------------------
AT3G06640.1_Raf9             MNQRLEIP---KDIDPDWISLIESCWHRDAKLRPTFQELMERLRDLQRKYTIQFQATRWL
AT1G14000.1_VIK              DGHRPTFR--SKGCTPDLRELIVKCWDADMNQRPSFLDILKRLEKIKETLPSDHHWGLFT
AT2G31800.1_ILK3             EGRRPSFKAKSKSCPQEMRELIEECWDTETFVRPTFSEIIVRLDKIFVHCSKQGWWKDTF
AT2G43850.1_ILK1             EGKRPVFKTKSRSYPPDIKELIEKCWHPEAGIRPTFSEIIIRLDKIVANCSKQGWWKDTF
AT3G59830.1_ILK2             EGKRPTIRTKSKSYPPELKELIEECWHPEISVRPIFSEIIIRLDKIVTNCSKQGWWKDTF
AT3G58760.1_ILK4             EDERPPFNAPTKSYPFGLQELIQDCWDKEASKRPTFRVIISTLELISDRIARKRSWKVML
AT4G18950.1_Raf27            GKHRPLFKAPSKNYPHGLKTLIEECWHEKPAKRPTFREIIKRLESILHHMGHKRQWRMRP
AT2G31010.1_Raf13            EGSRLEIP---DG---PLSKLIADCWA-EPEERPNCEEILRGLLDCEYTLC---------
AT3G58640.1_Raf15            EGARLEIP---EG---PLGKLIADCWT-EPEQRPSCNEILSRLLDCEYSLC---------
AT5G01850.1_Raf31            KQERPVMP---EGISPSLAFIVQSCWVEDPNMRPSFSQIIRLLNEFLLTLTPPPPQPLPE
AT5G50180.1_Raf34            KNVRPSA----ESLPEELGDIVTSCWNEDPNARPNFTHIIELLLNYLSKVGSPISA--IP
AT3G27560.1                  KNLRPSA----EDLPGDLEMIVTSCWKEDPNERPNFTEIIQMLLRYLTTVSAPQIIPPPN
AT5G40540.1_Raf32            KNVRPSA----DDLPKDLAMIVTSCWKEDPNDRPNFTEIIQMLLRCLSTISSTELVPPAI
AT5G66710.1_Raf37            KNQRPSV----ENLPEGVVSILQSCWAENPDARPEFKEITYSLTNLLRSLSSDTDATSSN
```

```
AT3G50720.1_Raf44          QGKRPSL----SNIPDEVVPILECCWAADSKTRLEFKDITISLESLLKRFCSERSNNEIT
AT3G50730.1_Raf45          QGRRPIL----TKTPDVFVPIVESCWAQDPDARPEFKEISVMLTNLLRRMSSDSS-IGTT
AT3G63260.1_Raf48/MRK1     RNLRPEIP---KCCPHAVANIMKRCWDPNPDRRPEMEEVVKLLEAIDTSKGGGMIAPDQ-
AT3G22750.1_Raf39          QNLRPDIP---RCCPTALATIMKRCWEANPEKRPEMEEVVSLLEAVDTTKGGGMIPEDQ-
AT4G14780.1_Raf26          HNLRPEIP---RCCPTALAGIMKTCWDGNPQKRPEMKEVVKMLEGVDTSKGGGMIPEDQ-
AT3G01490.1_Raf38          QNLRPEIP---RCCPSSLANVMKRCWDANPEKRPEMEEVVAMLEAIDTSKGGGMIPPDQ-
AT5G50000.1_Raf33          QNLRPDIP---RCCPSALAAVMKRCWDANPDKRPEMDEVVPMLESIDTTKGGGMIPNDQ-
AT4G38470.1_Raf30          KGLRPTIP---KNTHPKLAELLERLWEHDSTQRPDFSEIIEQLQEIAKEVGEEGEEKKKS
AT2G17700.1_Raf21          KGLRPKIP---KKTHPKVKGLLERCWHQDPEQRPLFEEIIEMLQQIMKEVNVVV------
AT4G35780.1_Raf29          KGLRPKIP---KETHPKLTELLEKCWQQDPALRPNFAEIIEMLNQLIREVGDDERHKDK-
AT2G24360.1_Raf22          RGVRPTVP---NDCLPVLSDIMTRCWDANPEVRPCFVEVVKLLEAAETEIMTTARKA---
AT4G31170.1_Raf28          RGVRPTVP---ADCLPVLGEIMTRCWDADPEVRPCFAEIVNLLEAAETEIMTNVRKA---
AT1G62400.1_Raf19/HT1      KNERPPLP---ASCQPALAHLIKRCWSENPSKRPDFSNIVAVLEKYDECVKEGLPLTSHA
AT3G46930.1_Raf43          KKIRPVIP---TDCPAAMKELIERCWSSQTDKRPEFWQIVKVLEHFKKSLTSEGKLN---
AT5G58950.1_Raf36          KNIRPAIP---GDCPVAMKALIEQCWSVAPDKRPEFWQIVKVLEQFAISLEREGNLN---
                              *                ::   *

AT1G04700.1_Raf16          ------------------------------------------------------------
AT2G35050.1_Raf24          QIHK--------------------------------------------------------
AT1G16270.1_Raf18          KASK--------------------------------------------------------
AT1G79570.1_Raf5           RASK--------------------------------------------------------
AT3G46920.1_Raf42          PQS---------------------------------------------------------
AT5G57610.1_Raf35          ------------------------------------------------------------
AT1G08720.1_EDR1           ------------------------------------------------------------
AT5G11850.1_Raf3           --ANTSESLM--------------------------------------------------
AT1G18160.1_Raf4           ------SALTTDE--------------QEQ------------------------------
AT1G73660.1                --VPSSSSLPTEH--------------EQKD-----------------------------
AT4G24480.1_Raf6           GVIPTKSAPIL-------------------------------------------------
AT5G03730.2_Raf1/CTR1      ------------------------------------------------------------
AT4G23050.2_Raf12          ------------------------------------------------------------
AT5G49470.2_Raf10          SIENSALKEK--------------------------------------------------
AT1G67890.1_Raf11          SIDNSSLK----E--------------K--------------------------------
AT3G06620.1_Raf7           LSD----NSLLKD--------------N--------------------------------
AT3G06630.1_Raf8           ------------------------------------------------------------
AT3G06640.1_Raf9           TMVRTESSQVLKQ--------------NH-------------------------------
AT1G14000.1_VIK            S-----------------------------------------------------------
AT2G31800.1_ILK3           K-------FPWK------------------------------------------------
AT2G43850.1_ILK1           K-------FPWK------------------------------------------------
AT3G59830.1_ILK2           K-------FPWK------------------------------------------------
AT3G58760.1_ILK4           G-------RCLPR--F-----RLFTKRDYV-NPGGSNR---SSG---SFNR---------
AT4G18950.1_Raf27          L-------TCFQN--F-----EHKKKHNWD-LSSHDGS---SSG---SHL----------
AT2G31010.1_Raf13          ------------------------------------------------------------
AT3G58640.1_Raf15          ------------------------------------------------------------
AT5G01850.1_Raf31          TATNRTNGRAIT---------------------------------EFSIRPKGKFAFI--
AT5G50180.1_Raf34          QRILASKNTLLPP--DSPGTSSLMAKLDECGETPKA---------KSEDKRKGLF--F--
AT3G27560.1                RRVFSSENIVLSP--ESPGTCSLMSVRDGDVSRQTV-----NTADSSEKQTKGSF-FS--
AT5G40540.1_Raf32          KRVFSSENTVLPP--ESPGTCSLMTVRDKDQIPTDA-----N---SAQNEVRGSFFFF--
AT5G66710.1_Raf37          ----SKANIATED-----STSSLVQERVVCDCPGLKMSKTKKLKKKTNKLMNMIVPFLKI
AT3G50720.1_Raf44          ----ITEDEAYDDEIEELETTWLLPKRY-I-----KLKK-PKKIK--QNVMKKILPFFKK
AT3G50730.1_Raf45          ----LPDGEAYEGEMEESENSPLLQEHF-C-----KVKK-PKEKKKKKKLVKMRFPFFKK
AT3G63260.1_Raf48/MRK1     ----FQGCLCFFK--PR-GP----------------------------------------
AT3G22750.1_Raf39          ----RPGCFCFVS--GR-GP----------------------------------------
AT4G14780.1_Raf26          ----SRGCFCFAP--AR-GP----------------------------------------
AT3G01490.1_Raf38          ----QQGCFCFRR--HR-GP----------------------------------------
AT5G50000.1_Raf33          ----QQGCLCFRR--KR-GP----------------------------------------
AT4G38470.1_Raf30          STGL-GGGIFAAL--RR-STTHH-------------------------------------
AT2G17700.1_Raf21          ------------------------------------------------------------
AT4G35780.1_Raf29          -----HGGYFSGL--KK-GHR---------------------------------------
AT2G24360.1_Raf22          -----------RF--RC-CLSQPMTID---------------------------------
AT4G31170.1_Raf28          -----------RF--RC-CMTQPMTVD---------------------------------
AT1G62400.1_Raf19/HT1      SLTKTKKAILDHL--KG-CVTSI------------------SSPFSSSSVPVNA------
AT3G46930.1_Raf43          ---LLPSQICPEL--KK-CPKFWIHIFGSFHHHSSGGG--SSS--NNSALPKPKFA----
AT5G58950.1_Raf36          ---LSSSKICKDP--RK-GLKHWIQKLGPVHAGGGGGS--SSSGLGGSALPKPKFA----


AT1G04700.1_Raf16          ----------------
AT2G35050.1_Raf24          ----------------
AT1G16270.1_Raf18          ----------------
```

```
AT1G79570.1_Raf5            ----------------
AT3G46920.1_Raf42           ----------------
AT5G57610.1_Raf35           ----------------
AT1G08720.1_EDR1            ----------------
AT5G11850.1_Raf3            ----------------
AT1G18160.1_Raf4            ----------------
AT1G73660.1                 ----------------
AT4G24480.1_Raf6            ----------------
AT5G03730.2_Raf1/CTR1       ----------------
AT4G23050.2_Raf12           ----------------
AT5G49470.2_Raf10           ----------------
AT1G67890.1_Raf11           ----------------
AT3G06620.1_Raf7            ----------------
AT3G06630.1_Raf8            ----------------
AT3G06640.1_Raf9            ----------------
AT1G14000.1_VIK             ----------------
AT2G31800.1_ILK3            ----------------
AT2G43850.1_ILK1            ----------------
AT3G59830.1_ILK2            ----------------
AT3G58760.1_ILK4            ----------------
AT4G18950.1_Raf27           ----------------
AT2G31010.1_Raf13           ----------------
AT3G58640.1_Raf15           ----------------
AT5G01850.1_Raf31           ----RQLFAAKRNINS
AT5G50180.1_Raf34           --CFNQCY--------
AT3G27560.1                 --CCS-----------
AT5G40540.1_Raf32           --CC------------
AT5G66710.1_Raf37           FKSCMSK---------
AT3G50720.1_Raf44           FISSKW----------
AT3G50730.1_Raf45           FKVWLYNYKP------
AT3G63260.1_Raf48/MRK1      ----------------
AT3G22750.1_Raf39           ----------------
AT4G14780.1_Raf26           ----------------
AT3G01490.1_Raf38           ----------------
AT5G50000.1_Raf33           ----------------
AT4G38470.1_Raf30           ----------------
AT2G17700.1_Raf21           ----------------
AT4G35780.1_Raf29           ----------------
AT2G24360.1_Raf22           ----------------
AT4G31170.1_Raf28           ----------------
AT1G62400.1_Raf19/HT1       ----------------
AT3G46930.1_Raf43           ----------------
AT5G58950.1_Raf36           ----------------
```

PLEASE NOTE: Showing colors on large alignments is slow.
