# Supplementary material for: Insights into the Structure, Function, and Ion-Mediated Signaling Pathways Transduced by Plant Integrin-Linked Kinases
Source: Front Plant Sci. 2017 Apr 3;8:376. doi: 10.3389/fpls.2017.00376 (PMC5376563; doi:10.3389/fpls.2017.00376)
Supplement: TABLE S1 — Interaction partners of the Arabidopsis ILKs. [file Table_1.docx]

**Supplementary Table 1** lists the interaction partners of the Arabidopsis ILKs, their IDs, symbols (if known) and function.

| **ILK protein** | **ILK ID** | **Interactor** | **Interactor ID** | **Interactor function** |
| --- | --- | --- | --- | --- |
| ILK1 | AT2G43850 | CAM9 | AT3G51920 | Ca2+-binding, signaling |
| ILK1 | AT2G43850 | CPK15 | AT4G21940 | Ca2+-binding, signaling |
| ILK1 | AT2G43850 | CAM1 | AT5G37780 | Ca2+-binding, signaling |
| ILK1 | AT2G43850 | ANK1 | AT5G02620 | Protein-binding |
| ILK1 | AT2G43850 | CERK1 | AT3G21630 | Receptor/Signal transduction |
| ILK1 | AT2G43850 | CRK40 | AT4G04570 | Receptor/Signal transduction |
| ILK1 | AT2G43850 | RGS1 | AT3G26090 | Signal transduction |
| ILK1 | AT2G43850 | MLO4 | AT1G11000 | Trafficking/exocytosis |
| ILK1 | AT2G43850 | NRT1.1 | AT1G12110 | Transporter (nitrate transporter) |
| ILK1 | AT2G43850 | NRT1.7 | AT1G69870 | Transporter (nitrate transporter) |
| ILK1 | AT2G43850 | PTR4 | AT2G02020 | Transporter (peptide transporter) |
| ILK1 | AT2G43850 | PTR2 | AT2G02040 | Transporter (peptide transporter) |
| ILK1 | AT2G43850 | CHX19 | AT3G17630 | Transporter (cation/H+ exchanger) |
| ILK1 | AT2G43850 | CHX10 | AT3G44930 | Transporter (cation/H+ exchanger) |
| ILK1 | AT2G43850 | PTR1 | AT3G54140 | Transporter (peptide transporter) |
| ILK1 | AT2G43850 | MscS | AT4G00290 | Transporter (Mechanosensitive ion channel) |
| ILK1 | AT2G43850 | CNGC17 | AT4G30360 | Transporter (cyclic nucleotide-gated channel ) |
| ILK1 | AT2G43850 | PTR5 | AT5G01180 | Transporter (peptide transporter) |
| ILK1 | AT2G43850 |  | AT5G41800 | Transporter (amino acid transporter) |
| ILK1 | AT2G43850 | HAK5 | AT4G13420 | Transporter (potassium transporter) |
| ILK1 | AT2G43850 | GRF4 | AT1G35160 | Signal transduction |
| ILK3 | AT2G31800 | SYP43 | AT3G05710 | Trafficking/exocytosis |
| ILK3 | AT2G31800 | PEN1_SYP121 | AT3G11820 | Trafficking/exocytosis |
| ILK3 | AT2G31800 | SYP123 | AT4G03330 | Trafficking/exocytosis |
| ILK3 | AT2G31800 | SYP132 | AT5G08080 | Trafficking/exocytosis |
| ILK4 | AT3G58760 | CSLC4 | AT3G28180 | Cell wall |
| ILK4 | AT3G58760 | TBL36 | AT3G54260 | Cell wall |
| ILK4 | AT3G58760 | PK | AT1G11050 | Signal transduction |
| ILK4 | AT3G58760 | SERK4 | AT2G13790 | Signal transduction |
| ILK4 | AT3G58760 | PK | AT4G02010 | Signal transduction |
| ILK4 | AT3G58760 | GLR2.9 | AT2G29100 | Transporter (glutamate receptor) |
| ILK5 | AT4G18950 | Ca-binding EF-hand | AT2G41090 | Ca2+-binding, signaling |
| ILK5 | AT4G18950 | CAM4_TCH3 | AT2G41100 | Ca2+-binding, signaling |
| ILK5 | AT4G18950 | CAM7 | AT3G43810 | Ca2+-binding, signaling |
| ILK5 | AT4G18950 | CAM9 | AT3G51920 | Ca2+-binding, signaling |
| ILK5 | AT4G18950 | CML8 | AT4G14640 | Ca2+-binding, signaling |
| ILK5 | AT4G18950 | CAM6 | AT5G21274 | Ca2+-binding, signaling |
| ILK5 | AT4G18950 | CAM1 | AT5G37780 | Ca2+-binding, signaling |
| ILK5 | AT4G18950 | IRX15 | AT3G50220 | Cell wall |
| ILK5 | AT4G18950 | RING/U-box | AT1G24440 | Protein degradation |
| ILK5 | AT4G18950 | LRR-RLK | AT3G03770 | Receptor/Signal transduction |
| ILK5 | AT4G18950 | MPK7 | AT2G18170 | Signal transduction |
| ILK5 | AT4G18950 | ATPI4K | AT2G46500 | Signal transduction |
| ILK5 | AT4G18950 | AMT1;4 | AT4G28700 | Transporter (ammonium transporter) |
| ILK6 | AT1G14000 | BRL2 | AT2G01950 | Receptor/Signal transduction |
