# Supplementary material for: Insights into the Structure, Function, and Ion-Mediated Signaling Pathways Transduced by Plant Integrin-Linked Kinases
Source: Front Plant Sci. 2017 Apr 3;8:376. doi: 10.3389/fpls.2017.00376 (PMC5376563; doi:10.3389/fpls.2017.00376)
Supplement: DATA S1 — Model and information associated with 3D structure prediction of full length ILK1. [file Data_Sheet_1.ZIP › SDATA_1.ILK1_FullStructure_SupplementalData/hit_report.pdf]

Phyre2

Email	scp319@msstate.edu
Description	Undefined
Date	Tue Jul 19 15:56:00 BST 2016
Unique Job ID	f2321ee0cdfca61b

Detailed template information

#	Template	Alignment Coverage	3D Model	Confidence	% i.d.	Template Information
1	c4oauC_	Alignment	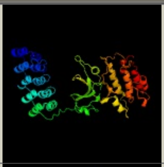	100.0	17	**PDB header:**hydrolase/rna **Chain:** C: **PDB Molecule:**2-5a-dependent ribonuclease; **PDBTitle:** complete human rnase l in complex with biological activators.
2	c2fo0A_	Alignment	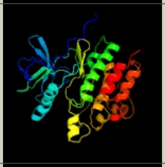	100.0	25	**PDB header:**transferase **Chain:** A: **PDB Molecule:**proto-oncogene tyrosine-protein kinase abl1 (1b isoform); **PDBTitle:** organization of the sh3-sh2 unit in active and inactive forms of the2 c-abl tyrosine kinase
3	c4xi2A_	Alignment	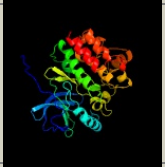	100.0	23	**PDB header:**transferase **Chain:** A: **PDB Molecule:**tyrosine-protein kinase btk; **PDBTitle:** crystal structure of an auto-inhibited form of bruton's tryrosine2 kinase
4	c1oplA_	Alignment	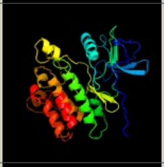	100.0	26	**PDB header:**transferase **Chain:** A: **PDB Molecule:**proto-oncogene tyrosine-protein kinase; **PDBTitle:** structural basis for the auto-inhibition of c-abl tyrosine2 kinase
5	c1y57A_	Alignment	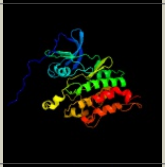	100.0	26	**PDB header:**transferase **Chain:** A: **PDB Molecule:**proto-oncogene tyrosine-protein kinase src; **PDBTitle:** structure of unphosphorylated c-src in complex with an inhibitor
6	c1k9aB_	Alignment	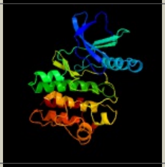	100.0	29	**PDB header:**transferase **Chain:** B: **PDB Molecule:**carboxyl-terminal src kinase; **PDBTitle:** crystal structure analysis of full-length carboxyl-terminal2 src kinase at 2.5 a resolution
7	c2j0kB_	Alignment	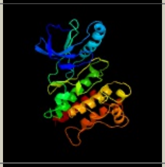	100.0	25	**PDB header:**transferase **Chain:** A: **PDB Molecule:**focal adhesion kinase 1; **PDBTitle:** crystal structure of a fragment of focal adhesion kinase2 containing the ferm and kinase domains.
8	c2h8hA_	Alignment	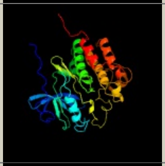	100.0	26	**PDB header:**transferase **Chain:** A: **PDB Molecule:**proto-oncogene tyrosine-protein kinase src; **PDBTitle:** src kinase in complex with a quinazoline inhibitor
9	c4y93A_	Alignment	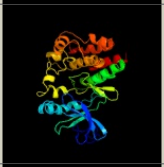	100.0	24	**PDB header:**transferase **Chain:** A: **PDB Molecule:**non-specific protein-tyrosine kinase,non-specific protein- **PDBTitle:** crystal structure of the ph-th-kinase construct of bruton's tyrosine2 kinase (btk)
10	c4fl2A_	Alignment	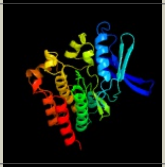	100.0	24	**PDB header:**transferase **Chain:** A: **PDB Molecule:**tyrosine-protein kinase syk; **PDBTitle:** structural and biophysical characterization of the syk activation2 switch
11	c4btfA_	Alignment	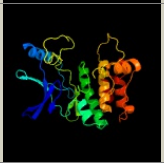	100.0	24	**PDB header:**transferase **Chain:** A: **PDB Molecule:**mixed lineage kinase domain-like protein; **PDBTitle:** structure of mlkl

12	c3qa8H_	Alignment	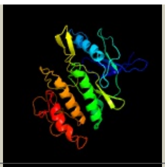	100.0	25	**PDB header:**immune system, signaling protein<br>**Chain:** H: **PDB Molecule:**mgc80376 protein;<br>**PDBTitle:** crystal structure of inhibitor of kappa b kinase beta
13	c2ozoA_	Alignment	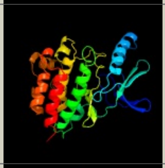	100.0	23	**PDB header:**transferase<br>**Chain:** A: **PDB Molecule:**tyrosine-protein kinase zap-70;<br>**PDBTitle:** autoinhibited intact human zap-70
14	c4im2A_	Alignment	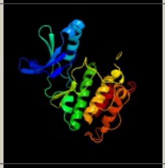	100.0	23	**PDB header:**transferase/transferase inhibitor<br>**Chain:** A: **PDB Molecule:**serine/threonine-protein kinase tbk1;<br>**PDBTitle:** structure of tank-binding kinase 1
15	c2c0iA_	Alignment	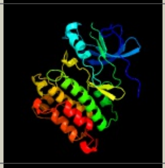	100.0	26	**PDB header:**transferase<br>**Chain:** A: **PDB Molecule:**tyrosine-protein kinase hck;<br>**PDBTitle:** src family kinase hck with bound inhibitor a-420983
16	c3pfqA_	Alignment	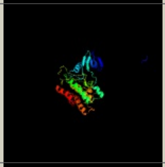	100.0	24	**PDB header:**transferase<br>**Chain:** A: **PDB Molecule:**protein kinase c beta type;<br>**PDBTitle:** crystal structure and allosteric activation of protein kinase c beta2 ii
17	c3qa8A_	Alignment	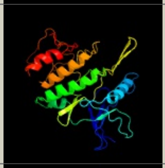	100.0	24	**PDB header:**immune system, signaling protein<br>**Chain:** A: **PDB Molecule:**mgc80376 protein;<br>**PDBTitle:** crystal structure of inhibitor of kappa b kinase beta
18	c3nyoB_	Alignment	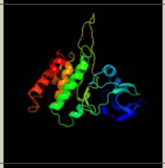	100.0	24	**PDB header:**transferase<br>**Chain:** B: **PDB Molecule:**g protein-coupled receptor kinase 6;<br>**PDBTitle:** crystal structure of g protein-coupled receptor kinase 6 in complex2 with amp
19	c4wnkA_	Alignment	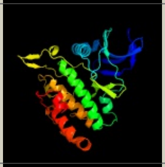	100.0	26	**PDB header:**ligase<br>**Chain:** A: **PDB Molecule:**g protein-coupled receptor kinase 5;<br>**PDBTitle:** crystal structure of bovine g protein coupled-receptor kinase 5 in2 complex with ccg215022
20	c2acxB_	Alignment	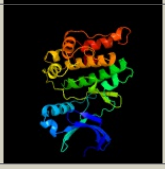	100.0	24	**PDB header:**transferase<br>**Chain:** B: **PDB Molecule:**g protein-coupled receptor kinase 6;<br>**PDBTitle:** crystal structure of g protein coupled receptor kinase 6 bound to2 amppnp
21	c3zzwA_	Alignment	not modelled	100.0	26	**PDB header:**transferase<br>**Chain:** A: **PDB Molecule:**tyrosine-protein kinase transmembrane receptor ror2;<br>**PDBTitle:** crystal structure of the kinase domain of ror2
22	c4czuC_	Alignment	not modelled	100.0	25	**PDB header:**transferase<br>**Chain:** C: **PDB Molecule:**cbl-interacting serine/threonine-protein kinase 23;<br>**PDBTitle:** crystal structure of the kinase domain of snrk3.23 at1g302702 t190d mutant
23	c4c0tA_	Alignment	not modelled	100.0	24	**PDB header:**transferase<br>**Chain:** A: **PDB Molecule:**likely protein kinase;<br>**PDBTitle:** candida albicans pkh kinase domain
24	c3soaA_	Alignment	not modelled	100.0	19	**PDB header:**transferase/transferase inhibitor<br>**Chain:** A: **PDB Molecule:**calcium/calmodulin-dependent protein kinase type ii subunit<br>**PDBTitle:** full-length human camkii
25	c1ym7C_	Alignment	not modelled	100.0	24	**PDB header:**transferase<br>**Chain:** C: **PDB Molecule:**beta-adrenergic receptor kinase 1;<br>**PDBTitle:** g protein-coupled receptor kinase 2 (grk2)
26	d2j4za1	Alignment	not modelled	100.0	23	**Fold:**Protein kinase-like (PK-like)<br>**Superfamily:**Protein kinase-like (PK-like)<br>**Family:**Protein kinases, catalytic subunit
27	c3cblA_	Alignment	not modelled	100.0	27	**PDB header:**transferase<br>**Chain:** A: **PDB Molecule:**proto-oncogene tyrosine-protein kinase fes/fps;<br>**PDBTitle:** crystal structure of human feline sarcoma viral oncogene homologue (v-2 fes) in complex with staurosporine and a consensus peptide
28	c3q5iA_	[illegible]	not modelled	100.0	24	**PDB header:**transferase<br>**Chain:** A: **PDB Molecule:**protein kinase;

						**PDBTitle:** crystal structure of pbanka_031420
29	c4yhjA_	Alignment	not modelled	100.0	23	**PDB header:**transferase<br>**Chain:** A: **PDB Molecule:**g protein-coupled receptor kinase 4;<br>**PDBTitle:** structure and function of the hypertension variant a486v of g protein-2 coupled receptor kinase 4 (grk4)
30	c4i6hA_	Alignment	not modelled	100.0	22	**PDB header:**transferase/transferase inhibitor<br>**Chain:** A: **PDB Molecule:**serine/threonine-protein kinase plk2;<br>**PDBTitle:** selective & brain-permeable polo-like kinase-2 (plk-2) inhibitors that2 reduce alpha-synuclein phosphorylation in rat brain
31	c4dn5A_	Alignment	not modelled	100.0	22	**PDB header:**transferase<br>**Chain:** A: **PDB Molecule:**mitogen-activated protein kinase kinase kinase 14;<br>**PDBTitle:** crystal structure of nf-kb-inducing kinase (nik)
32	c3c4yA_	Alignment	not modelled	100.0	22	**PDB header:**transferase<br>**Chain:** A: **PDB Molecule:**rhodopsin kinase;<br>**PDBTitle:** crystal structure of apo form of g protein coupled receptor kinase 12 at 7.51a
33	c4cfhA_	Alignment	not modelled	100.0	26	**PDB header:**transferase<br>**Chain:** A: **PDB Molecule:**5'-amp-activated protein kinase catalytic subunit alpha-1;<br>**PDBTitle:** structure of an active form of mammalian ampk
34	d1qcfa3	Alignment	not modelled	100.0	25	**Fold:**Protein kinase-like (PK-like)<br>**Superfamily:**Protein kinase-like (PK-like)<br>**Family:**Protein kinases, catalytic subunit
35	c3c4wB_	Alignment	not modelled	100.0	21	**PDB header:**transferase<br>**Chain:** B: **PDB Molecule:**rhodopsin kinase;<br>**PDBTitle:** crystal structure of g protein coupled receptor kinase 1 bound to atp2 and magnesium chloride at 2.7a
36	c4b6lA_	Alignment	not modelled	100.0	20	**PDB header:**transferase<br>**Chain:** A: **PDB Molecule:**serine/threonine-protein kinase plk3;<br>**PDBTitle:** discovery of oral polo-like kinase (plk) inhibitors with enhanced2 selectivity profile using residue targeted drug design
37	c3g0eA_	Alignment	not modelled	100.0	27	**PDB header:**transferase<br>**Chain:** A: **PDB Molecule:**mast/stem cell growth factor receptor;<br>**PDBTitle:** kit kinase domain in complex with sunitinib
38	c2ivsA_	Alignment	not modelled	100.0	30	**PDB header:**transferase<br>**Chain:** A: **PDB Molecule:**proto-oncogene tyrosine-protein kinase receptor<br>**PDBTitle:** crystal structure of non-phosphorylated ret tyrosine kinase2 domain
39	c2vwiC_	Alignment	not modelled	100.0	21	**PDB header:**transferase<br>**Chain:** C: **PDB Molecule:**serine/threonine-protein kinase osr1;<br>**PDBTitle:** structure of the osr1 kinase, a hypertension drug target
40	c1koaA_	Alignment	not modelled	100.0	20	**PDB header:**kinase<br>**Chain:** A: **PDB Molecule:**twitchin;<br>**PDBTitle:** twitchin kinase fragment (c.elegans), autoregulated protein2 kinase and immunoglobulin domains
41	d1opja_	Alignment	not modelled	100.0	27	**Fold:**Protein kinase-like (PK-like)<br>**Superfamily:**Protein kinase-like (PK-like)<br>**Family:**Protein kinases, catalytic subunit
42	c4wb7B_	Alignment	not modelled	100.0	25	**PDB header:**transferase/transferase inhibitor<br>**Chain:** B: **PDB Molecule:**dnaj homolog subfamily b member 1,camp-dependent protein<br>**PDBTitle:** crystal structure of a chimeric fusion of human dnaj (hsp40) and camp-2 dependent protein kinase a (catalytic alpha subunit)
43	c3ckxA_	Alignment	not modelled	100.0	26	**PDB header:**transferase<br>**Chain:** A: **PDB Molecule:**serine/threonine-protein kinase 24;<br>**PDBTitle:** crystal structure of sterile 20-like kinase 3 (mst3, stk24)2 in complex with staurosporine
44	c4oliA_	Alignment	not modelled	100.0	28	**PDB header:**transferase<br>**Chain:** A: **PDB Molecule:**non-receptor tyrosine-protein kinase tyk2;<br>**PDBTitle:** the pseudokinase/kinase protein from jak-family member tyk2
45	c3lijA_	Alignment	not modelled	100.0	21	**PDB header:**transferase<br>**Chain:** A: **PDB Molecule:**calcium/calmodulin dependent protein kinase with<br>**PDBTitle:** crystal structure of full length cpcdpk3 (cgd5_820) in2 complex with ca2+ and amppnp
46	c4y83B_	Alignment	not modelled	100.0	20	**PDB header:**transferase<br>**Chain:** B: **PDB Molecule:**mitogen-activated protein kinase kinase kinase 8;<br>**PDBTitle:** crystal structure of cot kinase domain in complex with 5-(2-amino-5-2 (quinolin-3-yl)pyridin-3-yl)-1,3,4-oxadiazole-2(3h)-thione
47	c4wnoA_	Alignment	not modelled	100.0	27	**PDB header:**transferase/transferase inhibitor<br>**Chain:** A: **PDB Molecule:**serine/threonine-protein kinase ulk1;<br>**PDBTitle:** structure of ulk1 bound to an inhibitor
48	d1u59a_	Alignment	not modelled	100.0	22	**Fold:**Protein kinase-like (PK-like)<br>**Superfamily:**Protein kinase-like (PK-like)<br>**Family:**Protein kinases, catalytic subunit
49	c2c30A_	Alignment	not modelled	100.0	22	**PDB header:**transferase<br>**Chain:** A: **PDB Molecule:**serine/threonine-protein kinase pak 6;<br>**PDBTitle:** crystal structure of the human p21-activated kinase 6
50	c3tkuB_	Alignment	not modelled	100.0	21	**PDB header:**transferase/transferase inhibitor<br>**Chain:** B: **PDB Molecule:**serine/threonine-protein kinase mrck beta;<br>**PDBTitle:** mrck beta in complex with fasudil
51	c4cdsA_	Alignment	not modelled	100.0	29	**PDB header:**transferase<br>**Chain:** A: **PDB Molecule:**tyrosine kinase as - a common ancestor of src and abl;<br>**PDBTitle:** tyrosine kinase as - a common ancestor of src and abl
52	c1lufA_	Alignment	not modelled	100.0	25	**PDB header:**transferase<br>**Chain:** A: **PDB Molecule:**muscle-specific tyrosine kinase receptor musk;<br>**PDBTitle:** crystal structure of the musk tyrosine kinase: insights2 into receptor autoregulation
53	d1lufa_	Alignment	not modelled	100.0	25	**Fold:**Protein kinase-like (PK-like)<br>**Superfamily:**Protein kinase-like (PK-like)

						**Family:**Protein kinases, catalytic subunit
54	d1k2pa_	Alignment	not modelled	100.0	24	**Fold:**Protein kinase-like (PK-like)<br>**Superfamily:**Protein kinase-like (PK-like)<br>**Family:**Protein kinases, catalytic subunit
55	d1qpca_	Alignment	not modelled	100.0	27	**Fold:**Protein kinase-like (PK-like)<br>**Superfamily:**Protein kinase-like (PK-like)<br>**Family:**Protein kinases, catalytic subunit
56	c3dpkA_	Alignment	not modelled	100.0	27	**PDB header:**transferase<br>**Chain:** A: **PDB Molecule:**macrophage colony-stimulating factor 1 receptor;<br>**PDBTitle:** cfms tyrosine kinase in complex with a pyridopyrimidinone2 inhibitor
57	c4myiA_	Alignment	not modelled	100.0	26	**PDB header:**transferase<br>**Chain:** A: **PDB Molecule:**cgmp-dependent protein kinase, putative;<br>**PDBTitle:** crystal structure of pvx_084705
58	c2bujA_	Alignment	not modelled	100.0	20	**PDB header:**transferase<br>**Chain:** A: **PDB Molecule:**serine/threonine-protein kinase 16;<br>**PDBTitle:** crystal structure of the human serine-threonine kinase 162 in complex with staurosporine
59	c2jamB_	Alignment	not modelled	100.0	22	**PDB header:**transferase<br>**Chain:** B: **PDB Molecule:**calcium/calmodulin-dependent protein kinase type<br>**PDBTitle:** crystal structure of human calmodulin-dependent protein2 kinase i g
60	c5ig1A_	Alignment	not modelled	100.0	24	**PDB header:**transferase<br>**Chain:** A: **PDB Molecule:**camk/camk2 protein kinase;<br>**PDBTitle:** crystal structure of s. rosetta camkii kinase domain
61	c2pvfA_	Alignment	not modelled	100.0	29	**PDB header:**transferase<br>**Chain:** A: **PDB Molecule:**fibroblast growth factor receptor 2;<br>**PDBTitle:** crystal structure of tyrosine phosphorylated activated fgf receptor 22 (fgfr2) kinase domain in complex with atp analog and substrate3 peptide
62	c3fbvL_	Alignment	not modelled	100.0	25	**PDB header:**transferase, hydrolase<br>**Chain:** L: **PDB Molecule:**serine/threonine-protein kinase/endoribonuclease ire1;<br>**PDBTitle:** crystal structure of the oligomer formed by the kinase-ribonuclease2 domain of ire1
63	c5ceqA_	Alignment	not modelled	100.0	31	**PDB header:**transferase/transferase inhibitor<br>**Chain:** A: **PDB Molecule:**mitogen-activated protein kinase kinase kinase 12;<br>**PDBTitle:** dlk in complex with inhibitor 2-((1-cyclopentyl-5-(1-(oxetan-3-yl)2 piperidin-4-yl)-1h-pyrazol-3-yl)amino)isonicotinonitrile
64	c2psqA_	Alignment	not modelled	100.0	27	**PDB header:**transferase<br>**Chain:** A: **PDB Molecule:**fibroblast growth factor receptor 2;<br>**PDBTitle:** crystal structure of unphosphorylated unactivated wild type2 fgf receptor 2 (fgfr2) kinase domain
65	d2psqa1	Alignment	not modelled	100.0	27	**Fold:**Protein kinase-like (PK-like)<br>**Superfamily:**Protein kinase-like (PK-like)<br>**Family:**Protein kinases, catalytic subunit
66	c4fijA_	Alignment	not modelled	100.0	21	**PDB header:**transferase<br>**Chain:** A: **PDB Molecule:**serine/threonine-protein kinase pak 4;<br>**PDBTitle:** catalytic domain of human pak4
67	c2bmcD_	Alignment	not modelled	100.0	26	**PDB header:**transferase<br>**Chain:** D: **PDB Molecule:**serine threonine-protein kinase 6;<br>**PDBTitle:** aurora-2 t287d t288d complexed with pha-680632
68	c3lxkA_	Alignment	not modelled	100.0	29	**PDB header:**transferase<br>**Chain:** A: **PDB Molecule:**tyrosine-protein kinase jak3;<br>**PDBTitle:** structural and thermodynamic characterization of the tyk2 and jak32 kinase domains in complex with cp-690550 and cmp-6
69	c2wqmA_	Alignment	not modelled	100.0	21	**PDB header:**transferase<br>**Chain:** A: **PDB Molecule:**serine/threonine-protein kinase nek7;<br>**PDBTitle:** structure of apo human nek7
70	c4xhgA_	Alignment	not modelled	100.0	14	**PDB header:**transferase<br>**Chain:** A: **PDB Molecule:**similar to uniprot p29295 saccharomyces cerevisiae ypl204w
71	d1phka_	Alignment	not modelled	100.0	27	**Fold:**Protein kinase-like (PK-like)<br>**Superfamily:**Protein kinase-like (PK-like)<br>**Family:**Protein kinases, catalytic subunit
72	d1t46a_	Alignment	not modelled	100.0	27	**Fold:**Protein kinase-like (PK-like)<br>**Superfamily:**Protein kinase-like (PK-like)<br>**Family:**Protein kinases, catalytic subunit
73	c1mruB_	Alignment	not modelled	100.0	25	**PDB header:**transferase<br>**Chain:** B: **PDB Molecule:**probable serine/threonine-protein kinase pknb;<br>**PDBTitle:** intracellular ser/thr protein kinase domain of2 mycobacterium tuberculosis pknb.
74	d1yhwa1	Alignment	not modelled	100.0	18	**Fold:**Protein kinase-like (PK-like)<br>**Superfamily:**Protein kinase-like (PK-like)<br>**Family:**Protein kinases, catalytic subunit
75	d1o6ya_	Alignment	not modelled	100.0	25	**Fold:**Protein kinase-like (PK-like)<br>**Superfamily:**Protein kinase-like (PK-like)<br>**Family:**Protein kinases, catalytic subunit
76	c4eqmE_	Alignment	not modelled	100.0	25	**PDB header:**transferase<br>**Chain:** E: **PDB Molecule:**protein kinase;<br>**PDBTitle:** structural analysis of staphylococcus aureus serine/threonine kinase2 pknb
77	c4fieB_	Alignment	not modelled	100.0	21	**PDB header:**transferase<br>**Chain:** B: **PDB Molecule:**serine/threonine-protein kinase pak 4;<br>**PDBTitle:** full-length human pak4
78	d1uwha_	Alignment	not modelled	100.0	26	**Fold:**Protein kinase-like (PK-like)<br>**Superfamily:**Protein kinase-like (PK-like)<br>**Family:**Protein kinases, catalytic subunit

79	c4uy9A_	Alignment	not modelled	100.0	28	**PDB header:**transferase<br>**Chain:** A: **PDB Molecule:**mitogen-activated protein kinase kinase kinase 9;<br>**PDBTitle:** structure of mlk1 kinase domain with leucine zipper 1
80	c3hztA_	Alignment	not modelled	100.0	25	**PDB header:**transferase<br>**Chain:** A: **PDB Molecule:**calcium-dependent protein kinase 3;<br>**PDBTitle:** crystal structure of toxoplasma gondii cdpk3, tgme49_105860
81	c2y7jB_	Alignment	not modelled	100.0	25	**PDB header:**transferase<br>**Chain:** B: **PDB Molecule:**phosphorylase b kinase gamma catalytic chain,<br>**PDBTitle:** structure of human phosphorylase kinase, gamma 2
82	c4ithB_	Alignment	not modelled	100.0	26	**PDB header:**transferase/transferase inhibitor<br>**Chain:** B: **PDB Molecule:**receptor-interacting serine/threonine-protein kinase 1;<br>**PDBTitle:** crystal structure of rip1 kinase in complex with necrostatin-1 analog
83	c3plsA_	Alignment	not modelled	100.0	25	**PDB header:**transferase<br>**Chain:** A: **PDB Molecule:**macrophage-stimulating protein receptor;<br>**PDBTitle:** ron in complex with ligand amp-pnp
84	c3dfaA_	Alignment	not modelled	100.0	25	**PDB header:**transferase<br>**Chain:** A: **PDB Molecule:**calcium-dependent protein kinase cgd3_920;<br>**PDBTitle:** crystal structure of kinase domain of calcium-dependent protein kinase2 cgd3_920 from cryptosporidium parvum
85	c4btjB_	Alignment	not modelled	100.0	16	**PDB header:**transferase<br>**Chain:** B: **PDB Molecule:**tau-tubulin kinase 1;<br>**PDBTitle:** ttbk1 in complex with atp
86	d1fgka_	Alignment	not modelled	100.0	32	**Fold:**Protein kinase-like (PK-like)<br>**Superfamily:**Protein kinase-like (PK-like)<br>**Family:**Protein kinases, catalytic subunit
87	c2wtkC_	Alignment	not modelled	100.0	26	**PDB header:**transferase/metal-binding protein<br>**Chain:** C: **PDB Molecule:**serine/threonine-protein kinase 11;<br>**PDBTitle:** structure of the heterotrimeric lkb1-stradalpha-mo25alpha2 complex
88	c3p23B_	Alignment	not modelled	100.0	26	**PDB header:**hydrolase, transferase<br>**Chain:** B: **PDB Molecule:**serine/threonine-protein kinase/endoribonuclease ire1;<br>**PDBTitle:** crystal structure of the human kinase and rnase domains in complex2 with adp
89	c5dfzB_	Alignment	not modelled	100.0	16	**PDB header:**transferase<br>**Chain:** B: **PDB Molecule:**serine/threonine-protein kinase vps15;<br>**PDBTitle:** structure of vps34 complex ii from s. cerevisiae.
90	c2qg5D_	Alignment	not modelled	100.0	22	**PDB header:**transferase<br>**Chain:** D: **PDB Molecule:**calcium/calmodulin-dependent protein kinase;<br>**PDBTitle:** cryptosporidium parvum calcium dependent protein kinase cgd7_1840
91	c2a27B_	Alignment	not modelled	100.0	25	**PDB header:**transferase<br>**Chain:** B: **PDB Molecule:**death-associated protein kinase 2;<br>**PDBTitle:** human drp-1 kinase, w305s s308a d40 mutant, crystal form with 82 monomers in the asymmetric unit
92	c2pziA_	Alignment	not modelled	100.0	24	**PDB header:**transferase<br>**Chain:** A: **PDB Molecule:**probable serine/threonine-protein kinase pkng;<br>**PDBTitle:** crystal structure of protein kinase pkng from mycobacterium2 tuberculosis in complex with tetrahydrobenzothiophene ax20017
93	c4qfrA_	Alignment	not modelled	100.0	24	**PDB header:**signaling protein/inhibitor/activator<br>**Chain:** A: **PDB Molecule:**5'-amp-activated protein kinase catalytic subunit alpha-1;<br>**PDBTitle:** structure of ampk in complex with cl-a769662 activator and2 staurosporine inhibitor
94	c2a1aB_	Alignment	not modelled	100.0	24	**PDB header:**protein synthesis/transferase<br>**Chain:** B: **PDB Molecule:**interferon-induced, double-stranded rna-activated protein<br>**PDBTitle:** pkr kinase domain-eif2alpha complex
95	d3bu3a1	Alignment	not modelled	100.0	27	**Fold:**Protein kinase-like (PK-like)<br>**Superfamily:**Protein kinase-like (PK-like)<br>**Family:**Protein kinases, catalytic subunit
96	c3d4qA_	Alignment	not modelled	100.0	28	**PDB header:**transferase<br>**Chain:** A: **PDB Molecule:**b-raf proto-oncogene serine/threonine-protein kinase;<br>**PDBTitle:** pyrazole-based inhibitors of b-raf kinase
97	c3kulB_	Alignment	not modelled	100.0	26	**PDB header:**transferase<br>**Chain:** B: **PDB Molecule:**ephrin type-a receptor 8;<br>**PDBTitle:** kinase domain of human ephrin type-a receptor 8 (epha8)
98	c2c47D_	Alignment	not modelled	100.0	13	**PDB header:**transferase<br>**Chain:** D: **PDB Molecule:**casein kinase 1 gamma 2 isoform;<br>**PDBTitle:** structure of casein kinase 1 gamma 2
99	c4aojA_	Alignment	not modelled	100.0	27	**PDB header:**transferase<br>**Chain:** A: **PDB Molecule:**high affinity nerve growth factor receptor;<br>**PDBTitle:** human trka in complex with the inhibitor az-23
100	c1xjdA_	Alignment	not modelled	100.0	25	**PDB header:**transferase<br>**Chain:** A: **PDB Molecule:**protein kinase c, theta type;<br>**PDBTitle:** crystal structure of pkc-theta complexed with staurosporine2 at 2a resolution
101	d1xjda_	Alignment	not modelled	100.0	25	**Fold:**Protein kinase-like (PK-like)<br>**Superfamily:**Protein kinase-like (PK-like)<br>**Family:**Protein kinases, catalytic subunit
102	c4eutA_	Alignment	not modelled	100.0	21	**PDB header:**transferase/transferase inhibitor<br>**Chain:** A: **PDB Molecule:**serine/threonine-protein kinase tbk1;<br>**PDBTitle:** structure of bx-795 complexed with unphosphorylated human tbk1 kinase-2 uld domain
103	[illegible]	[illegible]	not modelled	100.0	22	**PDB header:**transferase<br>**Chain:** E: **PDB Molecule:**nf-kappa-beta-inducing kinase;

103	c4g3d[illegible]_		Alignment	not modelled	100.0	22	[illegible] **PDBTitle:** crystal structure of human nf-kappab inducing kinase (nik)
104	c2bdwB_		Alignment	not modelled	100.0	17	**PDB header:**transferase **Chain:** B: **PDB Molecule:**hypothetical protein k11e8.1d; **PDBTitle:** crystal structure of the auto-inhibited kinase domain of2 calcium/calmodulin activated kinase ii
105	d1jksa_		Alignment	not modelled	100.0	24	**Fold:**Protein kinase-like (PK-like) **Superfamily:**Protein kinase-like (PK-like) **Family:**Protein kinases, catalytic subunit
106	c4ebwA_		Alignment	not modelled	100.0	26	**PDB header:**transferase/transferase inhibitor **Chain:** A: **PDB Molecule:**focal adhesion kinase 1; **PDBTitle:** structure of focal adhesion kinase catalytic domain in complex with2 novel allosteric inhibitor
107	d1sm2a_		Alignment	not modelled	100.0	30	**Fold:**Protein kinase-like (PK-like) **Superfamily:**Protein kinase-like (PK-like) **Family:**Protein kinases, catalytic subunit
108	c2cgvA_		Alignment	not modelled	100.0	23	**PDB header:**transferase **Chain:** A: **PDB Molecule:**serine/threonine-protein kinase chk1; **PDBTitle:** identification of chemically diverse chk1 inhibitors by2 receptor-based virtual screening
109	c2clqA_		Alignment	not modelled	100.0	26	**PDB header:**transferase **Chain:** A: **PDB Molecule:**mitogen-activated protein kinase kinase kinase 5; **PDBTitle:** structure of mitogen-activated protein kinase kinase kinase2 5
110	c4x3fA_		Alignment	not modelled	100.0	27	**PDB header:**transferase **Chain:** A: **PDB Molecule:**serine/threonine-protein kinase pkna; **PDBTitle:** crystal structure of the intracellular domain of the m. tuberculosis2 ser/thr kinase pkna
111	c3d9vA_		Alignment	not modelled	100.0	23	**PDB header:**transferase **Chain:** A: **PDB Molecule:**rho-associated protein kinase 1; **PDBTitle:** crystal structure of rock i bound to h-1152p a di-2 methylated variant of fasudil
112	c3comB_		Alignment	not modelled	100.0	22	**PDB header:**transferase **Chain:** B: **PDB Molecule:**serine/threonine-protein kinase 4; **PDBTitle:** crystal structure of mst1 kinase
113	c2j51A_		Alignment	not modelled	100.0	24	**PDB header:**transferase **Chain:** A: **PDB Molecule:**ste20-like serine/threonine-protein kinase; **PDBTitle:** crystal structure of human ste20-like kinase bound to 5-2 amino-3-((4-(aminosulfonyl)phenyl)amino)-n-(2,6-3 difluorophenyl)-1h-1,2,4-triazole-1-carbothioamide
114	c1yvjA_		Alignment	not modelled	100.0	29	**PDB header:**transferase **Chain:** A: **PDB Molecule:**tyrosine-protein kinase jak3; **PDBTitle:** crystal structure of the jak3 kinase domain in complex with2 a staurosporine analogue
115	c3vw6B_		Alignment	not modelled	100.0	28	**PDB header:**transferase/transferase inhibitor **Chain:** B: **PDB Molecule:**mitogen-activated protein kinase kinase kinase 5; **PDBTitle:** crystal structure of human apoptosis signal-regulating kinase 1 (ask1)2 with imidazopyridine inhibitor
116	d1xbba_		Alignment	not modelled	100.0	23	**Fold:**Protein kinase-like (PK-like) **Superfamily:**Protein kinase-like (PK-like) **Family:**Protein kinases, catalytic subunit
117	c4lgdB_		Alignment	not modelled	100.0	21	**PDB header:**signaling protein **Chain:** B: **PDB Molecule:**serine/threonine-protein kinase 3; **PDBTitle:** structural basis for autoactivation of human mst2 kinase and its2 regulation by rassf5
118	d1nvra_		Alignment	not modelled	100.0	24	**Fold:**Protein kinase-like (PK-like) **Superfamily:**Protein kinase-like (PK-like) **Family:**Protein kinases, catalytic subunit
119	c3l9pA_		Alignment	not modelled	100.0	27	**PDB header:**transferase **Chain:** A: **PDB Molecule:**anaplastic lymphoma kinase; **PDBTitle:** crystal structure of the anaplastic lymphoma kinase catalytic domain
120	c2ya9A_		Alignment	not modelled	100.0	25	**PDB header:**transferase **Chain:** A: **PDB Molecule:**death-associated protein kinase 2; **PDBTitle:** crystal structure of the autoinhibited form of mouse dapk2
