# Supplementary material for: Insights into the Structure, Function, and Ion-Mediated Signaling Pathways Transduced by Plant Integrin-Linked Kinases
Source: Front Plant Sci. 2017 Apr 3;8:376. doi: 10.3389/fpls.2017.00376 (PMC5376563; doi:10.3389/fpls.2017.00376)
Supplement: DATA S1 — Model and information associated with 3D structure prediction of full length ILK1. [file Data_Sheet_1.ZIP › SDATA_1.ILK1_FullStructure_SupplementalData/ss_report.pdf]

Email	scp319@msstate.edu
Description	Undefined
Date	Tue Jul 19 15:56:00 BST 2016
Unique Job ID	f2321ee0cdfca61b

Phyre²

Secondary structure and disorder prediction

	1 … 10 … 20 … 30 … 40 … 50 … 60
Sequence	M E N I T A Q L K R G I S R Q F S T G S I R R T L S R Q F T R Q S S L D P R R T N M R F S F G R Q S S L D P I R R S P D
Secondary structure	
SS confidence	
Disorder	? ?
Disorder confidence	
Conserved Domain info	

	… 70 … 80 … 90 … 100 … 110 … 120
Sequence	S S K S D D E P H M S V P E N L D S T M Q L L F M A S K G D V R G I E E L L D E G I D V N S I D L D G R T A L H I A A C
Secondary structure	
SS confidence	
Disorder	? ?
Disorder confidence	
Conserved Domain info	

	… 130 … 140 … 150 … 160 … 170 … 180
Sequence	E G H L G V V K A L L S R R A N I D A R D R W G S T A A A D A K Y Y G N L D V Y N L L K A R G A K V P K T R K T P M T V
Secondary structure	
SS confidence	
Disorder	? ?
Disorder confidence	
Conserved Domain info	

	… 190 … 200 … 210 … 220 … 230 … 240
Sequence	S N P R E V P E Y E L N P L E V Q V R K S D G I S K G A Y Q V A K W N G T R V S V K I L D K D S Y S D P E R I N A F R H
Secondary structure	
SS confidence	
Disorder	? ? ? ? ? ? ? ? ? ? ? ? ? ? ? ? ? ? ?
Disorder confidence	
Conserved Domain info	• • • • • • •

	… 250 … 260 … 270 … 280 … 290 … 300
Sequence	E L T L L E K V R H P N V I Q F V G A V T Q N I P M M I V V E Y N P K G D L S V Y L Q K K G R L S P S K A L R F A L D I
Secondary structure	
SS confidence	
Disorder	? ?
Disorder confidence	
Conserved Domain info	• • • • •

	… 310 … 320 … 330 … 340 … 350 … 360
Sequence	A R G M N Y L H E C K P D P I I H C D L K P K N I L L D R G G Q L K I S G F G M I R L S K I S Q D K A K V A N H K A H I
Secondary structure	

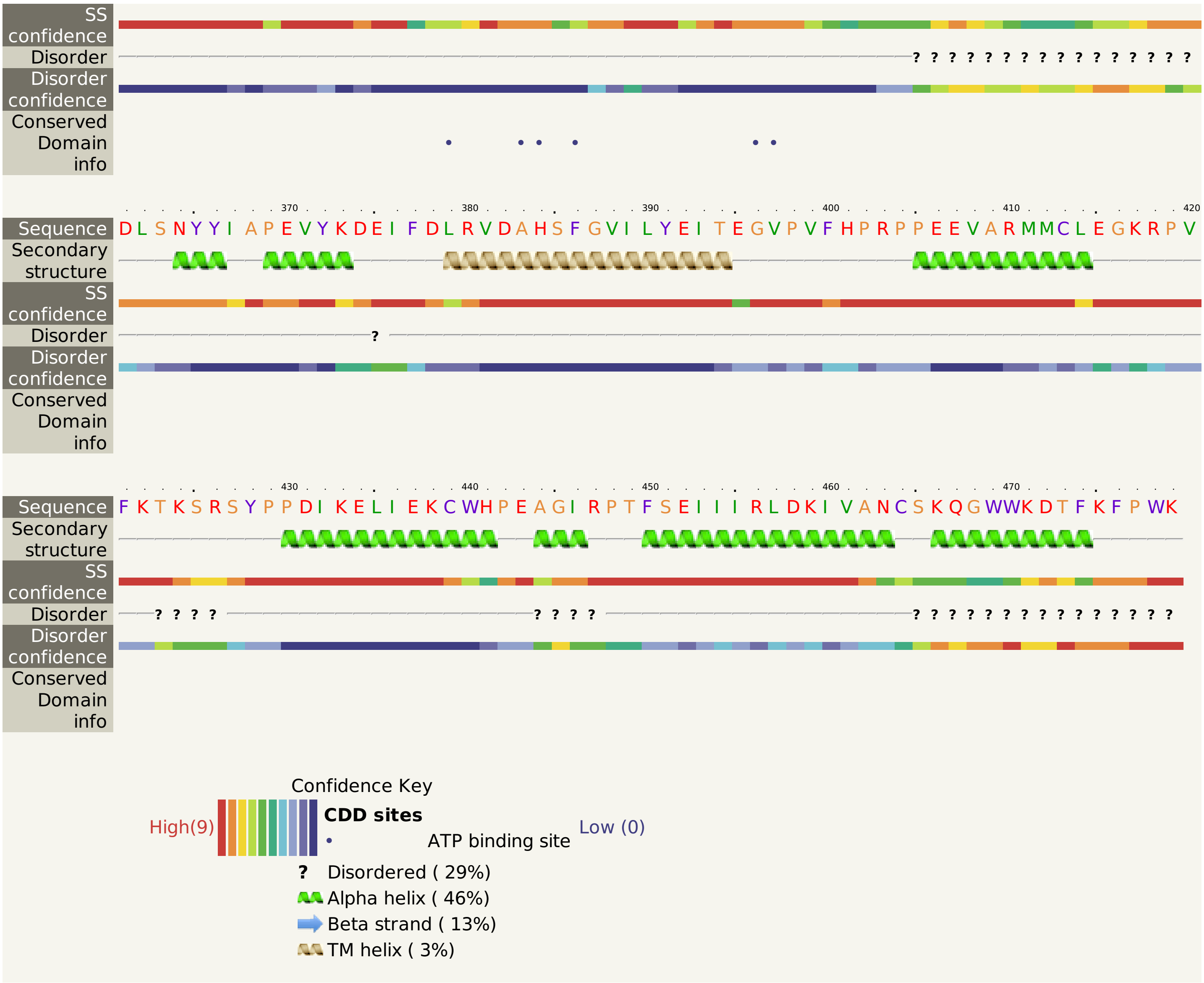
SS confidence
Disorder
Disorder confidence
Conserved Domain info
370
380
390
400
410
420
Sequence
DLSNYYIAPEVYKDEIFDLRVDAHSFGVILYEITEGVPVFHPRPPEEVARMMCLEGKRPV
Secondary structure
SS confidence
Disorder
Disorder confidence
Conserved Domain info
430
440
450
460
470
Sequence
FKTKSRSYPPDIKELIEKCWHPEAGIRPTFSEIIIRLDKIVANCSKQGWWKDTFKFPWK
Secondary structure
SS confidence
Disorder
Disorder confidence
Conserved Domain info
Confidence Key
High(9)
Low (0)
CDD sites
ATP binding site
Disordered (29%)
Alpha helix (46%)
Beta strand (13%)
TM helix (3%)
