# Supplementary material for: Insights into the Structure, Function, and Ion-Mediated Signaling Pathways Transduced by Plant Integrin-Linked Kinases
Source: Front Plant Sci. 2017 Apr 3;8:376. doi: 10.3389/fpls.2017.00376 (PMC5376563; doi:10.3389/fpls.2017.00376)
Supplement: DATA S2 — Model and information associated with 3D structure prediction of ankyrin repeat domains of ILK1 to ILK6. [file Data_Sheet_2.ZIP › SDATA_2_ILKs_AR_SupplementalData/ILK1_ss_report.pdf]

Email	scp319@msstate.edu
Description	ILK1AR___
Date	Fri Jul 29 14:39:49 BST 2016
Unique Job ID	03394cf9414d5632

Secondary structure and disorder prediction

1 . . . . 10 . . . . 20 . . . . 30 . . . . 40 . . . . 50 60

Sequence M E N I T A Q L K R G I S R Q F S T G S I R R T L S R Q F T R Q S S L D P R R T N M R F S F G R Q S S L D P I R R S P D

Secondary structure

SS confidence

Disorder ?

Disorder confidence

Conserved Domain info

. . . . 70 . . . . 80 . . . . 90 . . . . 100 . . . . 110 120

Sequence S S K S D D E P H M S V P E N L D S T M Q L L F M A S K G D V R G I E E L L D E G I D V N S I D L D G R T A L H I A A C

Secondary structure

SS confidence

Disorder ? ? ? ? ? ? ? ? ? ? ? ? ? ?

Disorder confidence

Conserved Domain info

. . . . 130 . . . . 140 . . . . 150 . . . . 160 . . . . 170 180

Sequence E G H L G V V K A L L S R R A N I D A R D R W G S T A A A D A K Y Y G N L D V Y N L L K A R G A K V P K T R K T P M T V

Secondary structure

SS confidence

Disorder ? ? ? ?

Disorder confidence

Conserved Domain info

. . . . 190 . . . .

Sequence S N P R E V P E Y E L N P L E V Q V

Secondary structure

SS confidence

Disorder ? ? ?

Disorder confidence

Conserved Domain info

Confidence Key

High(9) Low (0)

? Disordered (20%)

Alpha helix (59%)

Beta strand (0%)
