# Supplementary material for: Insights into the Structure, Function, and Ion-Mediated Signaling Pathways Transduced by Plant Integrin-Linked Kinases
Source: Front Plant Sci. 2017 Apr 3;8:376. doi: 10.3389/fpls.2017.00376 (PMC5376563; doi:10.3389/fpls.2017.00376)
Supplement: DATA S2 — Model and information associated with 3D structure prediction of ankyrin repeat domains of ILK1 to ILK6. [file Data_Sheet_2.ZIP › SDATA_2_ILKs_AR_SupplementalData/ILK2_ss_report.pdf]

Phyre2

Email	scp319@msstate.edu
Description	ILK2_AR__
Date	Fri Jul 29 15:30:41 BST 2016
Unique Job ID	5f972ac4a430bac5

Secondary structure and disorder prediction

Sequence (1–60): M D N I A A Q L K R G I S R Q F S T G S M R R T L S R Q F T R Q N S L D P R R N N M R F S F G R Q S S L D P I R R S P E

Disorder: ? ? ? ? ? ? ? ? ? ? ? ? ? ? ? ? ? ? ?

Sequence (61–120): S L S C E P H M S V P E N L D S T M Q L L F M A S K G D V N G V E E L L N E G I D V N S I D L D G R T A L H I A S C E G

Disorder: ? ? ?

Sequence (121–180): H Y D V V K V L L S R R A N I D A R D R W G S T A A V D A K Y Y G N V E V Y N L L K A R G A K A P K T R K T P M T V G N

Disorder: ? ? ? ?

Sequence (181–196): P K E V P E Y E L N P L E L Q V

Disorder: ? ? ?

Confidence Key

High(9) … Low (0)

? Disordered (14%)

Alpha helix (51%)

Beta strand (0%)
