# Supplementary material for: Insights into the Structure, Function, and Ion-Mediated Signaling Pathways Transduced by Plant Integrin-Linked Kinases
Source: Front Plant Sci. 2017 Apr 3;8:376. doi: 10.3389/fpls.2017.00376 (PMC5376563; doi:10.3389/fpls.2017.00376)
Supplement: DATA S2 — Model and information associated with 3D structure prediction of ankyrin repeat domains of ILK1 to ILK6. [file Data_Sheet_2.ZIP › SDATA_2_ILKs_AR_SupplementalData/ILK3_ss_report.pdf]

Email	scp319@msstate.edu
Description	ILK3_AR__
Date	Fri Jul 29 15:31:27 BST 2016
Unique Job ID	eccc3fe583367fe3

Secondary structure and disorder prediction

1 . . . . 10 . . . . 20 . . . . 30 . . . . 40 . . . . 50 60

Sequence: M A N V V G Q L K R G I S R Q F S T G S L R R T L S R Q F T R Q A S H D P R R N N M R F S F G R Q S S L D P I R R S P D

Secondary structure

SS confidence

Disorder: ? ? ? ? ? ? ? ? ? ? ? ? ? ? ?

Disorder confidence

Conserved Domain info

. . . . 70 . . . . 80 . . . . 90 . . . . 100 . . . . 110 120

Sequence: G S N G P Q L A V P D N L D A T M Q L L F V A C R G D V E G V Q D L L D E G I D V N S I D L D G R T A L H I A A C E G H

Secondary structure

SS confidence

Disorder: ?

Disorder confidence

Conserved Domain info

. . . . 130 . . . . 140 . . . . 150 . . . . 160 . . . . 170 180

Sequence: V D V V K L L L T R K A N I D A R D R W G S T A A A D A K Y Y G N M D V F N I L K A R G A K V P K T K R T P M V V A N P

Secondary structure

SS confidence

Disorder: ? ? ?

Disorder confidence

Conserved Domain info

. . . . 190 . . . .

Sequence: R E V P E Y E L N P Q E L Q V R K A

Secondary structure

SS confidence

Disorder: ? ? ? ? ? ?

Disorder confidence

Conserved Domain info

Confidence Key

High(9) Low (0)

? Disordered (13%)

Alpha helix (55%)

Beta strand (3%)
