# Supplementary material for: Insights into the Structure, Function, and Ion-Mediated Signaling Pathways Transduced by Plant Integrin-Linked Kinases
Source: Front Plant Sci. 2017 Apr 3;8:376. doi: 10.3389/fpls.2017.00376 (PMC5376563; doi:10.3389/fpls.2017.00376)
Supplement: DATA S2 — Model and information associated with 3D structure prediction of ankyrin repeat domains of ILK1 to ILK6. [file Data_Sheet_2.ZIP › SDATA_2_ILKs_AR_SupplementalData/ILK4_hit_report.pdf]

Phyre2

| | |
|---|---|
| Email | scp319@msstate.edu |
| Description | ILK4_AR__ |
| Date | Fri Jul 29 15:31:48 BST 2016 |
| Unique Job ID | 941274f5f6a2028c |

Detailed template information

| # | Template | Alignment Coverage | 3D Model | Confidence | % i.d. | Template Information |
|---|---|---|---|---|---|---|
| 1 | c4rlvA_ | Alignment | 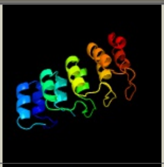 | 100.0 | 23 | **PDB header:**structural protein **Chain:** A: **PDB Molecule:**ankyrin-1, ankyrin-2; **PDBTitle:** crystal structure of ankb 24 ankyrin repeats in complex with ankr2 autoinhibition segment |
| 2 | c4cj9A_ | Alignment | 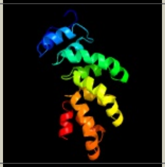 | 100.0 | 9 | **PDB header:**transcription **Chain:** A: **PDB Molecule:**burrh; **PDBTitle:** burrh dna-binding protein from burkholderia rhizoxinica in2 its apo form |
| 3 | c4xd0A_ | Alignment | 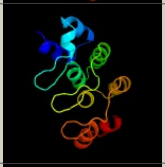 | 100.0 | 18 | **PDB header:**transferase **Chain:** A: **PDB Molecule:**tdp-3-aminoquinovose-n-formyltransferase; **PDBTitle:** x-ray structure of the n-formyltransferase qdtf from providencia2 alcalifaciens |
| 4 | c4oauC_ | Alignment | 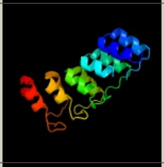 | 100.0 | 23 | **PDB header:**hydrolase/rna **Chain:** C: **PDB Molecule:**2-5a-dependent ribonuclease; **PDBTitle:** complete human rnase l in complex with biological activators. |
| 5 | c1n11A_ | Alignment | 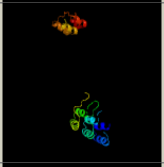 | 100.0 | 24 | **PDB header:**structural protein **Chain:** A: **PDB Molecule:**ankyrin; **PDBTitle:** d34 region of human ankyrin-r and linker |
| 6 | d1n11a_ | Alignment | 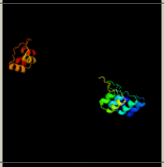 | 100.0 | 24 | **Fold:**beta-hairpin-alpha-hairpin repeat **Superfamily:**Ankyrin repeat **Family:**Ankyrin repeat |
| 7 | c1ycsB_ | Alignment | 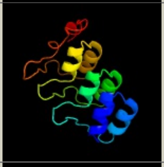 | 100.0 | 29 | **PDB header:**complex (anti-oncogene/ankyrin repeats) **Chain:** B: **PDB Molecule:**53bp2; **PDBTitle:** p53-53bp2 complex |
| 8 | c3j9pD_ | Alignment | 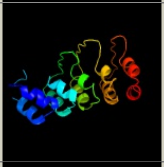 | 100.0 | 20 | **PDB header:**transport protein **Chain:** D: **PDB Molecule:**maltose-binding periplasmic protein, transient receptor **PDBTitle:** structure of the trpa1 ion channel determined by electron cryo-2 microscopy |
| 9 | c3keaB_ | Alignment | 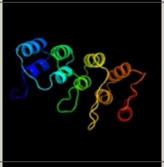 | 100.0 | 16 | **PDB header:**viral protein **Chain:** B: **PDB Molecule:**k1l; **PDBTitle:** structure function studies of vaccinia virus host-range protein k12 reveal a novel ankyrin repeat interaction surface for k1s function |
| 10 | c3j5pB_ | Alignment | 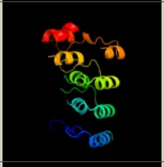 | 100.0 | 20 | **PDB header:**transport protein **Chain:** B: **PDB Molecule:**transient receptor potential cation channel subfamily v **PDBTitle:** structure of trpv1 ion channel determined by single particle electron2 cryo-microscopy |
| 11 | c1sw6A_ | Alignment | 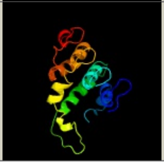 | 99.9 | 12 | **PDB header:**transcription regulation **Chain:** A: **PDB Molecule:**regulatory protein swi6; **PDBTitle:** s. cerevisiae swi6 ankyrin-repeat fragment |

| | | | | | | |
|---|---|---|---|---|---|---|
| 12 | d1sw6a_ | Alignment | 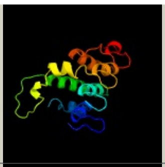 | 99.9 | 12 | **Fold:**beta-hairpin-alpha-hairpin repeat<br>**Superfamily:**Ankyrin repeat<br>**Family:**Ankyrin repeat |
| 13 | c3jueA_ | Alignment | 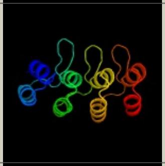 | 99.9 | 18 | **PDB header:**protein transport/endocytosis<br>**Chain:** A: **PDB Molecule:**arfgap with coiled-coil, ank repeat and ph domain-<br>**PDBTitle:** crystal structure of arfgap and ank repeat domain of acap1 |
| 14 | c2ajaA_ | Alignment | 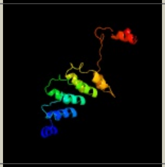 | 99.9 | 14 | **PDB header:**protein binding<br>**Chain:** A: **PDB Molecule:**ankyrin repeat family protein;<br>**PDBTitle:** x-ray structure of an ankyrin repeat family protein q5zsv02 from legionella pneumophila. northeast structural genomics3 consortium target lgr21. |
| 15 | c3t9kA_ | Alignment | 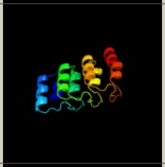 | 99.9 | 18 | **PDB header:**protein transport<br>**Chain:** A: **PDB Molecule:**arf-gap with coiled-coil, ank repeat and ph domain-<br>**PDBTitle:** crystal structure of acap1 c-portion mutant s554d fused with integrin2 beta1 peptide |
| 16 | d2ajaa1 | Alignment | 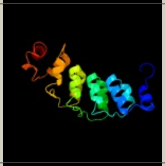 | 99.9 | 14 | **Fold:**alpha-alpha superhelix<br>**Superfamily:**Pseudo ankyrin repeat-like<br>**Family:**Pseudo ankyrin repeat |
| 17 | c3utmA_ | Alignment | 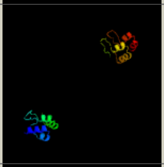 | 99.9 | 40 | **PDB header:**transferase/signaling protein<br>**Chain:** A: **PDB Molecule:**tankyrase-1;<br>**PDBTitle:** crystal structure of a mouse tankyrase-axin complex |
| 18 | c2b0oF_ | Alignment | 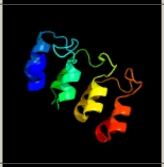 | 99.9 | 21 | **PDB header:**metal binding protein<br>**Chain:** F: **PDB Molecule:**uplc1;<br>**PDBTitle:** crystal structure of uplc1 gap domain |
| 19 | c2fo1E_ | Alignment | 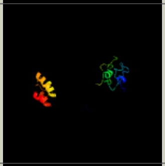 | 99.9 | 20 | **PDB header:**gene regulation/signalling protein/dna<br>**Chain:** E: **PDB Molecule:**lin-12 protein;<br>**PDBTitle:** crystal structure of the csl-notch-mastermind ternary2 complex bound to dna |
| 20 | c4bepB_ | Alignment | 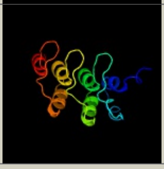 | 99.9 | 19 | **PDB header:**transferase<br>**Chain:** B: **PDB Molecule:**phosphocholine transferase ankx;<br>**PDBTitle:** crystal structure of the legionella pneumophila fic domain-2 containing effector ankx protein (apo-form) |
| 21 | d2fo1e1 | Alignment | not modelled | 99.9 | 21 | **Fold:**beta-hairpin-alpha-hairpin repeat<br>**Superfamily:**Ankyrin repeat<br>**Family:**Ankyrin repeat |
| 22 | c3eu9B_ | Alignment | not modelled | 99.9 | 25 | **PDB header:**protein binding, methyl-lysine-binding p<br>**Chain:** B: **PDB Molecule:**huntingtin-interacting protein 14;<br>**PDBTitle:** the ankyrin repeat domain of huntingtin interacting protein 14 |
| 23 | c4g8kA_ | Alignment | not modelled | 99.9 | 27 | **PDB header:**hydrolase<br>**Chain:** A: **PDB Molecule:**2-5a-dependent ribonuclease;<br>**PDBTitle:** intact sensor domain of human rnase l in the inactive signaling state |
| 24 | c2xaiD_ | Alignment | not modelled | 99.9 | 20 | **PDB header:**transcription<br>**Chain:** D: **PDB Molecule:**ankyrin repeat and socs box protein 9;<br>**PDBTitle:** crystal structure of ankyrin repeat and socs box-containing2 protein 9 (asb9) in complex with elonginb and elonginc |
| 25 | d1uoha_ | Alignment | not modelled | 99.9 | 31 | **Fold:**beta-hairpin-alpha-hairpin repeat<br>**Superfamily:**Ankyrin repeat<br>**Family:**Ankyrin repeat |
| 26 | c3ui2A_ | Alignment | not modelled | 99.9 | 28 | **PDB header:**transport protein<br>**Chain:** A: **PDB Molecule:**signal recognition particle 43 kda protein, chloroplastic;<br>**PDBTitle:** crystal structure of the cpsrp54 tail bound to cpsrp43 |
| 27 | d1dcqa1 | Alignment | not modelled | 99.9 | 23 | **Fold:**beta-hairpin-alpha-hairpin repeat<br>**Superfamily:**Ankyrin repeat<br>**Family:**Ankyrin repeat |
| 28 | c4uucA_ | Alignment | not modelled | 99.9 | 24 | **PDB header:**signaling protein<br>**Chain:** A: **PDB Molecule:**ankyrin repeat and socs box protein 11;<br>**PDBTitle:** crystal structure of human asb11 ankyrin repeat domain |
| | | | | | | **PDB header:**de novo protein |

| | | | | | | |
|---|---|---|---|---|---|---|
| 29 | c4o60A_ | Alignment | not modelled | 99.9 | 28 | **Chain:** A: **PDB Molecule:**ank-n5c-317; **PDBTitle:** structure of ankyrin repeat protein |
| 30 | c1dcqA_ | Alignment | not modelled | 99.9 | 22 | **PDB header:**metal binding protein **Chain:** A: **PDB Molecule:**pyk2-associated protein beta; **PDBTitle:** crystal structure of the arf-gap domain and ankyrin repeats2 of papbeta. |
| 31 | c3t8kB_ | Alignment | not modelled | 99.9 | 13 | **PDB header:**structural genomics, unknown function **Chain:** B: **PDB Molecule:**uncharacterized protein; **PDBTitle:** the crystal structure of a functionally unknown protein lebu_0176 from2 leptotrichia buccalis c-1013-b |
| 32 | c4ot9A_ | Alignment | not modelled | 99.9 | 20 | **PDB header:**transcription **Chain:** A: **PDB Molecule:**nuclear factor nf-kappa-b p100 subunit; **PDBTitle:** crystal structure of the c-terminal domain of p100/nf-kb2 |
| 33 | d1s70b_ | Alignment | not modelled | 99.9 | 24 | **Fold:**beta-hairpin-alpha-hairpin repeat **Superfamily:**Ankyrin repeat **Family:**Ankyrin repeat |
| 34 | c3ehrB_ | Alignment | not modelled | 99.9 | 21 | **PDB header:**signaling protein **Chain:** B: **PDB Molecule:**osteoclast-stimulating factor 1; **PDBTitle:** crystal structure of human osteoclast stimulating factor |
| 35 | d1k1aa_ | Alignment | not modelled | 99.9 | 24 | **Fold:**beta-hairpin-alpha-hairpin repeat **Superfamily:**Ankyrin repeat **Family:**Ankyrin repeat |
| 36 | d1wdya_ | Alignment | not modelled | 99.9 | 24 | **Fold:**beta-hairpin-alpha-hairpin repeat **Superfamily:**Ankyrin repeat **Family:**Ankyrin repeat |
| 37 | d1ot8a_ | Alignment | not modelled | 99.9 | 25 | **Fold:**beta-hairpin-alpha-hairpin repeat **Superfamily:**Ankyrin repeat **Family:**Ankyrin repeat |
| 38 | d1ycsb1 | Alignment | not modelled | 99.9 | 27 | **Fold:**beta-hairpin-alpha-hairpin repeat **Superfamily:**Ankyrin repeat **Family:**Ankyrin repeat |
| 39 | c3deoA_ | Alignment | not modelled | 99.9 | 26 | **PDB header:**protein transport, membrane protein **Chain:** A: **PDB Molecule:**signal recognition particle 43 kda protein; **PDBTitle:** structural basis for specific substrate recognition by the2 chloroplast signal recognition particle protein cpsrp43 |
| 40 | c3d9hA_ | Alignment | not modelled | 99.9 | 21 | **PDB header:**structural protein, protein binding **Chain:** A: **PDB Molecule:**cdna flj77766, highly similar to homo sapiens **PDBTitle:** crystal structure of the splice variant of human asb92 (hasb9-2), an ankyrin repeat protein |
| 41 | c3b7bB_ | Alignment | not modelled | 99.9 | 27 | **PDB header:**transferase **Chain:** B: **PDB Molecule:**euchromatic histone-lysine n-methyltransferase 1; **PDBTitle:** euhmt1 (glp) ankyrin repeat domain (structure 1) |
| 42 | c3lvrE_ | Alignment | not modelled | 99.9 | 22 | **PDB header:**protein transport **Chain:** E: **PDB Molecule:**arf-gap with sh3 domain, ank repeat and ph domain- **PDBTitle:** the crystal structure of asap3 in complex with arf6 in transition2 state soaked with calcium |
| 43 | c2vgeA_ | Alignment | not modelled | 99.9 | 24 | **PDB header:**transcription **Chain:** A: **PDB Molecule:**rela-associated inhibitor; **PDBTitle:** crystal structure of the c-terminal region of human iaspp |
| 44 | c4hbdA_ | Alignment | not modelled | 99.9 | 27 | **PDB header:**protein binding **Chain:** A: **PDB Molecule:**kn motif and ankyrin repeat domain-containing protein 2; **PDBTitle:** crystal structure of kank2 ankyrin repeats |
| 45 | d1myoa_ | Alignment | not modelled | 99.9 | 29 | **Fold:**beta-hairpin-alpha-hairpin repeat **Superfamily:**Ankyrin repeat **Family:**Ankyrin repeat |
| 46 | d1iknd_ | Alignment | not modelled | 99.9 | 19 | **Fold:**beta-hairpin-alpha-hairpin repeat **Superfamily:**Ankyrin repeat **Family:**Ankyrin repeat |
| 47 | c2f8xK_ | Alignment | not modelled | 99.9 | 25 | **PDB header:**transcription/dna **Chain:** K: **PDB Molecule:**neurogenic locus notch homolog protein 1; **PDBTitle:** crystal structure of activated notch, csl and maml on hes-12 promoter dna sequence |
| 48 | d1ixva_ | Alignment | not modelled | 99.9 | 25 | **Fold:**beta-hairpin-alpha-hairpin repeat **Superfamily:**Ankyrin repeat **Family:**Ankyrin repeat |
| 49 | c3hraA_ | Alignment | not modelled | 99.8 | 19 | **PDB header:**structural protein **Chain:** A: **PDB Molecule:**ankyrin repeat family protein; **PDBTitle:** crystal structure of ef0377 an ankyrin repeat protein |
| 50 | c3ljnA_ | Alignment | not modelled | 99.8 | 17 | **PDB header:**unknown function **Chain:** A: **PDB Molecule:**hypothetical protein; **PDBTitle:** ankyrin repeat protein from leishmania major |
| 51 | c4b93B_ | Alignment | not modelled | 99.8 | 29 | **PDB header:**exocytosis **Chain:** B: **PDB Molecule:**ankyrin repeat domain-containing protein 27; **PDBTitle:** complex of vamp7 cytoplasmic domain with 2nd ankyrin repeat2 domain of varp |
| 52 | c4n5qA_ | Alignment | not modelled | 99.8 | 12 | **PDB header:**protein binding **Chain:** A: **PDB Molecule:**transient receptor potential cation channel subfamily v **PDBTitle:** crystal structure of the n-terminal ankyrin repeat domain of trpv3 |
| 53 | c4gmrA_ | Alignment | not modelled | 99.8 | 48 | **PDB header:**de novo protein **Chain:** A: **PDB Molecule:**or266 de novo protein; **PDBTitle:** crystal structure of engineered protein. northeast structural genomics2 consortium target or266. |
| 54 | c2zgdA_ | Alignment | not modelled | 99.8 | 28 | **PDB header:**de novo protein **Chain:** A: **PDB Molecule:**3 repeat synthetic ankyrin; **PDBTitle:** asn-hydroxylation stabilises the ankyrin repeat domain fold |
| | | | | | | **PDB header:**protein binding |

| | | | | | | |
|---|---|---|---|---|---|---|
| 55 | c3c5rB_ | Alignment | not modelled | 99.8 | 28 | **Chain:** B: **PDB Molecule:**brca1-associated ring domain protein 1; **PDBTitle:** crystal structure of the bard1 ankyrin repeat domain and its2 functional consequences |
| 56 | d1blxb_ | Alignment | not modelled | 99.8 | 33 | **Fold:**beta-hairpin-alpha-hairpin repeat **Superfamily:**Ankyrin repeat **Family:**Ankyrin repeat |
| 57 | d2a5ea_ | Alignment | not modelled | 99.8 | 27 | **Fold:**beta-hairpin-alpha-hairpin repeat **Superfamily:**Ankyrin repeat **Family:**Ankyrin repeat |
| 58 | c2l6bA_ | Alignment | not modelled | 99.8 | 32 | **PDB header:**de novo protein **Chain:** A: **PDB Molecule:**nr1c; **PDBTitle:** nrc consensus ankyrin repeat protein solution structure |
| 59 | c3jxiA_ | Alignment | not modelled | 99.8 | 11 | **PDB header:**membrane protein **Chain:** A: **PDB Molecule:**vanilloid receptor-related osmotically activated channel **PDBTitle:** crystal structure of the chicken trpv4 ankyrin repeat domain |
| 60 | c2rfaA_ | Alignment | not modelled | 99.8 | 24 | **PDB header:**membrane protein **Chain:** A: **PDB Molecule:**transient receptor potential cation channel subfamily v **PDBTitle:** crystal structure of the mouse trpv6 ankyrin repeat domain |
| 61 | c1mj0A_ | Alignment | not modelled | 99.8 | 29 | **PDB header:**de novo protein **Chain:** A: **PDB Molecule:**sank e3_5 protein; **PDBTitle:** sank e3_5: an artificial ankyrin repeat protein |
| 62 | c3twtD_ | Alignment | not modelled | 99.8 | 31 | **PDB header:**signaling protein/peptide **Chain:** D: **PDB Molecule:**tankyrase-2; **PDBTitle:** crystal structure of arc4 from human tankyrase 2 in complex with2 peptide from human mcl1 (chimeric peptide) |
| 63 | c2kbxA_ | Alignment | not modelled | 99.8 | 23 | **PDB header:**cell adhesion **Chain:** A: **PDB Molecule:**integrin-linked protein kinase; **PDBTitle:** solution structure of ilk-pinch complex |
| 64 | c4bszB_ | Alignment | not modelled | 99.8 | 24 | **PDB header:**rna binding protein **Chain:** B: **PDB Molecule:**ankyrin repeat-containing protein yar1; **PDBTitle:** crystal structure of the yeast ribosomal protein rps3 in complex2 with its chaperone yar1 |
| 65 | c3v31A_ | Alignment | not modelled | 99.8 | 26 | **PDB header:**protein binding **Chain:** A: **PDB Molecule:**ankyrin repeat family a protein 2; **PDBTitle:** crystal structure of the peptide bound complex of the ankyrin repeat2 domains of human ankra2 |
| 66 | c2jabC_ | Alignment | not modelled | 99.8 | 26 | **PDB header:**de novo protein **Chain:** C: **PDB Molecule:**h10-2-g3; **PDBTitle:** a designed ankyrin repeat protein evolved to picomolar2 affinity to her2 |
| 67 | c2xenA_ | Alignment | not modelled | 99.8 | 33 | **PDB header:**de novo protein **Chain:** A: **PDB Molecule:**ni1c mut4; **PDBTitle:** structural determinants for improved thermal stability of2 designed ankyrin repeat proteins with a redesigned c-3 capping module. |
| 68 | d1bi7b_ | Alignment | not modelled | 99.8 | 17 | **Fold:**beta-hairpin-alpha-hairpin repeat **Superfamily:**Ankyrin repeat **Family:**Ankyrin repeat |
| 69 | c2f37B_ | Alignment | not modelled | 99.7 | 14 | **PDB header:**membrane protein **Chain:** B: **PDB Molecule:**transient receptor potential cation channel **PDBTitle:** crystal structure of the ankyrin repeat domain of human2 trpv2 |
| 70 | d1oy3d_ | Alignment | not modelled | 99.7 | 29 | **Fold:**beta-hairpin-alpha-hairpin repeat **Superfamily:**Ankyrin repeat **Family:**Ankyrin repeat |
| 71 | c1oy3D_ | Alignment | not modelled | 99.7 | 29 | **PDB header:**dna binding protein **Chain:** D: **PDB Molecule:**transcription factor inhibitor i-kappa-b-beta; **PDBTitle:** crystal structure of an ikbbeta/nf-kb p65 homodimer complex |
| 72 | c4tumD_ | Alignment | not modelled | 99.7 | 29 | **PDB header:**protein binding **Chain:** D: **PDB Molecule:**ankyrin repeat domain-containing protein 2; **PDBTitle:** crystal structure of ankyrin repeat domain of akr2 |
| 73 | d1awcb_ | Alignment | not modelled | 99.7 | 26 | **Fold:**beta-hairpin-alpha-hairpin repeat **Superfamily:**Ankyrin repeat **Family:**Ankyrin repeat |
| 74 | c1ympB_ | Alignment | not modelled | 99.7 | 23 | **PDB header:**transcription **Chain:** B: **PDB Molecule:**notch 1 protein; **PDBTitle:** the crystal structure of a partial mouse notch-1 ankyrin2 domain: repeats 4 through 7 preserve an ankyrin fold |
| 75 | c1ympA_ | Alignment | not modelled | 99.7 | 23 | **PDB header:**transcription **Chain:** A: **PDB Molecule:**notch 1 protein; **PDBTitle:** the crystal structure of a partial mouse notch-1 ankyrin2 domain: repeats 4 through 7 preserve an ankyrin fold |
| 76 | c1n0qB_ | Alignment | not modelled | 99.7 | 38 | **PDB header:**structural protein **Chain:** B: **PDB Molecule:**3 ankyrin repeats; **PDBTitle:** 3ank: a designed ankyrin repeat protein with three identical consensus2 repeats |
| 77 | c4cz2D_ | Alignment | not modelled | 99.7 | 25 | **PDB header:**signaling protein **Chain:** D: **PDB Molecule:**ankyrin repeat domain-containing protein 27; **PDBTitle:** complex of human varp-ankrd1 with rab32-gppcp. selenomet derivative. |
| 78 | c2nyjA_ | Alignment | not modelled | 99.7 | 19 | **PDB header:**transport protein **Chain:** A: **PDB Molecule:**transient receptor potential cation channel **PDBTitle:** crystal structure of the ankyrin repeat domain of trpv1 |
| 79 | c2rfmB_ | Alignment | not modelled | 99.7 | 28 | **PDB header:**protein binding **Chain:** B: **PDB Molecule:**putative ankyrin repeat protein tv1425; **PDBTitle:** structure of a thermophilic ankyrin repeat protein |
| | | | | | | **Fold:**beta-hairpin-alpha-hairpin repeat |

| | | | | | | |
|---|---|---|---|---|---|---|
| 80 | d1bd8a_ | Alignment | not modelled | 99.7 | 16 | **Superfamily:**Ankyrin repeat<br>**Family:**Ankyrin repeat |
| 81 | c2v5qC_ | Alignment | not modelled | 99.6 | 30 | **PDB header:**transferase<br>**Chain:** C: **PDB Molecule:**design ankyrin repeat protein;<br>**PDBTitle:** crystal structure of wild-type plk-1 kinase domain in2 complex with a selective darpin |
| 82 | d1ihba_ | Alignment | not modelled | 99.6 | 16 | **Fold:**beta-hairpin-alpha-hairpin repeat<br>**Superfamily:**Ankyrin repeat<br>**Family:**Ankyrin repeat |
| 83 | c3v30A_ | Alignment | not modelled | 99.6 | 26 | **PDB header:**protein binding<br>**Chain:** A: **PDB Molecule:**dna-binding protein rfxank;<br>**PDBTitle:** crystal structure of the peptide bound complex of the ankyrin repeat2 domains of human rfxank |
| 84 | c1n0rA_ | Alignment | not modelled | 99.4 | 43 | **PDB header:**structural protein<br>**Chain:** A: **PDB Molecule:**4 ankyrin repeats;<br>**PDBTitle:** 4ank: a designed ankyrin repeat protein with four identical2 consensus repeats |
| 85 | c4zhbA_ | Alignment | not modelled | 93.3 | 22 | **PDB header:**structural genomics, unknown function<br>**Chain:** A: **PDB Molecule:**ankyrin repeat-containing protein;<br>**PDBTitle:** n-terminal structure of ankyrin repeat-containing protein lega11 from2 legionella pneumophila |
| 86 | c2xumS_ | Alignment | not modelled | 88.7 | 39 | **PDB header:**oxidoreductase/peptide<br>**Chain:** S: **PDB Molecule:**asp-substrate peptide 2;<br>**PDBTitle:** factor inhibiting hif (fih) q239h mutant in complex with zn(ii), nog2 and asp-substrate peptide (20-mer) |
| 87 | c2y0iS_ | Alignment | not modelled | 68.0 | 36 | **PDB header:**oxidoreductase/peptide<br>**Chain:** S: **PDB Molecule:**tankyrase-2;<br>**PDBTitle:** factor inhibiting hif-1 alpha in complex with tankyrase-2 (tnks2)2 fragment peptide (21-mer) |
| 88 | c4qiwT_ | Alignment | not modelled | 17.6 | 21 | **PDB header:**transcription<br>**Chain:** T: **PDB Molecule:**dna-directed rna polymerase subunit k;<br>**PDBTitle:** crystal structure of euryarchaeal rna polymerase from thermococcus2 kodakarensis |
| 89 | c3ih9A_ | Alignment | not modelled | 15.4 | 7 | **PDB header:**hydrolase<br>**Chain:** A: **PDB Molecule:**salt-tolerant glutaminase;<br>**PDBTitle:** crystal structure analysis of mglu in its tris form |
| 90 | d1eqza_ | Alignment | not modelled | 10.0 | 12 | **Fold:**Histone-fold<br>**Superfamily:**Histone-fold<br>**Family:**Nucleosome core histones |
| 91 | c3sviA_ | Alignment | not modelled | 9.4 | 11 | **PDB header:**signaling protein<br>**Chain:** A: **PDB Molecule:**type iii effector hopab2;<br>**PDBTitle:** structure of the pto-binding domain of hoppmal generated by limited2 thermolysin digestion |
| 92 | c3hgkE_ | Alignment | not modelled | 6.4 | 10 | **PDB header:**transferase<br>**Chain:** E: **PDB Molecule:**effector protein hopab2;<br>**PDBTitle:** crystal structure of effect protein avrptob complexed with2 kinase pto |
| 93 | d1kx5c_ | Alignment | not modelled | 5.6 | 12 | **Fold:**Histone-fold<br>**Superfamily:**Histone-fold<br>**Family:**Nucleosome core histones |
| 94 | c1twcF_ | Alignment | not modelled | 5.1 | 8 | **PDB header:**transcription<br>**Chain:** F: **PDB Molecule:**dna-directed rna polymerases i, ii, and iii 23<br>**PDBTitle:** rna polymerase ii complexed with gtp |
