# Supplementary material for: Insights into the Structure, Function, and Ion-Mediated Signaling Pathways Transduced by Plant Integrin-Linked Kinases
Source: Front Plant Sci. 2017 Apr 3;8:376. doi: 10.3389/fpls.2017.00376 (PMC5376563; doi:10.3389/fpls.2017.00376)
Supplement: DATA S2 — Model and information associated with 3D structure prediction of ankyrin repeat domains of ILK1 to ILK6. [file Data_Sheet_2.ZIP › SDATA_2_ILKs_AR_SupplementalData/ILK4_ss_report.pdf]

Email	scp319@msstate.edu
Description	ILK4_AR__
Date	Fri Jul 29 15:31:48 BST 2016
Unique Job ID	941274f5f6a2028c

Secondary structure and disorder prediction

	1–60
Sequence	MTIKPKSPARFKLGRQSSLAPESRTPIDTLTEDEDDDLAAAATAGIGDPTIRLMYLANEG
Secondary structure	
SS confidence	
Disorder	? ? ? ? ? ? ? ? ? ? ????? ? ? ? ?
Disorder confidence	
Conserved Domain info	

	61–120
Sequence	DIDGINKMLDSGTNVDYRDIDARTALHVAACQGRTDVVELLLSRGAKVDTKDRWGSTPLA
Secondary structure	
SS confidence	
Disorder	?
Disorder confidence	
Conserved Domain info	

	121–168
Sequence	DAVYYKNHDVIKLLEKHGAKPTIAPMHVLTDKEVPEYEIHPTELDFSN
Secondary structure	
SS confidence	
Disorder	? ? ? ?
Disorder confidence	
Conserved Domain info	

Confidence Key

High(9) Low (0)

? Disordered (14%)

Alpha helix (47%)

Beta strand (2%)
