# Supplementary material for: Insights into the Structure, Function, and Ion-Mediated Signaling Pathways Transduced by Plant Integrin-Linked Kinases
Source: Front Plant Sci. 2017 Apr 3;8:376. doi: 10.3389/fpls.2017.00376 (PMC5376563; doi:10.3389/fpls.2017.00376)
Supplement: DATA S2 — Model and information associated with 3D structure prediction of ankyrin repeat domains of ILK1 to ILK6. [file Data_Sheet_2.ZIP › SDATA_2_ILKs_AR_SupplementalData/ILK5_hit_report.pdf]

Phyre²

Email	scp319@msstate.edu
Description	ILK5_AR__
Date	Fri Jul 29 15:32:12 BST 2016
Unique Job ID	e739a0c46e60c553

Detailed template information

#	Template	Alignment Coverage	3D Model	Confidence	% i.d.	Template Information
1	c4rlvA_	Alignment	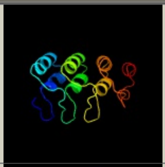	100.0	25	**PDB header:**structural protein<br>**Chain:** A: **PDB Molecule:**ankyrin-1, ankyrin-2;<br>**PDBTitle:** crystal structure of ankb 24 ankyrin repeats in complex with ankr2 autoinhibition segment
2	c4xd0A_	Alignment	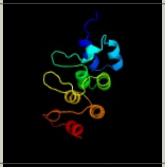	100.0	15	**PDB header:**transferase<br>**Chain:** A: **PDB Molecule:**tdp-3-aminoquinovose-n-formyltransferase;<br>**PDBTitle:** x-ray structure of the n-formyltransferase qdtf from providencia2 alcalifaciens
3	c4cj9A_	Alignment	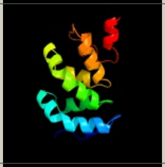	100.0	12	**PDB header:**transcription<br>**Chain:** A: **PDB Molecule:**burrh;<br>**PDBTitle:** burrh dna-binding protein from burkholderia rhizoxinica in2 its apo form
4	c5aq7B_	Alignment	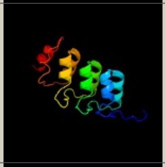	100.0	24	**PDB header:**chaperone<br>**Chain:** B: **PDB Molecule:**d12_db04v3;<br>**PDBTitle:** darpin-based crystallization chaperones exploit molecular geometry as2 a screening dimension in protein crystallography
5	c5d68C_	Alignment	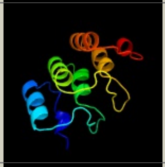	100.0	23	**PDB header:**signaling protein<br>**Chain:** C: **PDB Molecule:**krev interaction trapped protein 1;<br>**PDBTitle:** crystal structure of krit1 ard-ferm
6	c4oauC_	Alignment	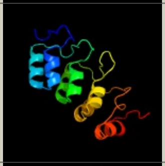	100.0	23	**PDB header:**hydrolase/rna<br>**Chain:** C: **PDB Molecule:**2-5a-dependent ribonuclease;<br>**PDBTitle:** complete human rnase l in complex with biological activators.
7	c1ycsB_	Alignment	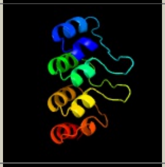	100.0	24	**PDB header:**complex (anti-oncogene/ankyrin repeats)<br>**Chain:** B: **PDB Molecule:**53bp2;<br>**PDBTitle:** p53-53bp2 complex
8	c5iwtA_	Alignment	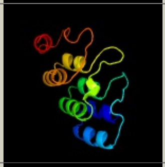	100.0	24	**PDB header:**transport protein<br>**Chain:** A: **PDB Molecule:**transient receptor potential cation channel subfamily v<br>**PDBTitle:** structure of transient receptor potential (trp) channel trpv6 in the2 presence of gadolinium
9	c5czyA_	Alignment	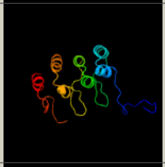	100.0	21	**PDB header:**transferase<br>**Chain:** A: **PDB Molecule:**legionella effector legas4;<br>**PDBTitle:** crystal structure of legas4
10	c5et1A_	Alignment	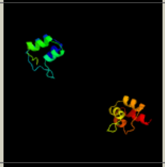	100.0	32	**PDB header:**protein binding/motor protein<br>**Chain:** A: **PDB Molecule:**espin;<br>**PDBTitle:** crystal structure of myo3b-arb1 in complex with espin1-ar
11	c3j9pD_	Alignment	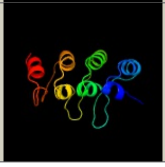	100.0	25	**PDB header:**transport protein<br>**Chain:** D: **PDB Molecule:**maltose-binding periplasmic protein, transient receptor<br>**PDBTitle:** structure of the trpa1 ion channel determined by electron cryo-2 microscopy

12	c4g8kA_	Alignment	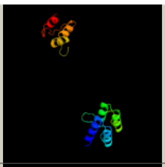	100.0	22	**PDB header:**hydrolase<br>**Chain:** A: **PDB Molecule:**2-5a-dependent ribonuclease;<br>**PDBTitle:** intact sensor domain of human rnase l in the inactive signaling state
13	c3utmA_	Alignment	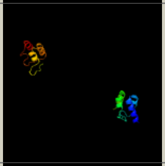	99.9	32	**PDB header:**transferase/signaling protein<br>**Chain:** A: **PDB Molecule:**tankyrase-1;<br>**PDBTitle:** crystal structure of a mouse tankyrase-axin complex
14	c3keaB_	Alignment	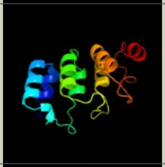	99.9	17	**PDB header:**viral protein<br>**Chain:** B: **PDB Molecule:**k1l;<br>**PDBTitle:** structure function studies of vaccinia virus host-range protein k12 reveal a novel ankyrin repeat interaction surface for k1s function
15	c4bepB_	Alignment	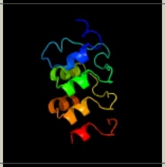	99.9	21	**PDB header:**transferase<br>**Chain:** B: **PDB Molecule:**phosphocholine transferase ankx;<br>**PDBTitle:** crystal structure of the legionella pneumophila fic domain-2 containing effector ankx protein (apo-form)
16	c1n11A_	Alignment	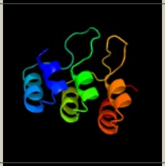	99.9	27	**PDB header:**structural protein<br>**Chain:** A: **PDB Molecule:**ankyrin;<br>**PDBTitle:** d34 region of human ankyrin-r and linker
17	d1n11a_	Alignment	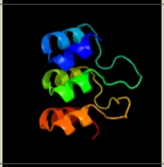	99.9	27	**Fold:**beta-hairpin-alpha-hairpin repeat<br>**Superfamily:**Ankyrin repeat<br>**Family:**Ankyrin repeat
18	c3j5pB_	Alignment	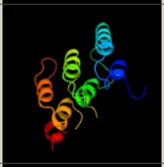	99.9	23	**PDB header:**transport protein<br>**Chain:** B: **PDB Molecule:**transient receptor potential cation channel subfamily v<br>**PDBTitle:** structure of trpv1 ion channel determined by single particle electron2 cryo-microscopy
19	d2ajaa1	Alignment	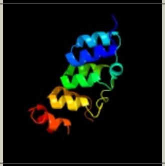	99.9	16	**Fold:**alpha-alpha superhelix<br>**Superfamily:**Pseudo ankyrin repeat-like<br>**Family:**Pseudo ankyrin repeat
20	c2xaiD_	Alignment	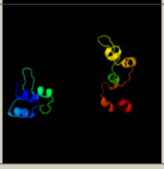	99.9	26	**PDB header:**transcription<br>**Chain:** D: **PDB Molecule:**ankyrin repeat and socs box protein 9;<br>**PDBTitle:** crystal structure of ankyrin repeat and socs box-containing2 protein 9 (asb9) in complex with elonginb and elonginc
21	d1sw6a_	Alignment	not modelled	99.9	13	**Fold:**beta-hairpin-alpha-hairpin repeat<br>**Superfamily:**Ankyrin repeat<br>**Family:**Ankyrin repeat
22	c1sw6A_	Alignment	not modelled	99.9	13	**PDB header:**transcription regulation<br>**Chain:** A: **PDB Molecule:**regulatory protein swi6;<br>**PDBTitle:** s. cerevisiae swi6 ankyrin-repeat fragment
23	d1s70b_	Alignment	not modelled	99.9	34	**Fold:**beta-hairpin-alpha-hairpin repeat<br>**Superfamily:**Ankyrin repeat<br>**Family:**Ankyrin repeat
24	c2fo1E_	Alignment	not modelled	99.9	20	**PDB header:**gene regulation/signalling protein/dna<br>**Chain:** E: **PDB Molecule:**lin-12 protein;<br>**PDBTitle:** crystal structure of the csl-notch-mastermind ternary2 complex bound to dna
25	c2ajaA_	Alignment	not modelled	99.9	16	**PDB header:**protein binding<br>**Chain:** A: **PDB Molecule:**ankyrin repeat family protein;<br>**PDBTitle:** x-ray structure of an ankyrin repeat family protein q5zsv02 from legionella pneumophila. northeast structural genomics3 consortium target lgr21.
26	c3t9kA_	Alignment	not modelled	99.9	18	**PDB header:**protein transport<br>**Chain:** A: **PDB Molecule:**arf-gap with coiled-coil, ank repeat and ph domain-<br>**PDBTitle:** crystal structure of acap1 c-portion mutant s554d fused with integrin2 beta1 peptide
27	c4o60A_	Alignment	not modelled	99.9	42	**PDB header:**de novo protein<br>**Chain:** A: **PDB Molecule:**ank-n5c-317;<br>**PDBTitle:** structure of ankyrin repeat protein
28	c4ot9A_	Alignment	not modelled	99.9	27	**PDB header:**transcription<br>**Chain:** A: **PDB Molecule:**nuclear factor nf-kappa-b p100 subunit;<br>**PDBTitle:** crystal structure of the c-terminal domain of p100/nf-kb2

29	c2b0oF_	Alignment	not modelled	99.9	20	**PDB header:**metal binding protein<br>**Chain:** F: **PDB Molecule:**uplc1;<br>**PDBTitle:** crystal structure of uplc1 gap domain
30	c1dcqA_	Alignment	not modelled	99.9	17	**PDB header:**metal binding protein<br>**Chain:** A: **PDB Molecule:**pyk2-associated protein beta;<br>**PDBTitle:** crystal structure of the arf-gap domain and ankyrin repeats2 of papbeta.
31	c3d9hA_	Alignment	not modelled	99.9	22	**PDB header:**structural protein, protein binding<br>**Chain:** A: **PDB Molecule:**cdna flj77766, highly similar to homo sapiens<br>**PDBTitle:** crystal structure of the splice variant of human asb92 (hasb9-2), an ankyrin repeat protein
32	c2vgeA_	Alignment	not modelled	99.9	22	**PDB header:**transcription<br>**Chain:** A: **PDB Molecule:**rela-associated inhibitor;<br>**PDBTitle:** crystal structure of the c-terminal region of human iaspp
33	d1uoha_	Alignment	not modelled	99.9	30	**Fold:**beta-hairpin-alpha-hairpin repeat<br>**Superfamily:**Ankyrin repeat<br>**Family:**Ankyrin repeat
34	c3lvrE_	Alignment	not modelled	99.9	21	**PDB header:**protein transport<br>**Chain:** E: **PDB Molecule:**arf-gap with sh3 domain, ank repeat and ph domain-<br>**PDBTitle:** the crystal structure of asap3 in complex with arf6 in transition2 state soaked with calcium
35	c5d66B_	Alignment	not modelled	99.9	37	**PDB header:**structural genomics, unknown function<br>**Chain:** B: **PDB Molecule:**uncharacterized protein;<br>**PDBTitle:** crystal structure of an ankyrin repeat domain (abaye2397) from2 acinetobacter baumannii aye at 1.00 a resolution
36	d1wdya_	Alignment	not modelled	99.9	36	**Fold:**beta-hairpin-alpha-hairpin repeat<br>**Superfamily:**Ankyrin repeat<br>**Family:**Ankyrin repeat
37	c3jueA_	Alignment	not modelled	99.9	24	**PDB header:**protein transport/endocytosis<br>**Chain:** A: **PDB Molecule:**arfgap with coiled-coil, ank repeat and ph domain-<br>**PDBTitle:** crystal structure of arfgap and ank repeat domain of acap1
38	c3ui2A_	Alignment	not modelled	99.9	26	**PDB header:**transport protein<br>**Chain:** A: **PDB Molecule:**signal recognition particle 43 kda protein, chloroplastic;<br>**PDBTitle:** crystal structure of the cpsrp54 tail bound to cpsrp43
39	c3eu9B_	Alignment	not modelled	99.9	26	**PDB header:**protein binding, methyl-lysine-binding p<br>**Chain:** B: **PDB Molecule:**huntingtin-interacting protein 14;<br>**PDBTitle:** the ankyrin repeat domain of huntingtin interacting protein 14
40	d1dcqa1	Alignment	not modelled	99.9	19	**Fold:**beta-hairpin-alpha-hairpin repeat<br>**Superfamily:**Ankyrin repeat<br>**Family:**Ankyrin repeat
41	c4uucA_	Alignment	not modelled	99.9	29	**PDB header:**signaling protein<br>**Chain:** A: **PDB Molecule:**ankyrin repeat and socs box protein 11;<br>**PDBTitle:** crystal structure of human asb11 ankyrin repeat domain
42	d1ycsb1	Alignment	not modelled	99.9	24	**Fold:**beta-hairpin-alpha-hairpin repeat<br>**Superfamily:**Ankyrin repeat<br>**Family:**Ankyrin repeat
43	c5an8B_	Alignment	not modelled	99.9	26	**PDB header:**transport protein<br>**Chain:** B: **PDB Molecule:**trpv2;<br>**PDBTitle:** cryo-electron microscopy structure of rabbit trpv2 ion channel
44	d1k1aa_	Alignment	not modelled	99.9	27	**Fold:**beta-hairpin-alpha-hairpin repeat<br>**Superfamily:**Ankyrin repeat<br>**Family:**Ankyrin repeat
45	d1ixva_	Alignment	not modelled	99.9	27	**Fold:**beta-hairpin-alpha-hairpin repeat<br>**Superfamily:**Ankyrin repeat<br>**Family:**Ankyrin repeat
46	c3ehrB_	Alignment	not modelled	99.9	23	**PDB header:**signaling protein<br>**Chain:** B: **PDB Molecule:**osteoclast-stimulating factor 1;<br>**PDBTitle:** crystal structure of human osteoclast stimulating factor
47	c2rfaA_	Alignment	not modelled	99.9	30	**PDB header:**membrane protein<br>**Chain:** A: **PDB Molecule:**transient receptor potential cation channel subfamily v<br>**PDBTitle:** crystal structure of the mouse trpv6 ankyrin repeat domain
48	c3ljnA_	Alignment	not modelled	99.9	18	**PDB header:**unknown function<br>**Chain:** A: **PDB Molecule:**hypothetical protein;<br>**PDBTitle:** ankyrin repeat protein from leishmania major
49	c3deoA_	Alignment	not modelled	99.9	24	**PDB header:**protein transport, membrane protein<br>**Chain:** A: **PDB Molecule:**signal recognition particle 43 kda protein;<br>**PDBTitle:** structural basis for specific substrate recognition by the2 chloroplast signal recognition particle protein cpsrp43
50	c3b7bB_	Alignment	not modelled	99.9	21	**PDB header:**transferase<br>**Chain:** B: **PDB Molecule:**euchromatic histone-lysine n-methyltransferase 1;<br>**PDBTitle:** euhmt1 (glp) ankyrin repeat domain (structure 1)
51	c5cerD_	Alignment	not modelled	99.9	37	**PDB header:**immune system<br>**Chain:** D: **PDB Molecule:**bd3460;<br>**PDBTitle:** bd0816 predatory endopeptidase from bdellovibrio bacteriovorus in2 complex with immunity protein bd3460
52	d1oy3d_	Alignment	not modelled	99.9	43	**Fold:**beta-hairpin-alpha-hairpin repeat<br>**Superfamily:**Ankyrin repeat<br>**Family:**Ankyrin repeat
53	c1oy3D_	Alignment	not modelled	99.9	43	**PDB header:**dna binding protein<br>**Chain:** D: **PDB Molecule:**transcription factor inhibitor i-kappa-b-beta;<br>**PDBTitle:** crystal structure of an ikbbeta/nf-kb p65 homodimer complex
						**PDB header:**structural protein

54	c3hraA_	Alignment	not modelled	99.9	27	**Chain:** A: **PDB Molecule:**ankyrin repeat family protein;<br>**PDBTitle:** crystal structure of ef0377 an ankyrin repeat protein
55	d1myoa_	Alignment	not modelled	99.9	23	**Fold:**beta-hairpin-alpha-hairpin repeat<br>**Superfamily:**Ankyrin repeat<br>**Family:**Ankyrin repeat
56	c2kbxA_	Alignment	not modelled	99.9	28	**PDB header:**cell adhesion<br>**Chain:** A: **PDB Molecule:**integrin-linked protein kinase;<br>**PDBTitle:** solution structure of ilk-pinch complex
57	c4hbdA_	Alignment	not modelled	99.9	20	**PDB header:**protein binding<br>**Chain:** A: **PDB Molecule:**kn motif and ankyrin repeat domain-containing protein 2;<br>**PDBTitle:** crystal structure of kank2 ankyrin repeats
58	c3v31A_	Alignment	not modelled	99.9	30	**PDB header:**protein binding<br>**Chain:** A: **PDB Molecule:**ankyrin repeat family a protein 2;<br>**PDBTitle:** crystal structure of the peptide bound complex of the ankyrin repeat2 domains of human ankra2
59	d1iknd_	Alignment	not modelled	99.9	22	**Fold:**beta-hairpin-alpha-hairpin repeat<br>**Superfamily:**Ankyrin repeat<br>**Family:**Ankyrin repeat
60	d2a5ea_	Alignment	not modelled	99.9	20	**Fold:**beta-hairpin-alpha-hairpin repeat<br>**Superfamily:**Ankyrin repeat<br>**Family:**Ankyrin repeat
61	c2zgdA_	Alignment	not modelled	99.8	34	**PDB header:**de novo protein<br>**Chain:** A: **PDB Molecule:**3 repeat synthetic ankyrin;<br>**PDBTitle:** asn-hydroxylation stabilises the ankyrin repeat domain fold
62	d1blxb_	Alignment	not modelled	99.8	27	**Fold:**beta-hairpin-alpha-hairpin repeat<br>**Superfamily:**Ankyrin repeat<br>**Family:**Ankyrin repeat
63	c4n5qA_	Alignment	not modelled	99.8	26	**PDB header:**protein binding<br>**Chain:** A: **PDB Molecule:**transient receptor potential cation channel subfamily v<br>**PDBTitle:** crystal structure of the n-terminal ankyrin repeat domain of trpv3
64	c3t8kB_	Alignment	not modelled	99.8	17	**PDB header:**structural genomics, unknown function<br>**Chain:** B: **PDB Molecule:**uncharacterized protein;<br>**PDBTitle:** the crystal structure of a functionally unknown protein lebu_0176 from2 leptotrichia buccalis c-1013-b
65	c2xenA_	Alignment	not modelled	99.8	36	**PDB header:**de novo protein<br>**Chain:** A: **PDB Molecule:**ni1c mut4;<br>**PDBTitle:** structural determinants for improved thermal stability of2 designed ankyrin repeat proteins with a redesigned c-3 capping module.
66	c1n0qB_	Alignment	not modelled	99.8	39	**PDB header:**structural protein<br>**Chain:** B: **PDB Molecule:**3 ankyrin repeats;<br>**PDBTitle:** 3ank: a designed ankyrin repeat protein with three identical consensus2 repeats
67	c5cbol_	Alignment	not modelled	99.8	30	**PDB header:**protein binding<br>**Chain:** I: **PDB Molecule:**mbp3-16,immunoglobulin g-binding protein a;<br>**PDBTitle:** fusion protein of mbp3-16 and b4 domain of protein a from2 staphylococcal aureus
68	d1ot8a_	Alignment	not modelled	99.8	29	**Fold:**beta-hairpin-alpha-hairpin repeat<br>**Superfamily:**Ankyrin repeat<br>**Family:**Ankyrin repeat
69	c4gmrA_	Alignment	not modelled	99.8	41	**PDB header:**de novo protein<br>**Chain:** A: **PDB Molecule:**or266 de novo protein;<br>**PDBTitle:** crystal structure of engineered protein. northeast structural genomics2 consortium target or266.
70	c2f8xK_	Alignment	not modelled	99.8	29	**PDB header:**transcription/dna<br>**Chain:** K: **PDB Molecule:**neurogenic locus notch homolog protein 1;<br>**PDBTitle:** crystal structure of activated notch, csl and maml on hes-12 promoter dna sequence
71	c4b93B_	Alignment	not modelled	99.8	29	**PDB header:**exocytosis<br>**Chain:** B: **PDB Molecule:**ankyrin repeat domain-containing protein 27;<br>**PDBTitle:** complex of vamp7 cytoplasmic domain with 2nd ankyrin repeat2 domain of varp
72	c4bszB_	Alignment	not modelled	99.8	22	**PDB header:**rna binding protein<br>**Chain:** B: **PDB Molecule:**ankyrin repeat-containing protein yar1;<br>**PDBTitle:** crystal structure of the yeast ribosomal protein rps3 in complex2 with its chaperone yar1
73	c2l6bA_	Alignment	not modelled	99.8	34	**PDB header:**de novo protein<br>**Chain:** A: **PDB Molecule:**nr1c;<br>**PDBTitle:** nrc consensus ankyrin repeat protein solution structure
74	d2fo1e1	Alignment	not modelled	99.8	24	**Fold:**beta-hairpin-alpha-hairpin repeat<br>**Superfamily:**Ankyrin repeat<br>**Family:**Ankyrin repeat
75	c3c5rB_	Alignment	not modelled	99.8	29	**PDB header:**protein binding<br>**Chain:** B: **PDB Molecule:**brca1-associated ring domain protein 1;<br>**PDBTitle:** crystal structure of the bard1 ankyrin repeat domain and its2 functional consequences
76	d1bi7b_	Alignment	not modelled	99.8	26	**Fold:**beta-hairpin-alpha-hairpin repeat<br>**Superfamily:**Ankyrin repeat<br>**Family:**Ankyrin repeat
77	d1ihba_	Alignment	not modelled	99.8	27	**Fold:**beta-hairpin-alpha-hairpin repeat<br>**Superfamily:**Ankyrin repeat<br>**Family:**Ankyrin repeat
78	c4tumD_	Alignment	not modelled	99.8	37	**PDB header:**protein binding<br>**Chain:** D: **PDB Molecule:**ankyrin repeat domain-containing protein 2;<br>**PDBTitle:** crystal structure of ankyrin repeat domain of akr2
79	c3v30A_	Alignment	not modelled	99.8	33	**PDB header:**protein binding<br>**Chain:** A: **PDB Molecule:**dna-binding protein rfxank;<br>**PDBTitle:** crystal structure of the peptide bound complex of the ankyrin repeat2 domains of human rfxank

80	d1bd8a_	Alignment	not modelled	99.8	33	**Fold:**beta-hairpin-alpha-hairpin repeat **Superfamily:**Ankyrin repeat **Family:**Ankyrin repeat
81	c1mj0A_	Alignment	not modelled	99.8	47	**PDB header:**de novo protein **Chain:** A: **PDB Molecule:**sank e3_5 protein; **PDBTitle:** sank e3_5: an artificial ankyrin repeat protein
82	c5ja4D_	Alignment	not modelled	99.8	28	**PDB header:**chaperone **Chain:** D: **PDB Molecule:**tonsoku-like protein; **PDBTitle:** crystal structure of human tonsl and mcm2 hbds binding to a histone2 h3-h4 tetramer
83	c2v5qC_	Alignment	not modelled	99.8	35	**PDB header:**transferase **Chain:** C: **PDB Molecule:**design ankyrin repeat protein; **PDBTitle:** crystal structure of wild-type plk-1 kinase domain in2 complex with a selective darpin
84	c2jabC_	Alignment	not modelled	99.8	35	**PDB header:**de novo protein **Chain:** C: **PDB Molecule:**h10-2-g3; **PDBTitle:** a designed ankyrin repeat protein evolved to picomolar2 affinity to her2
85	c2rfmB_	Alignment	not modelled	99.8	30	**PDB header:**protein binding **Chain:** B: **PDB Molecule:**putative ankyrin repeat protein tv1425; **PDBTitle:** structure of a thermophilic ankyrin repeat protein
86	c4cz2D_	Alignment	not modelled	99.7	30	**PDB header:**signaling protein **Chain:** D: **PDB Molecule:**ankyrin repeat domain-containing protein 27; **PDBTitle:** complex of human varp-ankrd1 with rab32-gppcp. selenomet derivative.
87	d1awcb_	Alignment	not modelled	99.7	33	**Fold:**beta-hairpin-alpha-hairpin repeat **Superfamily:**Ankyrin repeat **Family:**Ankyrin repeat
88	c3twtD_	Alignment	not modelled	99.7	27	**PDB header:**signaling protein/peptide **Chain:** D: **PDB Molecule:**tankyrase-2; **PDBTitle:** crystal structure of arc4 from human tankyrase 2 in complex with2 peptide from human mcl1 (chimeric peptide)
89	c3jxiA_	Alignment	not modelled	99.7	15	**PDB header:**membrane protein **Chain:** A: **PDB Molecule:**vanilloid receptor-related osmotically activated channel **PDBTitle:** crystal structure of the chicken trpv4 ankyrin repeat domain
90	c2f37B_	Alignment	not modelled	99.7	15	**PDB header:**membrane protein **Chain:** B: **PDB Molecule:**transient receptor potential cation channel **PDBTitle:** crystal structure of the ankyrin repeat domain of human2 trpv2
91	c1ympB_	Alignment	not modelled	99.6	26	**PDB header:**transcription **Chain:** B: **PDB Molecule:**notch 1 protein; **PDBTitle:** the crystal structure of a partial mouse notch-1 ankyrin2 domain: repeats 4 through 7 preserve an ankyrin fold
92	c1n0rA_	Alignment	not modelled	99.6	42	**PDB header:**structural protein **Chain:** A: **PDB Molecule:**4 ankyrin repeats; **PDBTitle:** 4ank: a designed ankyrin repeat protein with four identical2 consensus repeats
93	c1ympA_	Alignment	not modelled	99.6	26	**PDB header:**transcription **Chain:** A: **PDB Molecule:**notch 1 protein; **PDBTitle:** the crystal structure of a partial mouse notch-1 ankyrin2 domain: repeats 4 through 7 preserve an ankyrin fold
94	c2nyjA_	Alignment	not modelled	99.6	16	**PDB header:**transport protein **Chain:** A: **PDB Molecule:**transient receptor potential cation channel **PDBTitle:** crystal structure of the ankyrin repeat domain of trpv1
95	c4zhbA_	Alignment	not modelled	94.4	20	**PDB header:**structural genomics, unknown function **Chain:** A: **PDB Molecule:**ankyrin repeat-containing protein; **PDBTitle:** n-terminal structure of ankyrin repeat-containing protein lega11 from2 legionella pneumophila
96	c5is0E_	Alignment	not modelled	76.8	18	**PDB header:**transport protein **Chain:** E: **PDB Molecule:**transient receptor potential cation channel subfamily v **PDBTitle:** structure of trpv1 in complex with capsazepine, determined in lipid2 nanodisc
97	c2xumS_	Alignment	not modelled	75.3	33	**PDB header:**oxidoreductase/peptide **Chain:** S: **PDB Molecule:**asp-substrate peptide 2; **PDBTitle:** factor inhibiting hif (fih) q239h mutant in complex with zn(ii), nog2 and asp-substrate peptide (20-mer)
98	c2y0iS_	Alignment	not modelled	31.1	42	**PDB header:**oxidoreductase/peptide **Chain:** S: **PDB Molecule:**tankyrase-2; **PDBTitle:** factor inhibiting hif-1 alpha in complex with tankyrase-2 (tnks2)2 fragment peptide (21-mer)
99	c3ih9A_	Alignment	not modelled	28.2	13	**PDB header:**hydrolase **Chain:** A: **PDB Molecule:**salt-tolerant glutaminase; **PDBTitle:** crystal structure analysis of mglu in its tris form
