# Supplementary material for: Insights into the Structure, Function, and Ion-Mediated Signaling Pathways Transduced by Plant Integrin-Linked Kinases
Source: Front Plant Sci. 2017 Apr 3;8:376. doi: 10.3389/fpls.2017.00376 (PMC5376563; doi:10.3389/fpls.2017.00376)
Supplement: DATA S2 — Model and information associated with 3D structure prediction of ankyrin repeat domains of ILK1 to ILK6. [file Data_Sheet_2.ZIP › SDATA_2_ILKs_AR_SupplementalData/ILK5_ss_report.pdf]

Phyre²

Email	scp319@msstate.edu
Description	ILK5_AR__
Date	Fri Jul 29 15:32:12 BST 2016
Unique Job ID	e739a0c46e60c553

Secondary structure and disorder prediction

1 . . . . 10 . . . . 20 . . . . 30 . . . . 40 . . . . 50 60

Sequence: M E E D Y Q Q P R F T I G R Q S S M A P E K I P E P S V H S E E E V F E D G E E I D G G V R L M Y L A N E G D I E G I K

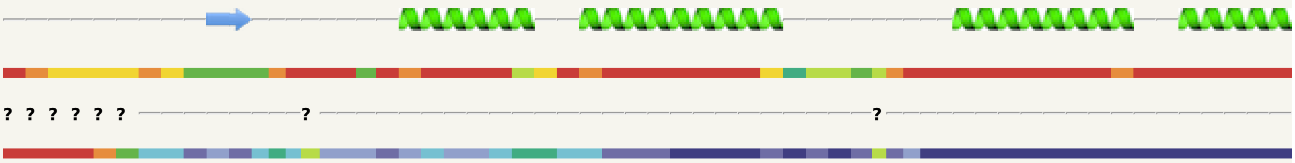

Secondary structure / SS confidence / Disorder / Disorder confidence / Conserved Domain info

Disorder: ? ? ? ? ? ? ? ?

. . . . 70 . . . . 80 . . . . 90 . . . . 100 . . . . 110 120

Sequence: E L I D S G I D A N Y R D I D D R T A L H V A A C Q G L K D V V E L L L D R K A E V D P K D R W G S T P F A D A I F Y K

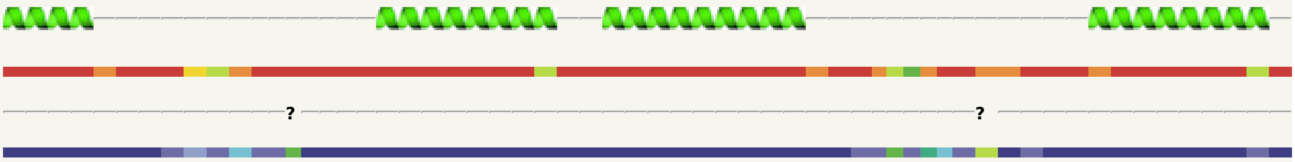

Secondary structure / SS confidence / Disorder / Disorder confidence / Conserved Domain info

Disorder: ? ?

. . . . 130 . . . . 140 . . . . 150 . . . . 160 .

Sequence: N I D V I K I L E I H G A K H P M A P M H V K T A R E V P E Y E I N P S E L D F T

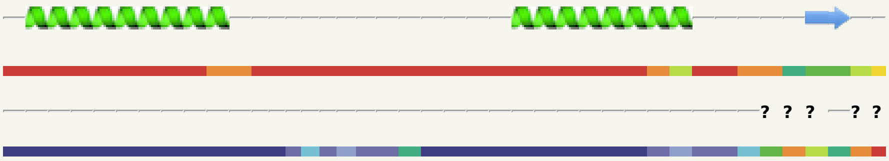

Secondary structure / SS confidence / Disorder / Disorder confidence / Conserved Domain info

Disorder: ? ? ? ? ?

Confidence Key

High(9) … Low (0)

? Disordered (9%)

Alpha helix (46%)

Beta strand (2%)
