# Supplementary figures and images for: Insights into the Structure, Function, and Ion-Mediated Signaling Pathways Transduced by Plant Integrin-Linked Kinases
Source: Front Plant Sci. 2017 Apr 3;8:376. doi: 10.3389/fpls.2017.00376 (PMC5376563; doi:10.3389/fpls.2017.00376)

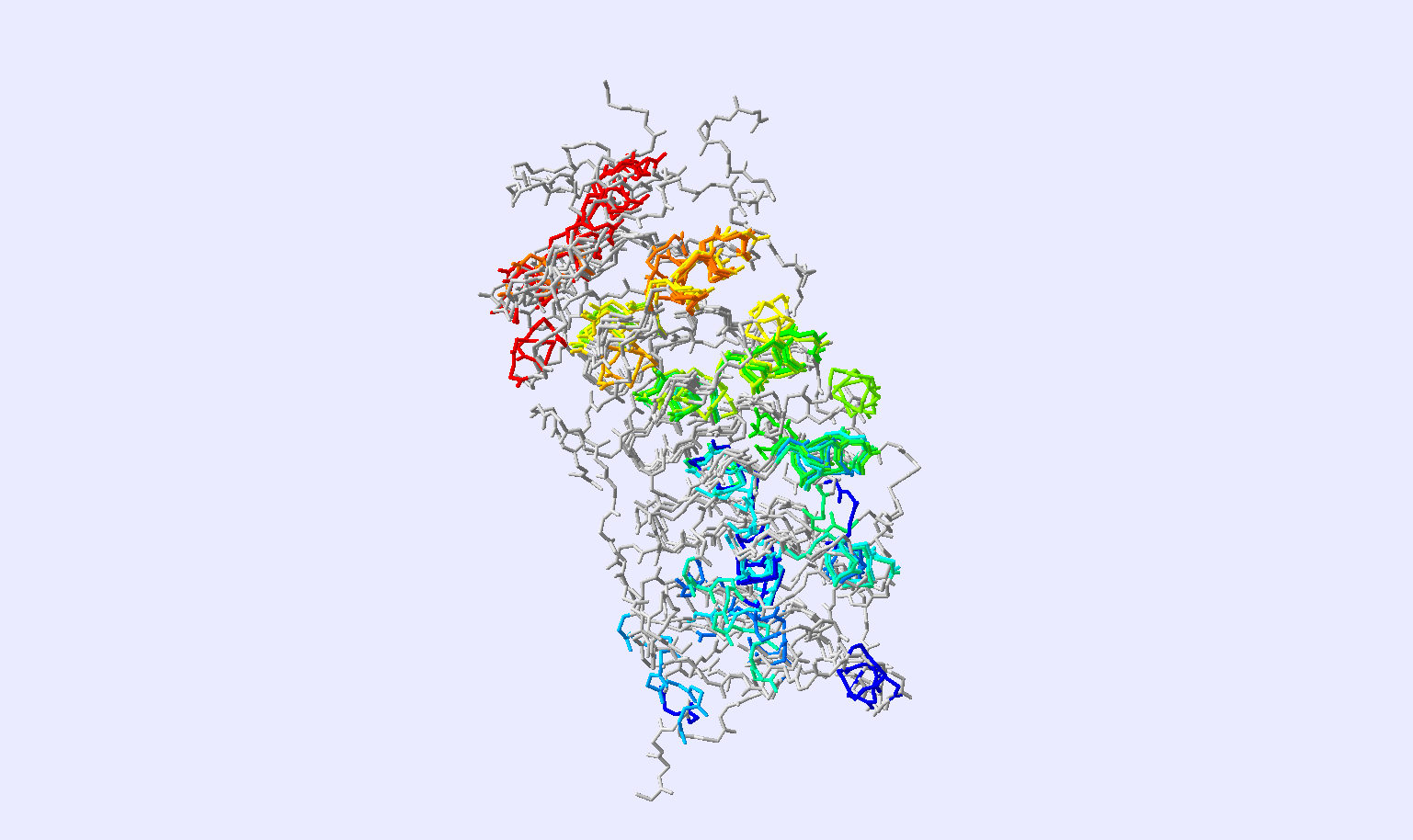

Supplement: DATA S3 — Information associated with superposition of the ankyrin repeat domains of ILK1 to ILK6. [file Data_Sheet_3.ZIP › SDATA_3.SS_SPDV_ILKs/Superposition_ILK1_final.png]
